# Genomic analyses reveal distinct genetic architectures and selective pressures in buffaloes

**DOI:** 10.1093/gigascience/giz166

**Published:** 2020-02-21

**Authors:** Ting Sun, Jiafei Shen, Alessandro Achilli, Ningbo Chen, Qiuming Chen, Ruihua Dang, Zhuqing Zheng, Hucai Zhang, Xiaoming Zhang, Shaoqiang Wang, Tao Zhang, Hongzhao Lu, Yun Ma, Yutang Jia, Marco Rosario Capodiferro, Yongzhen Huang, Xianyong Lan, Hong Chen, Yu Jiang, Chuzhao Lei

**Affiliations:** 1 Key Laboratory of Animal Genetics, Breeding and Reproduction of Shaanxi Province, College of Animal Science and Technology, Northwest A&F University, Yangling, Shaanxi 712100, China; 2 Dipartimento di Biologia e Biotecnologie “L. Spallanzani,” Università di Pavia, Pavia 27100, Italy; 3 Key Laboratory of Plateau Lake Ecology and Environment Change, Yunnan University, Kunming 650504, China; 4 State Key Laboratory of Genetic Resources and Evolution, Kunming Institute of Zoology, Chinese Academy of Sciences, Kunming 650223, China; 5 School of Bioscience and Engineering, Shaanxi University of Technology, Hanzhong, Shaanxi 723000, China; 6 Agricultural College, Ningxia University, Yinchuan 750021, China; 7 Institute of Animal Science and Veterinary Medicine, Anhui Academy of Agriculture Science, Hefei 230001, China

**Keywords:** buffalo, whole-genome resequencing, genetic history, selection

## Abstract

**Background:**

The domestic buffalo (*Bubalus bubalis*) is an essential farm animal in tropical and subtropical regions, whose genomic diversity is yet to be fully discovered.

**Results:**

In this study, we describe the demographic events and selective pressures of buffalo by analyzing 121 whole genomes (98 newly reported) from 25 swamp and river buffalo breeds. Both uniparental and biparental markers were investigated to provide the final scenario. The ancestors of swamp and river buffalo diverged ∼0.23 million years ago and then experienced independent demographic histories. They were domesticated in different regions, the swamp buffalo at the border between southwest China and southeast Asia, while the river buffalo in south Asia. The domestic stocks migrated to other regions and further differentiated, as testified by (at least) 2 ancestral components identified in each subspecies. Different signals of selective pressures were also detected in these 2 types of buffalo. The swamp buffalo, historically used as a draft animal, shows selection signatures in genes associated with the nervous system, while in river dairy breeds, genes under selection are related to heat stress and immunity.

**Conclusions:**

Our findings substantially expand the catalogue of genetic variants in buffalo and reveal new insights into the evolutionary history and distinct selective pressures in river and swamp buffalo.

## Background

The domestic buffalo is an important farm animal in tropical and subtropical regions, which can provide milk, meat, and draught power for rice cultivation. The domestic buffalo can be divided into 2 types: swamp and river buffalo. These 2 types show differences concerning body size, outward appearance, biological characteristics, and chromosome karyotype (2n = 48 in swamp buffalo; 2n = 50 in river buffalo) [1, 2]. The swamp buffalo is mainly bred in extensive rural areas in northeast India, southeast Asia, and south China, while the river buffalo is distributed from western India to Mediterranean areas. Swamp buffalo was traditionally raised as a draught animal for rice cultivation, while the river type was mainly selected for milk production [3].

There is a large agreement on the common ancestor of river and swamp buffaloes, both descending from the wild Asian buffalo (*Bubalus arnee*) [3]. However, details on the domestication process and its consequences are still missing. The oldest domestic buffalo remains of southeast Asia were found in northern Thailand and dated to 2,900–2,300 years ago [4], while other archeozoological evidence is scarce [5]. Therefore, the genomic screening of current breeds was often used to clarify the overall scenario. In particular, both uniparental markers were initially studied [6–13], while further details were eventually provided by autosomal analyses [14]. The current data on river buffalo point to an initial domestication in the Indian subcontinent 6,300–4,600 years ago and a following migration westwards into southern Europe [6, 10, 14]. The swamp buffalo was probably domesticated in the China/Indochina border. Then, the original stocks migrated to other regions: northward to China and then bending southwards into the Philippines; southward initially across the Mekong, then to Sumatra (Mekong colonization), and finally eastwards to the rest of Indonesia [12–14].

Considering that the entire genome variation of buffalo was largely unexplored [15], we sequenced the whole genome of 98 buffaloes from 21 swamp and 4 river breeds (Supplementary Tables 1 and 2) with different geographic origins in order to fully describe the genomic diversity, population structure, and demographic history of this important livestock species and to reveal possible signs of natural and artificial selection.

## Data Description

**Figure 1:**
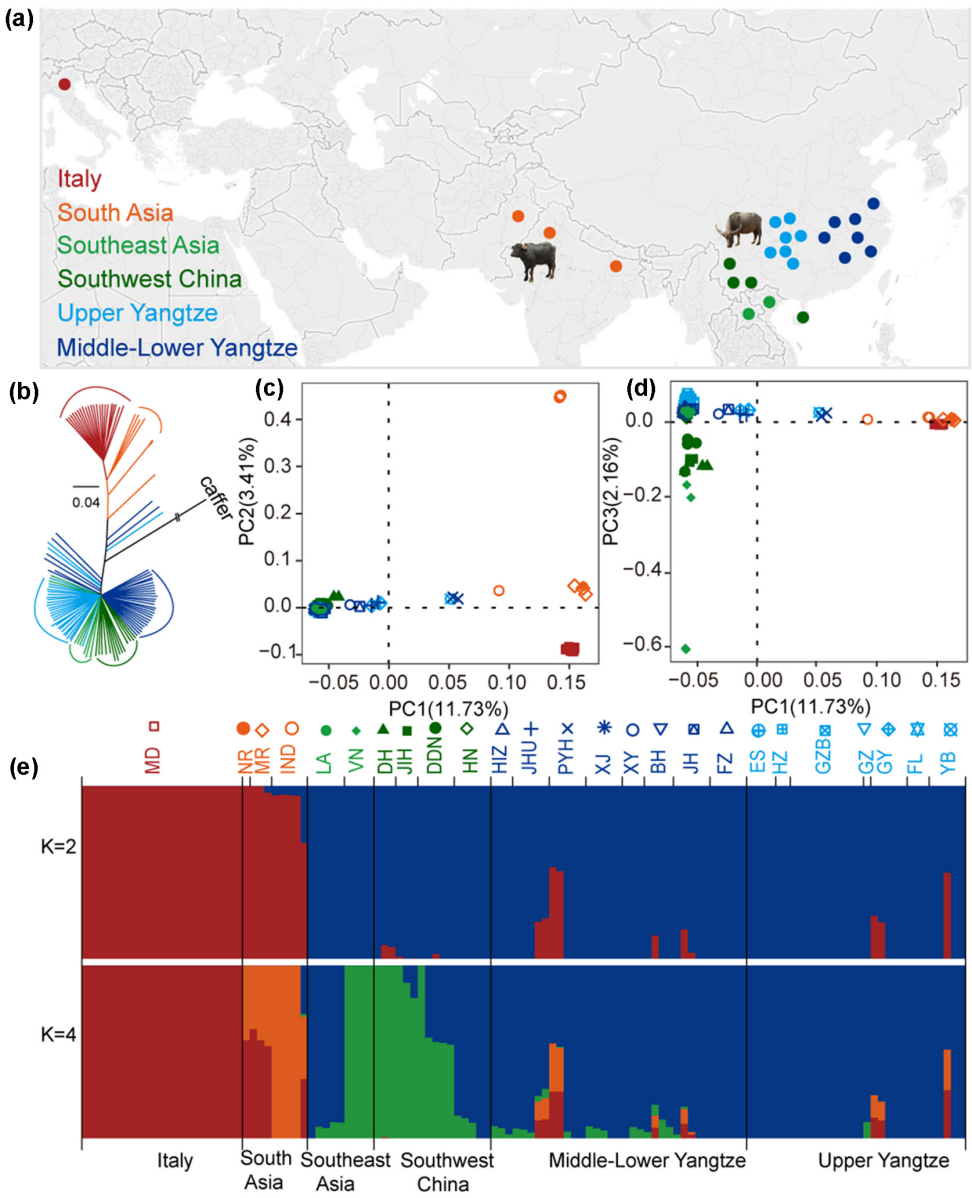
Population structure and relationships among buffaloes. (a) Geographic map indicating the origins of the buffalo breeds. (b) Neighbour-joining tree of buffaloes constructed using whole-genome autosomal SNP data. (c, d) Principal component analyses (PCA) showing PC1 against PC2 and PC1 against PC3, respectively. Each breed was labelled with different colors and shapes as shown in the top of Fig. 1e. (e) Genetic structure of buffalo breeds using ADMIXTURE program with *K* = 2, 4. Population acronyms are explained in Supplementary Tables 1 and 5.

We sampled a total of 98 domestic buffaloes (NCBI:txid89462) from different locations: China (81), Laos (5), Vietnam (4), and India (1 Nili-Ravi, 2 Murrah, and 5 Indian buffaloes). All genomic data have been submitted to the NCBI SRA under the BioProject accession number PRJNA547460. After adding 23 available genomes (Fig. 1a, Supplementary Tables 1 and 2) [15], the final dataset of 121 genomes was subdivided into 6 geographic groups: Upper Yangtze, Middle-Lower Yangtze, Southwest China, Southeast Asia , South Asia, and Italy (Fig.   1a, Supplementary Fig. 1).

## Analyses

### Population genetic structure and relationships

Neighbour-joining (NJ) trees, principal component analysis (PCA), and ADMIXTURE were used to explore the genetic relationships among the examined 121 buffaloes. The NJ tree, rooted with *Syncerus caffer*, showed a deep division between swamp and river buffaloes; then buffaloes from adjacent geographical regions form distinctive clades (Fig. 1b). The same geographic/genomic proximity was also confirmed by the maximum-likelihood (ML) tree (Supplementary Fig. 2). The PCA (Fig. 1c and d) showed that the first principal component (PC1) was driven by differences between swamp and river buffaloes. The PC2 (Fig. 1c) separates ITA and SA river buffaloes. This structure was also confirmed (Supplementary Note 2, Supplementary Fig. 3) when whole-genome data were merged with samples genotyped using the 90 K Axiom^TM^ Buffalo Genotyping Array [14]. The PC3 (Fig. 1d, Supplementary Fig. 4, Supplementary Table 7) highlights the variability among swamp breeds, separating the southwest Chinese and Vietnamese buffaloes from other swamp breeds. The ADMIXTURE analysis confirmed this genetic structure (Fig. 1e, Supplementary Table 8, Supplementary Fig. 5). At *K* = 4, all individuals were unambiguously assigned to 2 ancestries in swamp buffalo (cold colors: SC, SEA) and 2 in river buffalo (warm colors: SA, ITA) (Fig. 1e). Within swamp buffalo, the SC ancestral component (blue) characterizes most of Upper and Middle-Lower Yangtze Valley buffaloes, but it was also detected in southwest Chinese and Laotian (LA) breeds. The SEA ancestry (green) was shared between Vietnamese and 3 southwest Chinese breeds, and found at a low level in Middle-Lower Yangtze, showing evidence of recent admixture, probably due to the frequent trading of buffaloes among these regions. As for the river buffalo, the SA ancestry (orange) was abundant in Murrah and Nili-Ravi buffaloes, while the ITA ancestry (red) is unique to Italian breeds. Some swamp buffaloes showed evidence of admixture, which may be attributable to introgression events by way of recent crossbreeding with river buffalo for improving milk production traits [16].

### Uniparental phylogenies

**Figure 2:**
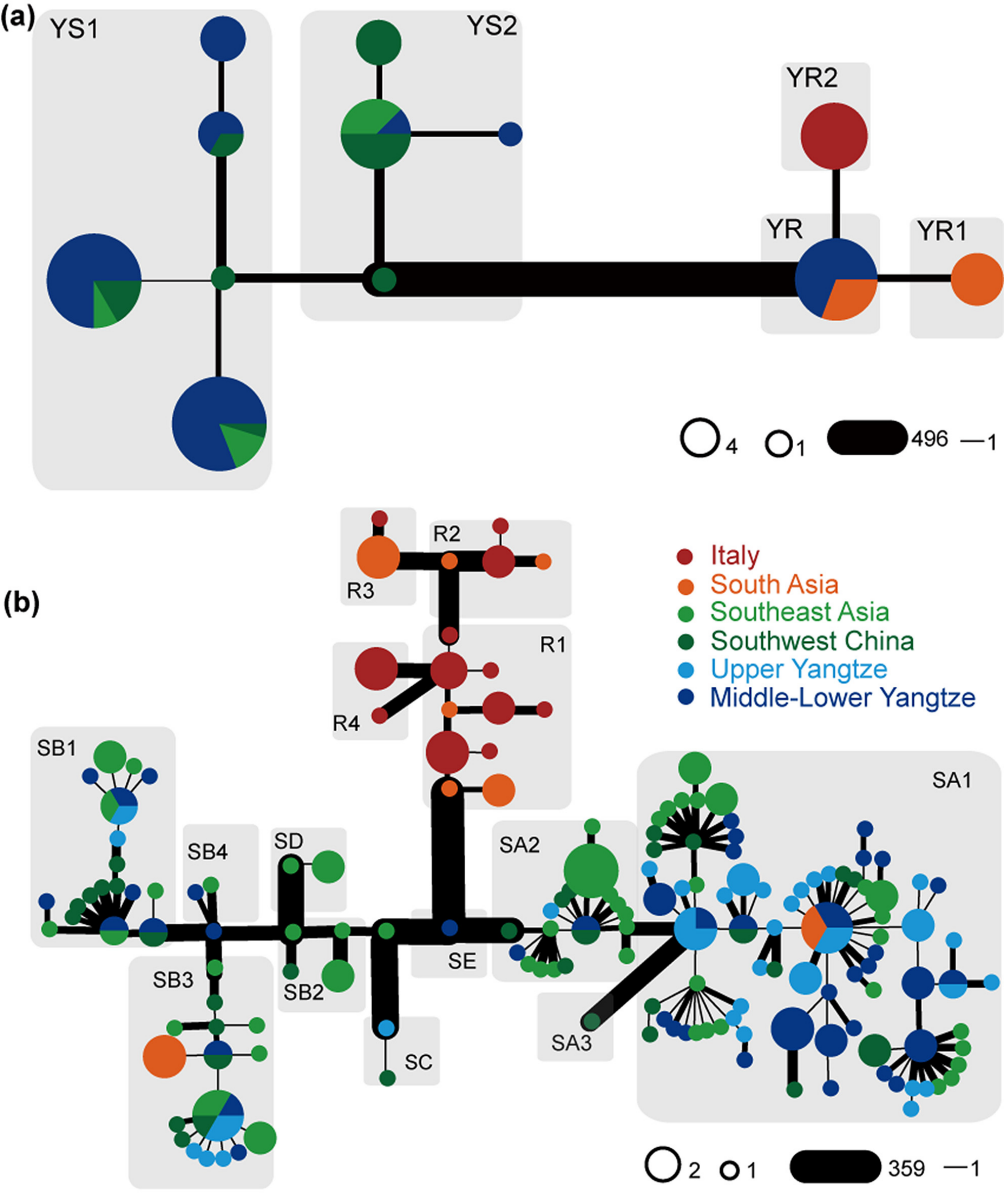
Y-chromosome and mitogenome phylogenies. The width of the edges is proportional to the number of pairwise differences between the joined haplotypes. (a) Y-chromosome network using 520 SNPs. (b) Mitogenome network of swamp buffalo. Different haplogroups of Y-chromosome and mitogenomes were labelled in each gray shadow.

Y-chromosome and mitochondrial DNA (mtDNA) are very useful to investigate genetic origins and ancient migrations (Supplementary Note 3). After quality control and filtering, 520 Y-chromosome single-nucleotide polymorphisms (SNPs) were retrieved from 89 male buffaloes and used to build a phylogenetic tree that clearly divides swamp (YS) and river (YR) clades. Most of the variants defined the branch that connects swamp and river common ancestors. Two haplogroups (YS1 and YS2) were identified in the swamp branch, both retrieved in all geographic regions. The haplogroup YS1 dominated the buffaloes from Upper and Middle-Lower Yangtze (76.09%), while the haplogroup YS2 was extremely frequent (84.62%) in southwest China and southeast Asia (Fig. 2a, Supplementary Fig. 6, and Supplementary Table 9).

We also inferred the maternal history of buffalo, combining the novel 118 mitogenomes from this study with 107 sequences from previous studies (Supplementary Table 10) [12]. Swamp buffaloes can be assigned to 5 previously defined lineages: 2 major haplogroups (SA haplogroup and SB haplogroup with various sub-clades) and 3 rare ones (SC, SD, and SE) (Fig. 2b, Supplementary Fig. 7, Supplementary Table 10). This larger dataset confirmed the geographic differentiation of current swamp buffalo populations (Fig.   1e), as previously reported by analyzing partial and complete mtDNA data [12, 13]. The Upper and Middle-Lower Yangtze buffalo breeds primarily belonged to lineage SA1. Buffaloes from southwest China and southeast Asia harbor almost all lineages, except for the rare lineage SE, and showed high frequencies of SA2 and SB2. The greatest variety of lineages was identified in southwest China and southeast Asia, thus confirming the hypothesized domestication of swamp buffalo at the border of the 2 regions [12, 14].

As for the Y-chromosome variation of river buffalo, we identified 1 ancestral node (YR) and 2 haplogroups (YR1 and YR2). The YR haplotype was found in 1 Indian, 2 south Asian, and 9 southern Chinese buffaloes. The latter finding was probably due to recent importation of bulls in China through cross-breeding programs [16], which is consistent with the autosomal analyses (PCA, ADMIXTURE, and NJ tree, Fig. 1b–e). The haplogroups YR1 and YR2 were found in south Asia and Italy, respectively (Fig. 2a and 2b and Supplementary Fig. 4). Four different mtDNA haplogroups have been identified in river buffaloes (Fig. 2b), thus adding a new lineage (R4) to the 3 previously defined ones (R1, R2, and R3). However, considering the low number (and coverage) of river mitogenomes (22 ITA and 10 SA buffaloes) no further phylogeographic analyses have been carried out (Fig. 2).

### Demographic history

**Figure 3:**
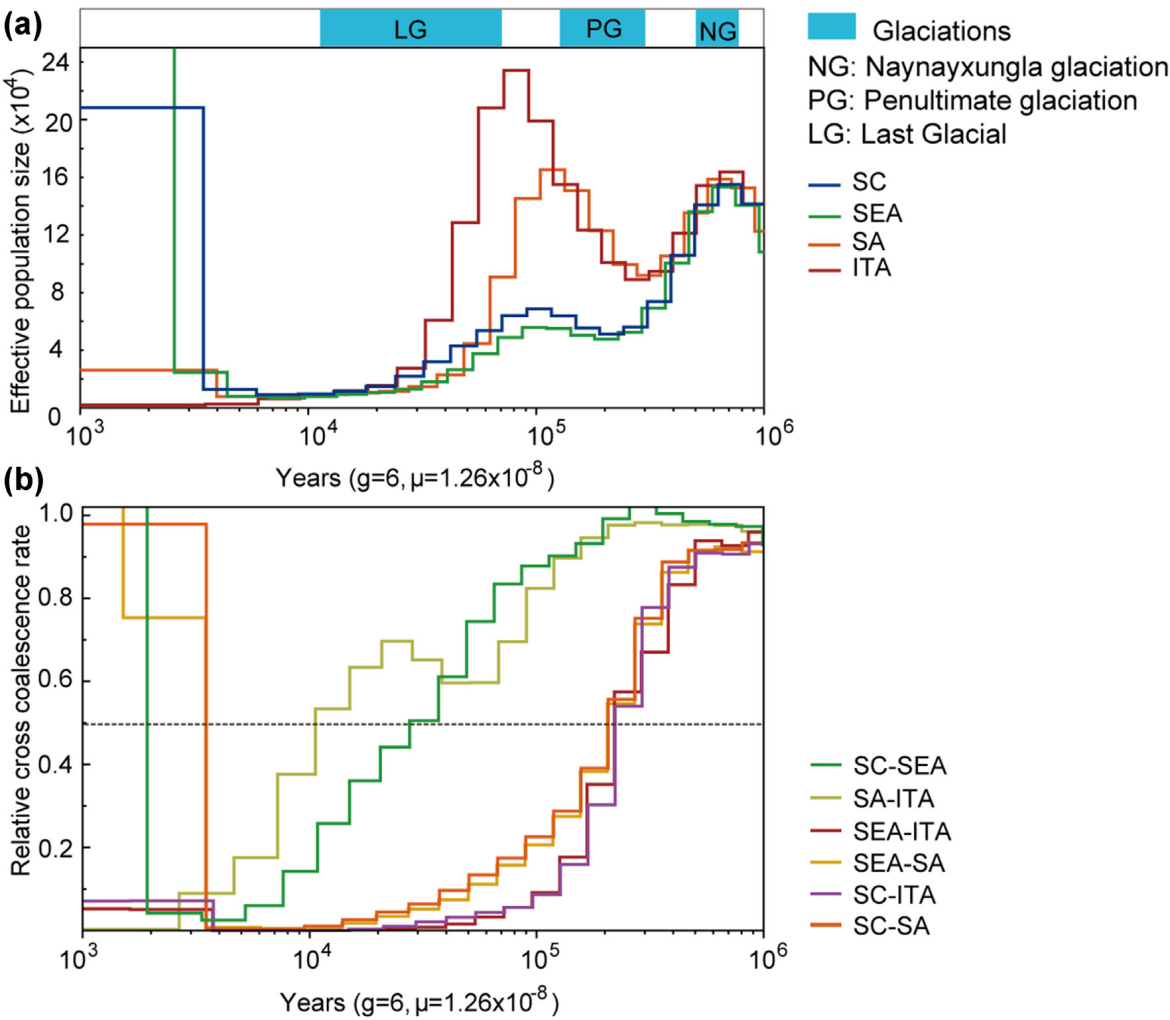
Demographic history and divergence of buffalo populations using MSMC. (a) Population size history inference of swamp and river buffalo based on 4 high-coverage haplotypes from southwest China (SC), southeast Asia (SEA), south Asia (SA), and Italy individuals (ITA). (b) Inferred relative cross-coalescence rates between pairs of populations over time based on the same 4 haplotypes.

We used the multiple sequentially Markovian coalescent (MSMC) method to detect the changes in the effective population size (*N_e_*) of 4 “ancestral” buffalo groups. River and swamp buffaloes underwent 2 apparent expansions and 2 bottlenecks that seemed to overlap with 3 major glacial cycles (Fig. 3a). Initially, the *N_e_* of river and swamp buffaloes showed similar demographic trajectories, peaking at ∼0.8 Mya and then quickly declining during the Naynayxungla glaciation (NG, 0.78–0.50 Mya), which was the most extensive glaciation during the Quaternary Period. The ancestral *N_e_* of river buffalo recovered very quickly and reached the highest peak at ∼70 kya after a short bottleneck ∼0.23 Mya. On the contrary, the ancestors of the swamp buffalo went through a long period of population decline until the retreat of the penultimate glaciation (PG, ∼0.30–0.13 Mya), and then, the *N_e_* slightly increased starting from ∼0.10 Mya. During the interglacial period, both river and swamp buffalo populations reached another peak and quickly declined during the last glaciation (LG). These results confirmed that the glaciations had a strong effect on the demographic history of swamp buffalo, as already observed by analyzing complete mtDNAs [12]. The decline from ∼6.0 to ∼4.5 kya is consistent with the onset of domestication, before the final increase until the present time.

The MSMC approach was also used to calculate the divergence time among 4 buffalo ancestral populations: SC, SEA, SA, and ITA (Fig. 3b). We observed a decrease in the cross-coalescence rate between river and swamp buffalo to 0.5 at ∼0.21–0.23 Mya (from 0.25 at ∼0.15–0.18 Mya to 0.75 at ∼0.28–0.38 Mya). The splitting time of SC and SA ancestors was observed at ∼28 kya, while a decline to 0.5 between ITA and SA was detected later at ∼11 kya.

### Genome-wide differential selection in river and swamp buffalo

We applied 4 methods (*F*_ST_, π ln ratio, cross-population composite likelihood ratio (XP-CLR), cross-population extended haplotype homozygosity (XP-EHH) to detect genomic regions related to selection in river and swamp buffalo. Two or more methods showed outlier signals (*P-*value < 0.005) in overlapping regions and were therefore considered as candidate selective regions. Functional gene set enrichment was used to identify KEGG pathways and Gene Ontology (GO) terms that are statistically significantly associated with the genes.

**Figure 4:**
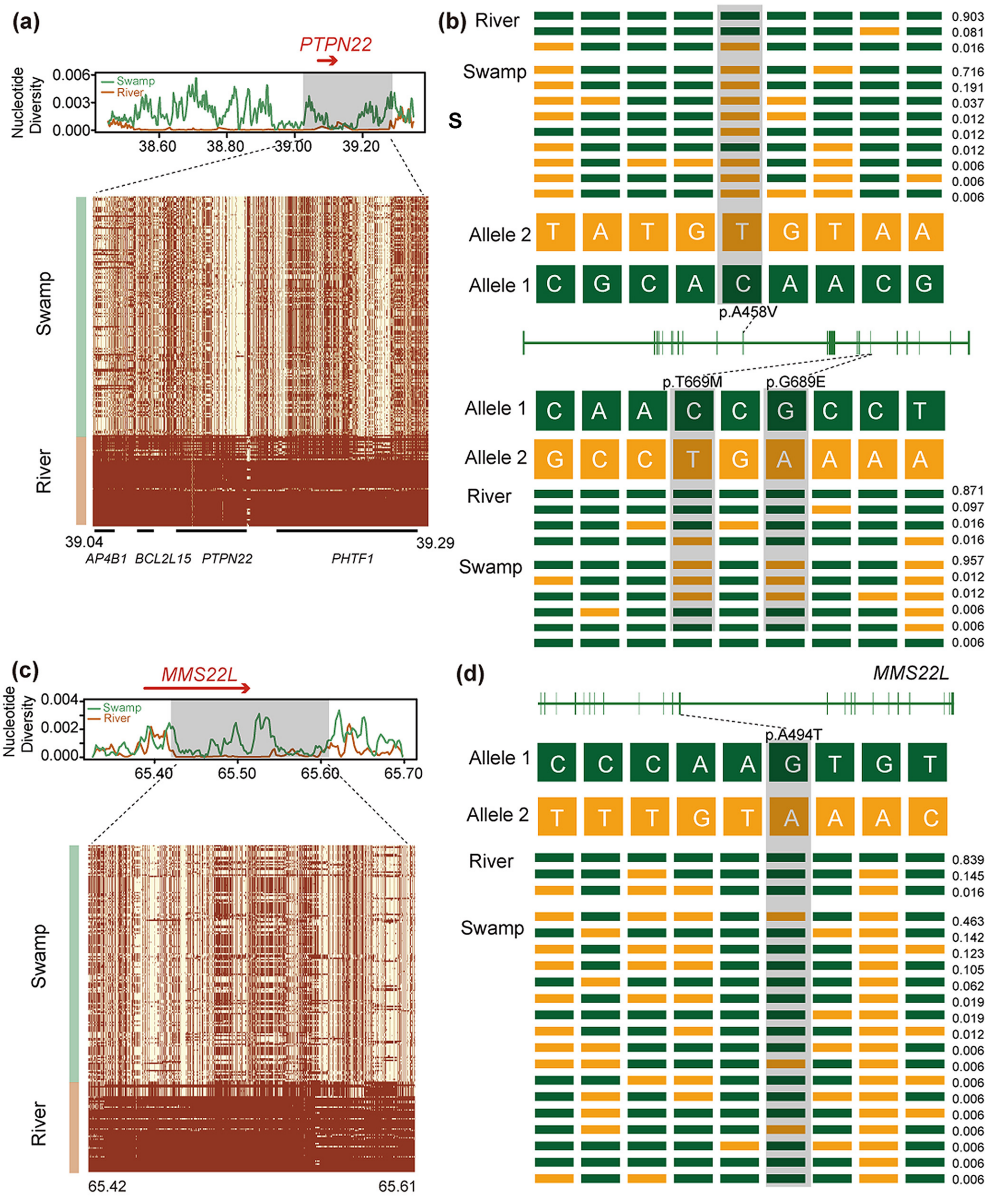
Signatures of selective sweep regions at *PTPN22* and *MMSL22* genes in river buffalo. Different parameters were estimated for each gene (*PTPN22* and *MMSL22*): nucleotide diversity, degree of haplotype sharing across populations (a and c). A red arrow notes the specific gene region. A schematic structure of each gene (b and d) is also depicted with exons indicated by vertical bars and reference/alternative alleles noted with different colors (green/yellow) and combined to form different haplotypes (each with a specific haplotype frequency next to it). Non-synonymous SNPs are highlighted in gray.

In river buffalo, a total of 502 candidate regions under selection containing 569 genes were detected (Supplementary Tables 11–14). Candidate genes in river buffalo are significantly over-represented (corrected *P-*value < 0.05) in the Jak-STAT signaling pathway, glioma, and pathways associated with cancer (Supplementary Tables 15 and 16). The Jak-STAT pathway plays a crucial role in prolactin signal transduction of the mammary gland [17] and control of immune responses [18, 19]. We also identified GO terms associated with immunity and others with DNA damage and repair (Supplementary Table 16, Supplementary Fig. 8a). In particular, 4 candidate genes (*AP4B1, BCL2L15, PHTF1*, and *PTPN22*) (Fig. a) are associated with the immune system response, while *MMS22L* (Fig. 4c) may be associated with heat stress. Moreover, we detected few non-synonymous variants that are completely fixed at *PTPN22* and *BCL2L15* in river buffaloes (Fig. 4b, Supplementary Fig. 8b), as well as 1 at *MMS22L* (Fig. 4d). Additional signs of selection were identified in genes coding for productive and economically significant traits, such as *NUMB* [20] and *SGMS2* [21] associated with milk production, while others related to growth (*NRF1*) [22] and feed efficiency (*TNPO3*) [23].

**Figure 5:**
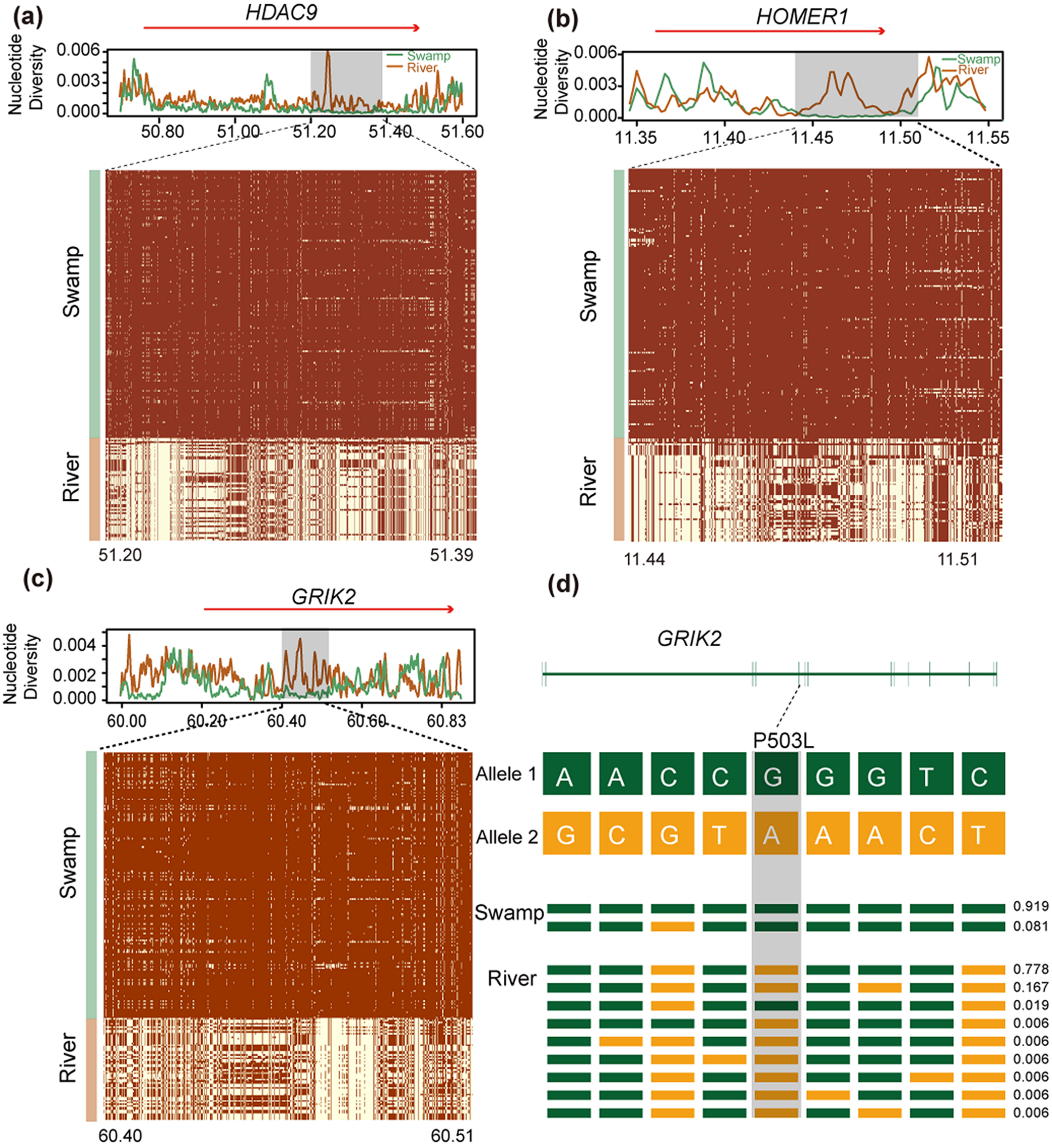
Signatures of selective sweep regions at *HDAC9, HOMER1*, and *GRIK2* genes in swamp buffalo. See the legend of Figure 4 for further details.

In swamp buffalo, a total of 171 candidate regions under selection containing 209 genes were detected (Supplementary Tables 11 and 17–19). These genes can be significantly associated (corrected *P-*value < 0.05) to 4 KEGG pathways (Supplementary Tables 20 and 21). The most significant one was “Glutamatergic synapse” involving 5 genes (*HOMER1, GRIK2, DLGAP1, GNG7, LOC102398542*) (Fig. 5, Supplementary Table 20, Supplementary Fig. 9). We also found significantly over-represented GO terms associated with dendritic spines (*TIAM1, RELN, DISC1, NLGN1, LOC102398542*) and nervous system (e.g., neuron, dendritic spine, synapse) involving 42 genes (*HDAC9, HOMER1, BIN1*, and *GRIK2* showed higher values in the detection methods) (Supplementary Table 21, Supplementary Fig. 9). Among these detected candidate genes, *HDAC9, HOMER1*, and *GRIK2* (Fig. F5a–c) may be associated with the development of the nervous system in swamp buffalo (Fig. 5d).

### Discussion

In this study, we analyzed the whole-genome sequence of 121 buffaloes (91 swamp and 30 river buffaloes). Our autosomal data reveal an ancient separation between river and swamp buffaloes ∼0.23 Mya, overlapping with previously reported data [9, 11, 12] and certainly predating buffalo domestication. Therefore, we can assume that river and swamp buffaloes probably descended from different wild populations. The demographic histories of swamp and river buffaloes were differentially linked to climatic changes. A similar pattern has been observed in taurine and indicine cattle, suggesting similar habitat requirements [24]. After diverging, the 2 types of buffalo evolved independently. In fact, 2 different ancestral components were identified for each of them (Fig. 1e). Distinctive Y-chromosome lineages were also revealed in river buffalo, with a basal haplogroup (YR) unique to breeds from India and Pakistan, YR1 typical of south Asia, and YR2 identified only in Italy. The most likely scenario based on previous studies [14] points to an early river buffalo domestication in the Indo-Pakistan region, followed by westward migration. Later, YR1 remained in south Asia, where it is still highly diffused, whereas YR2 became unique to the buffaloes bred in Italy. In swamp buffalo, YS2 is found mostly in southwest China and southeast Asia (84.62%), while YS1 dominates buffaloes from Upper and Middle-Lower Yangtze (76.09%). Considering that YS2 diverge earlier in the phylogeny, we might speculate that the swamp buffalo population migrated from the southern regions towards the north, where the YS1 experienced a population expansion. A clear geographic pattern was also identified in the mitochondrial gene pool of swamp buffalo. Taking into account the frequencies of uniparental haplogroups in swamp buffalo (Fig. 2), we could observe a correlation between Y-chromosome and mtDNA haplogroups (i.e., YS1 with SA and YS2 with SB), which could mark some similarities between maternal and paternal histories.

We identified significant and distinctive signatures of selective sweeps in these 2 types ow buffalo. To better understand possible explanations for the selective pressures, we have explored the most likely biological functions of these genes. River buffaloes are mainly distributed in western India to Mediterranean areas, where domestic herds are usually more resistant to various diseases than in tropical regions [16]. In river genomes, positively selected genes are significantly over-represented in GO terms associated with immunity. Among these, *PTPN22* encodes a negative regulator of T-cell receptor (TCR), which has been associated with human autoimmune diseases [25–28], bovine leukemia virus [29], and milk somatic cell counts of cow [29]. Among the other genes (*PTPN22, AP4B1, BCL2L15*, and *PHTF1*) were those related to bovine leukemia virus [30] and human autoimmune diseases [28]. The *BCL2L15* SNP A226G (acid changed: T76A) is a conserved region and almost fixed with the allele A in river buffalo (*P* > 0.90) (Supplementary Fig. 8b). In addition, heat stress is an important issue for many livestock species, particularly for dairy animals, leading to different problems in the phenotypic features (e.g., impairment of reproduction and slower growth), as well as in cellular stability, causing a reduced efficiency of DNA synthesis [31]. We found 3 GO terms associated with DNA damage and repair (cellular response to DNA damage stimulus; DNA repair; double-strand break repair) that are significantly over-represented in river buffalo. *MMS22L*, a component of the *MMS22L*-*TONSL* complex important for the DNA repair system [32, 33], was involved in these 3 GO terms. Actually, heat stress can induce the formation of double-stranded DNA breaks [34] and inhibit the functionality of the homologous recombination (HR) system [35]. Double-stranded DNA breaks can be repaired by HR, allowing DNA replication to continue at stalled or broken forks [36, 37]. *MMS22L* can facilitate HR-mediated maintenance of genome stability during DNA replication [38]. Taking into account that river buffalo is mainly selected for milk production and well adapted to a hot climate [39], these genes might play an important role in the heat adaptability of river buffalo. Finally, we were also able to identify signatures of selection on some genes for important economic and reproductive traits, which is not unexpected considering the great effort undertaken by the herders to improve the river breeds.

The swamp buffalo has historically been used as a draft animal to provide farm power in rice cultivation. Therefore, these animals are very docile and easy to handle and train. The swamp buffalo genome showed signs of selection in some genes of the “Glutamatergic synapse” pathway (*HOMER1, GRIK2, DLGAP1, GNG7*, and *LOC102398542*), which plays an important role in behavior, particularly concerning adaptation to stress and fear responses (Supplementary Table 20) [40]. We also found 42 genes that were over-represented in GO categories associated with the nervous system. Among them *HDAC9, HOMER1, BIN1*, and *GRIK2* seem the best candidates we have identified (Supplementary Table 21). *HDAC9* (Fig. 5a), a member of the class II HDAC proteins, plays a crucial role in neuronal differentiation during cortical development [41] and muscle development [42–45]. A study showed that degradation of class II HDAC proteins can activate myocyte enhancer factor 2, which enhances muscle endurance and fatigue resistance [46]. *HOMER1* (Fig. 5b) encodes a member of the homer family of dendritic proteins, involved in several psychiatric disorders, such as schizophrenia [47, 48] and major depression [49]. *HOMER1* plays an important role in brain development and behavior; *HOMER1* knockout mice showed deficits in learning and memorizing, and impairment of pain perception [47, 50–53]. *HOMER1* is also an important scaffold for TRP channels and regulates mechanotransduction in skeletal muscle [54]. Mice lacking *HOMER1* showed myopathy with decreased muscle fiber cross-sectional area and reduced skeletal muscle strength generation [54]. Studies have showed that *BIN1* is associated with Alzheimer disease [55–57]. *BIN1* is also involved in the biogenesis of T-tubules, which are responsible for the plasma membrane invaginations that allow for the excitation-contraction coupling machinery in cardiac and skeletal muscles [58–60]. *GRIK2* (Fig. 5c and 5d) encodes for GluR6, a kainite receptor that is highly expressed in the brain and is associated with autosomal recessive mental retardation [61]. *GRIK2* knockout mice exhibited reduction in fear memory [62], less anxiety, and more risk-taking type than despair-type behavior [63]. Notably, *GRIK2* was also identified as a candidate selective gene in domestic rabbits [64]. In addition, we also identified over-represented GO categories associated with dendritic spines (*TIAM1, RELN, DISC1, NLGN1, LOC102398542*) (Supplementary Fig. 9). The structural and functional plasticity of dendritic spines is crucial for learning and memorizing [65]. *TIAM1* plays an important role in the formation and morphogenesis of dendritic spines [66–68]. *RELN,DISC1*, and*NLGN1* were related to schizophrenia, mood disorders, and memory [69–77]. Therefore, these genes could be associated with nervous system development in swamp buffalo.

### Potential implications

This is the first population genetics study on buffalo using a large amount of whole-genome resequencing data. We reconstructed the genetic history and population structure of buffalo from all genetic perspectives using both uniparental and biparental markers. The final scenario indicates that the ancestors of swamp and river buffalo diverged ∼0.23 Mya. The swamp buffalo was then domesticated at the border between southwest China and southeast Asia, while the river buffalo was domesticated in south Asia (between northern India and Pakistan). The domestic herds then migrated to other regions and further differentiated. In fact, we were able to identify 2 ancestral and distinctive components in the current genomes of swamp (south China and southeast Asia components) and river (South Asia and Italy) populations. The different genetic history of these 2 subspecies is also evident by 2 distinct selection patterns identified in their genomes. River buffalo was selected to improve milk production, while the swamp buffalo was mainly raised to provide power for rice cultivation. We were able to intercept distinctive signature of selection in genes associated with nervous and muscle development in swamp buffaloes and in genes related to economic and reproductive traits in river breeds.

In summary, this is the first study providing a large amount of genomic data on river and swamp buffaloes, which was needed to describe their current genetic diversities and population structures, to reconstruct their demographic histories, and to scan for distinctive selective pressures.

## Methods

### Sample collection and sequencing

We sampled a total of 98 buffaloes from different locations: China (81), Laos (5), Vietnam (4), and India (1 Nili-Ravi, 2 Murrah, and 5 Indian buffaloes). Genomic DNA was extracted from ear tissue or blood samples using the standard phenol-chloroform protocol [78], amplified in genomic libraries with an average insert size of 500 bp, and sequenced (150-bp paired-end reads) on an Illumina HiSeq 2000. We also considered 23 available genome sequences from river buffalo, including 22 Mediterranean and 1 Murrah buffaloes. Additional details are provided in Supplementary Tables 1 and 2. This study was approved by Institutional Animal Care and Use Committee of Northwest A&F University (Permit No. NWAFAC1019).

### Alignments and variant identification

All cleaned reads were aligned to the reference genome (GCA_000471725.1) linked to “24+X+unplaced” pseudo-chromosomes (Supplementary Note 1, Supplementary Table 4) using BWA-MEM with default settings [79]. Duplicate reads were filtered using Picard tools. The SNPs were detected with GATK, version 3.6-0-g89b7209 [80], and filtered using the “VariantFiltration” tool, as described in Supplementary Note 1.

### Phylogenetic and population structure analyses

NJ tree, PCA, and ADMIXTURE methods were used to explore the genetic relationships among buffalo populations (Supplementary Note 2). An individual-based NJ tree based on the matrix of pairwise genetic distances from the autosomal SNP data of 121 buffaloes was constructed with PLINK version 1.9 (PLINK, RRID:SCR_001757) and visualized with FigTree (FigTree, RRID:SCR_008515). TreeMix software was used to construct a population-level phylogeny [81]. The PCA was performed using SmartPCA in the package EIGENSOFT v5.0 (Eigensoft, RRID:SCR_004965) [82] and the eigenvectors’ significance was detected by the Tracy-Widom test. The population genetic structure was estimated using ADMIXTURE v. 1.3.0 (ADMIXTURE, RRID:SCR_001263) [83] considering from 2 to 5 clusters (K).

### Y-chromosome and mitogenome phylogenies

After removing sites shared with female buffaloes, heterozygous sites, and sites with a genotyping rate <5%, a total of 520 male-specific SNPs were used to construct the phylogenetic tree with BEAST 1.8.0 (BEAST, RRID:SCR_010228) (Supplementary Note 3). A total of 98 mitochondrial genomes with an average coverage >100× were assembled from the whole-genome resequencing data. An additional 107 whole mtDNA sequences were obtained from GenBank. A phylogenetic tree based on the final alignment was constructed using RaxML (RAxML, RRID:SCR_006086) with the following parameters: -f a -x 123 -p 23 -# 100 -k -m 132 GTRGAMMA. The phylogenies were built using pegas [84].

### Estimates of the effective population size and divergence time

An MSMC was used to infer effective population sizes (*N_e_*) and divergence times considering 2 samples with average coverage >16× for each population. Autosomal SNPs of each sample were identified using GATK (GATK, RRID:SCR_001876). After removing variant outliers (with extremely low or high coverage), all sites were phased using BEAGLE v. 4.1 (BEAGLE, RRID:SCR_001789) [85]. The same high-coverage samples were also used to infer relative cross-coalescence rate, considering a value of 0.5 as a reference to extrapolate split times between populations (samples). The time scale is calculated using an average generation time of 6 years (g = 6) and a mutation rate of *μ*_g_ = 1.26 × 10^−8^ [86].

### Genome-wide selective sweep test

To detect selective sweeps in swamp and river buffalo, we performed the following comparisons: (i) the swamp buffalo as a reference and the river buffalo as the object population and (ii) the river buffalo as a reference and the swamp buffalo as the object population. A total of 4 methods were used. (i) The fixation index (*F*_ST_) values [87] were calculated in sliding 50-kb windows with 20-kb steps along the autosomes using VCFtools (VCFtools, RRID:SCR_001235) [88]. (ii) High differences in genetic diversity (π ln ratio) were calculated with 50-kb sliding windows and 20-kb steps along the autosomes using VCFtools and in-house scripts. (iii) The XP-CLR is a likelihood method that was used to detect whether the change in allele frequency at the locus likely occurred too quickly to be due to random drift between 2 populations [89]. We used such conditions to perform XP-CLR: non-overlapping sliding windows of 50 kb, maximum number of SNPs within each window of 600, and correlation level from which the SNP contribution to the XP-CLR result was down-weighted of 0.95. (iv) We also performed the XP-EHH test for every SNP using the default settings of selscan v1.1 [90], which was designed to detect ongoing or nearly fixed selective sweeps by comparing haplotypes from 2 populations [91]. For the XP-EHH selection scan, our test statistic was the average normalized XP-EHH score in each 50-kb region. Significant genomic regions were identified by *P-*value < 0.005. Two or more methods showed significant signals (*P-*value < 0.005) in overlapping regions and were therefore considered as the candidate regions affected by selection. The KOBAS 3.0 tool (KOBAS, RRID:SCR_006350) [92] was used to gain a better understanding of their biological functions and involved pathways as enriched GO terms and KEGG pathways.

## Availability of Supporting Data and Materials

Raw sequencing data are available from EBI European Nucleotide Archive Bioproject number PRJNA547460. All supporting genotype data and additional materials are available in the *GigaScience* GigaDB database [93].

## Additional Files

Supplementary Note 1. Linking pseudo-chromosomes.

Supplementary Note 2. The whole-genome diversity of buffalo.

Supplementary Note 3. Population structure analysis.

Supplementary Note 4. Y-chromosome and whole mitochondrial genome phylogeny.

Supplementary Figure 1. Genome-wide distribution of nucleotide diversity of buffaloes in 6 geographical regions in 50-kb sliding windows with 20-kb steps.

Supplementary Figure 2. TreeMix relationships between 25 buffalo breeds.

Supplementary Figure 3. PCA of river buffaloes, with PC1 plotted against PC2.

Supplementary Figure 4. PCA of swamp buffaloes, with PC1 plotted against PC2.

Supplementary Figure 5. Model-based clustering of buffalo using the ADMIXTURE program with K from 2 to 5.

Supplementary Figure 6. ML phylogeny of the Y-chromosome using 520 SNPs for 89 buffaloes.

Supplementary Figure 7. ML phylogeny of the mitochondrial genome.

Supplementary Figure 8. (a) Hierarchical graph of the over-represented (with significant corrected *P*-values < 0.05) GO terms associated with immunity. The color intensity is positively correlated to the corrected *P*-value of the GO term. (b) Amino acid conservation of *BCL2L15* (first exon) in mammals. Most amino acids are highly conserved with only a few exceptions, including SNP A226G (acid changed: T76A).

Supplementary Figure 9. (a) Hierarchical graph of the over-represented (with significant corrected *P*-values < 0.05) GO terms associated with the nervous system. The color intensity is positively correlated to the corrected *P*-values of the GO term. (b) Amino acid conservation of *LOC102398542*. Nonsynonymous SNP C455T (acid changed: P152L) located in the first exon. Amino acids at this site are highly conserved in other mammals.

Supplementary Table 1. Overview of sample information and sequencing statistics concerning the 121 buffaloes analyzed in this study.

Supplementary Table 2. Summary information on 25 buffalo breeds.

Supplementary Table 3. Distribution of SNPs within various genomic regions.

Supplementary Table 4. Summary information of the linked pseudo-chromosomes.

Supplementary Table 5. The θπ value for the buffalo population groups.

Supplementary Table 6. Pairwise *F*_ST_ values.

Supplementary Table 7. Tracy-Widom (TW) statistics and *P*-value for the 10 first eigenvalues in the PCA of buffaloes.

Supplementary Table 8. Cross-validation (CV) errors for ADMIXTURE ancestry models with K ranging from 2 to 5.

Supplementary Table 9. The genotype of 520 SNPs in the Y chromosome.

Supplementary Table 10. Mapping results for 118 novel buffalo mitochondrial genomes plus 107 published mitogenomes.

Supplementary Table 11. A summary of genes from *F*_ST_.

Supplementary Table 12. A summary of genes from XP-CLR (*P-*value < 0.5%) in river buffalo.

Supplementary Table 13. A summary of genes from ln ratio(π_swamp_/π_river_) (*P-*value < 0.5%) in river buffalo.

Supplementary Table 14. A summary of genes from XP-EHH in river buffalo.

Supplementary Table 15. KEGG pathway analysis of candidate genes in river buffalo.

Supplementary Table 16. GO enrichment of candidate genes in river buffalo.

Supplementary Table 17. A summary of genes from XP-CLR (*P-*value < 0.5%) in swamp buffalo.

Supplementary Table 18. A summary of genes from ln ratio(π_river_/π_swamp_) (*P-*value < 0.5%) in swamp buffalo.

Supplementary Table 19. A summary of genes from XP-EHH in swamp buffalo.

Supplementary Table 20. KEGG pathway analysis of candidate genes in swamp buffalo.

Supplementary Table 21. GO enrichment of candidate genes in swamp buffalo.

giz166_GIGA-D-19-00183_Original_Submission

giz166_GIGA-D-19-00183_Revision_1

giz166_Response_to_Reviewer_Comments_Original_Submission

giz166_Reviewer_1_Report_Original_Submission Madhu Tantia -- 7/30/2019 Reviewed

giz166_Reviewer_2_Report_Original_Submission Pierpaolo Maisano Delser -- 9/18/2019 Reviewed

giz166_Supplemental_Files

## Abbreviations

BWA: Burrows-Wheeler Aligner; bp: base pairs; GATK: Genome Analysis Toolkit; GO: Gene Ontology; HR: homologous recombination; ITA: Italy; kb: kilobase pairs; KEGG: Kyoto Encyclopedia of Genes and Genomes; kya: thousand years ago; LG: last glaciation; MSMC: multiple sequentially Markovian coalescent; mtDNA: mitochondrial DNA; Mya: million years ago; NG: Naynayxungla glaciation; NCBI: National Center for Biotechnology Information; NJ: neighbour-joining; PCA: principal component analysis; PG: penultimate glaciation; RAxML: Randomized Axelerated Maximum Likelihood; SA: south Asia; SC: southwest China; SEA: southeast Asia; SNP: single-nucleotide polymorphism; SRA: Sequence Read Archive; XP-CLR: cross-population composite likelihood ratio; XP-EHH: cross-population extended haplotype homozygosity.

## Competing Interests

The authors declare that they have no competing interests.

## Funding

The work was supported by the National Beef Cattle and Yak Industrial Technology System (CARS-37), Natural Science Foundation of China (31872317) to C.Z.L., and National Thousand Youth Talents Plan to Y.J.; and the Italian Ministry of Education, University and Research (MIUR), i.e., Dipartimenti di Eccellenza Program (2018–2022)-Dept. of Biology and Biotechnology “L. Spallanzani,” University of Pavia (to A.A.).

## Authors' Contributions

Y.J. and C.Z.L. conceived and supervised the experiments. T.S. and J.F.SH. performed the majority of the analysis with contributions from Q.M.CH., N.B.CH., and ZH.Q.ZH. T.S. and A.A. wrote and revised the manuscript. R.H.D., H.C.ZH, X.M.ZH, M.R.C., Y.ZH.H., X.Y.L., and H.CH. provided and prepared the samples. All authors reviewed the manuscript and gave final approval for publication.
