# Supplementary material for: Genomic analyses reveal distinct genetic architectures and selective pressures in buffaloes
Source: Gigascience. 2020 Feb 21;9(2):giz166. doi: 10.1093/gigascience/giz166 (PMC7033652; doi:10.1093/gigascience/giz166)
Supplement: giz166_GIGA-D-19-00183_Original_Submission [file giz166_giga-d-19-00183_original_submission.pdf]

GigaScience

Genomic Analyses Reveal Distinct Genetic Architectures and Selective Pressures in Buffaloes

--Manuscript Draft--

**Manuscript Number:**	GIGA-D-19-00183
**Full Title:**	Genomic Analyses Reveal Distinct Genetic Architectures and Selective Pressures in Buffaloes
**Article Type:**	Research
**Funding Information:**	National Beef Cattle and Yak Industrial Technology System (CARS-37) — Prof. Chuzhao Lei<br>Natural Science Foundation of China (31872317) — Prof. Chuzhao Lei<br>National Thousand Youth Talents Plan (none) — Prof. Yu Jiang<br>Dipartimenti di Eccellenza Program (none) — Not applicable
**Abstract:**	Background<br><br>The buffalo (Bubalus bubalis) is an essential farm animal with diverse genetic resources in tropical and subtropical regions, whose genomic value is yet to be discovered.<br><br>Results<br><br>In this study, we clarify the genetic, demographic and selective pressure of buffalo by analyzing 121 whole genomes (98 newly reported) from 25 swamp and river buffalo breeds. The ancestors of swamp and river buffalo diverged ~0.23 Mya and then experienced independent demographic history. Both uniparental and biparental markers were investigated to provide the final scenario. Briefly, they were domesticated in different regions, the swamp buffalo at the border between Southwest China and Southeast Asia, while the river buffalo in South Asia, then migrating to other regions and further differentiating, as testified by the (at least) two ancestral components identified in each subspecies. Moreover, distinct selective pressures were detected between these two types of buffalo. We were able to intercept distinctive signatures of selection in genes associated with nervous system in swamp buffalo, historically used as a draft animal, and in genes related to heat-stress, immunity in river dairy breeds.<br><br>Conclusions<br><br>Our findings substantially expand the catalogue of genetic variants in buffalo, and reveal new insights into the evolutionary history and distinct selective pressures in river and swamp buffalo.
**Corresponding Author:**	Yu Jiang, Ph.D<br>Northwest Agriculture and Forestry University<br>Yangling, Shaanxi CHINA
**Corresponding Author Secondary Information:**	
**Corresponding Author's Institution:**	Northwest Agriculture and Forestry University
**Corresponding Author's Secondary Institution:**	
**First Author:**	Ting Sun
**First Author Secondary Information:**	
**Order of Authors:**	Ting Sun

Powered by Editorial Manager® and ProduXion Manager® from Aries Systems Corporation

	Jiafei Shen
	Ningbo Chen
	Qiuming Chen
	Ruihua Dang
	Zhuqing Zheng
	Hucai Zhang
	Xiaoming Zhang
	Shaoqiang Wang
	Tao Zhang
	Hongzhao Lu
	Yun Ma
	Yutang Jia
	Marco Rosario Capodiferro
	Yongzhen Huang
	Xianyong Lan
	Hong Chen
	Alessandro Achilli
	Yu Jiang, Ph.D
	Chuzhao Lei
**Order of Authors Secondary Information:**	
**Additional Information:**	
**Question**	**Response**
Are you submitting this manuscript to a special series or article collection?	No
**Experimental design and statistics**<br><br>Full details of the experimental design and statistical methods used should be given in the Methods section, as detailed in our Minimum Standards Reporting Checklist. Information essential to interpreting the data presented should be made available in the figure legends.<br><br>Have you included all the information requested in your manuscript?	Yes
**Resources**<br><br>A description of all resources used, including antibodies, cell lines, animals	Yes

Powered by Editorial Manager® and ProduXion Manager® from Aries Systems Corporation

and software tools, with enough information to allow them to be uniquely identified, should be included in the Methods section. Authors are strongly encouraged to cite Research Resource Identifiers (RRIDs) for antibodies, model organisms and tools, where possible.<br><br>Have you included the information requested as detailed in our Minimum Standards Reporting Checklist?	
**Availability of data and materials**<br><br>All datasets and code on which the conclusions of the paper rely must be either included in your submission or deposited in publicly available repositories (where available and ethically appropriate), referencing such data using a unique identifier in the references and in the “Availability of Data and Materials” section of your manuscript.<br><br>Have you have met the above requirement as detailed in our Minimum Standards Reporting Checklist?	No
If not, please give reasons for any omissions below.<br><br>as follow-up to "**Availability of data and materials**<br><br>All datasets and code on which the conclusions of the paper rely must be either included in your submission or deposited in publicly available repositories (where available and ethically appropriate), referencing such data using a unique identifier in the references and in the “Availability of Data and Materials” section of your manuscript.<br><br>Have you have met the above	The 98 whole genomes resequecing data will be submit to the NCBI, and the accession number will be provided soon.

requirement as detailed in our Minimum Standards Reporting Checklist?<br><br>"	

Powered by Editorial Manager® and ProduXion Manager® from Aries Systems Corporation

Manuscript Click here to download Manuscript Main Document.docx

Genomic Analyses Reveal Distinct Genetic Architectures and Selective Pressures in Buffaloes

Ting Sun[1#], Jiafei Shen [1#], Ningbo Chen[1#], Qiuming Chen[1#], Ruihua Dang[1#], Zhuqing Zheng[1], Hucai Zhang[2], Xiaoming Zhang[3], Shaoqiang Wang[1], Tao Zhang[4], Hongzhao Lu[4], Yun Ma[5], Yutang Jia[6], Marco Rosario Capodiferro[7], Yongzhen Huang[1], Xianyong Lan[1], Hong Chen[1], Alessandro Achilli[7*], Yu Jiang[1*], Chuzhao Lei[1*]

[1]Key Laboratory of Animal Genetics, Breeding and Reproduction of Shaanxi Province, College of Animal Science and Technology, Northwest A&F University, Yangling, Shaanxi 712100, China.

[2]Key Laboratory of Plateau Lake Ecology and Environment Change, Yunnan University, Kunming 650504, China.

[3]State Key Laboratory of Genetic Resources and Evolution, Kunming Institute of Zoology, Chinese Academy of Sciences, Kunming 650223, China.

[4]School of Bioscience and Engineering, Shaanxi University of Technology, Hanzhong, Shaanxi 723000, China.

[5]Agricultural College, Ningxia University, Yinchuan 750021, China

[6]Institute of Animal Science and Veterinary Medicine, Anhui Academy of Agriculture Science, Hefei 230001, China.

[7]Dipartimento di Biologia e Biotecnologie "L. Spallanzani", Università di Pavia, Pavia, 27100, Italy.

e-mail addresses:

Ting Sun: sunting_sim07@163.com

Jiafei Shen: shenjiafei0118@163.com

Ningbo Chen: ningboch@126.com

Qiuming Chen: cqm19860612@126.com

Ruihua Dang: dangruihua@nwsuaf.edu.cn

Zhuqing Zheng: zzq1207@126.com

Hucai Zhang: hucaizhang@yahoo.com

Xiaoming Zhang: zhangxiaoming@mail.kiz.ac.cn

Shaoqiang Wang: oracle_2@163.com

Tao Zhang: zl780823@163.com

Hongzhao Lu:zl780823@126.com

Yun Ma: mayun666@yahoo.com.cn

Yutang Jia: yutang2018@163.com

Marco Rosario Capodiferro: marcorosario.capodiferro01@universitadipavia.it

Yongzhen Huang: huangyongzhen126@126.com

Xianyong Lan: lanxianyong79@nwafu.edu.cn

Hong Chen: chenhong1212@126.com

Alessandro Achilli: alessandro.achilli@unipv.it

Yu Jiang: yu.jiang@nwafu.edu.cn

Chuzhao Lei: leichuzhao1118@nwafu.edu.cn

*These authors contributed equally to this work.

*Corresponding author. E-mail: yu.jiang@nwafu.edu.cn (Y.J.) and leichuzhao1118@126.com (C.L.).

Abstract

Background

The buffalo (*Bubalus bubalis*) is an essential farm animal with diverse genetic resources in tropical and subtropical regions, whose genomic value is yet to be discovered.

Results

In this study, we clarify the genetic, demographic and selective pressure of buffalo by analyzing 121 whole genomes (98 newly reported) from 25 swamp and river buffalo breeds. The ancestors of swamp and river buffalo diverged ~0.23 Mya and then experienced independent demographic history. Both uniparental and biparental markers were investigated to provide the final scenario. Briefly, they were domesticated in different regions, the swamp buffalo at the border between Southwest China and Southeast Asia, while the river buffalo in South Asia, then migrating to other regions and further differentiating, as testified by the (at least) two ancestral components identified in each subspecies. Moreover, distinct selective pressures were detected between these two types of buffalo. We were able to intercept distinctive signatures of selection in genes associated with nervous system in swamp buffalo, historically used as a draft animal, and in genes related to heat-stress, immunity in river dairy breeds.

Conclusions

Our findings substantially expand the catalogue of genetic variants in buffalo, and reveal new insights into the evolutionary history and distinct selective pressures in river and swamp buffalo.

Key words: buffalo; whole-genome resequencing; history; selection

Background

The domestic buffalo, an important farm animal in tropical and subtropical regions, which can provide milk, meat, and draught power for the rice cultivation. The domestic buffalo can be divided into two types: the swamp and river buffalo. The two types showing different body size, outward appearance, biological characteristics and chromosome karyotype (swamp buffalo: 2n = 48; river buffalo: 2n = 50) [1, 2]. The swamp buffalo is mainly bred in extensive rural areas in Northeast India, Southeast Asia and South China, while the river buffalo is distributed from Western India to Mediterranean areas. Swamp buffalo was traditionally raised as a draught animal for rice cultivation; while the river type was mainly selected for milk production [3].

There is a large agreement on the common ancestor of river and swamp buffaloes, both descended from the wild Asian buffalo (*Bubalus arnee*) [3]. However, the details of the domestication process and its consequences are still missing. The oldest domestic buffalo remains of Southeast Asia were found in Northern Thailand and dated to 2,900-2,300 years before present (YBP) [4], while other archeozoological evidence is scarce [5]. Therefore, the genomic screening of current breeds was often employed to clarify the overall scenario. In particular, both uniparental markers were initially screened [6-13], while further details were eventually provided by autosomal analyses [14]. The current data on river buffalo point to an initial domestication in the Indian subcontinent 6,300-4,600 YBP and a following migration westwards into Southern Europe [6, 10, 14]. The swamp buffalo maybe domesticated in the China/Indochina border and then differentially migrated to other regions: northward to China and then bending southwards into the Philippines; southward initially across the Mekong, then to Sumatra (Mekong colonization) and finally eastwards to the remainder of Indonesia [12-14].

However, considering that the entire genome variation of buffalo was largely unexplored [15], we sequenced the whole genome of 98 buffaloes from 21 swamp and four river breeds (Supplementary tables 1 and 2) with different geographic origins in order to fully describe the genomic diversity, population structure, and demographic history of this important livestock species and to reveal possible signs of natural and artificial selection.

Data Description

Whole-genome sequencing of 98 geographically diverse (from China, Laos, and Vietnam) modern buffaloes generated a total of 26.59 billion paired-end reads giving an average depth (coverage) of 9.6× and a mean mapping rate of 98.89%. To place these buffaloes in a more widespread phylogeographic context, we combined our data with 23 available genomes (figure 1a, Supplementary tables 1 and 2) [15]. The final dataset of 121 genomes was subdivided into six geographic groups: Upper Yangtze, Middle-Lower Yangtze, Southwest China, Southeast Asia, South Asia and Italy (figure 1a).

In total, around 34.4 million putative autosomal single nucleotide variants (SNPs) were identified (3,427,636 with a minor allele frequency <1%, 2,643,818 between 1% and 5%, and 28,281,439 > 5%), including 69,396 nonsynonymous and 153,317 synonymous coding single nucleotide polymorphisms. In particular, 25,632,159 were retrieved from swamp and 21,454,466 from river buffaloes (Supplementary table 4). As for the swamp buffaloes (except for the samples exhibiting admixture with river buffalo), populations from Southwest China and Southeast Asia showed a higher genomic diversity (1.74×10^{-3} and 1.66×10^{-3}, respectively) than those from Upper (1.61×10^{-3}) and Middle-lower Yangtze (1.65×10^{-3}) (Supplementary figure 1, Supplementary table 5). The genomic diversity of river buffalo was much higher in South Asia (2.21×10^{-3}) than in the Mediterranean area (1.59×10^{-3}). The population-differentiation statistics (F_{ST}) revealed a deep division between swamp and river buffaloes, but lower genetic differentiation among populations from closer geographical regions (Supplementary table 6).

Analysis

Population genetic structure and relationship

Neighbour-joining (NJ) trees, principal component analysis (PCA), and ADMIXTURE were used to explore the genetic relationships among the examined buffalo populations. The NJ tree showed a deep division of the swamp and river buffalo. Moreover, buffaloes from adjacent geographical regions formed distinctive clades (figure 1b). The same geographic/genomic proximity was also confirmed by the maximum-likelihood (ML) tree (Supplementary figure 2).

The PCA (figure 1c and 1d) showed that the first principal component (PC1) was driven by differences between swamp and river buffaloes explaining 11.73% of the total genetic variance. The PC2 (figure 1c) separated the Italian river buffaloes from South Asian ones. The separation between Italian and South Asian buffaloes is also confirmed (Supplementary Note2, Supplementary figure 3) when whole genome samples are merged with samples genotyped using the 90K Axiom^{TM} Buffalo Genotyping Array [14]. The PC3 (figure 1d, Supplementary figure 4, Supplementary table 7) highlights the variability among swamp breeds separating the Southwest Chinese and Vietnamese buffaloes from other swamp breeds. The ADMIXTURE analysis also confirmed these genetic separations (figure 1e, Supplementary table 8, Supplementary figure 5). At $K = 4$, all individuals were unambiguously assigned into two ancestries in swamp buffalo (cold colors: South China, SC; Southeast Asia, SEA) and two in river buffalo (warm colors: South Asia, SA; Italy, ITA) (figure 1e). Within swamp buffalo, the SC ancestral component (blue) characterizes most of Upper and Middle-Lower Yangtze Valley buffaloes, but it is also detected in Southwest Chinese and Laotian (LA) breeds. The SEA ancestry (green) is shared between Vietnamese and three Southwest Chinese breeds, and found at low level in Middle-Lower Yangtze showing evidence of recent admixture, which may be due to the frequent trading of buffaloes among these regions. As for the river buffalo, the SA ancestry (orange) is abundant in Murrah and Nili-Ravi buffaloes, while the ITA ancestry (red) is unique to Italian breeds. Some swamp buffaloes showed evidence of admixture, which may be attributable to introgression events by way of recent crossbreeding with river buffalo for improving the milk production traits [16].

Uniparental phylogenies

Y-chromosome and mitochondrial DNA (mtDNA) are very useful to investigate genetic origins and ancient migrations (Supplementary Note 3). After quality control and filtering, 520 Y-chromosome SNPs were retrieved from 89 male buffaloes and used to build a phylogenetic tree that clearly divides swamp (YS) and river (YR) clades. Most variants defined the branch that connects swamp and river common ancestors. Two haplogroups (YS1 and YS2) were identified

in the swamp branch, both retrieved in all geographic regions. The haplogroups YS1 dominated the buffaloes from Upper and Middle-Lower Yangtze (76.09%), while the haplogroups YS2 was extremely frequent (84.62%) in Southwest China and Southeast Asia (figure 2a, Supplementary figure 6, and Supplementary table 9). We also inferred the maternal lineages of swamp buffalo combining the novel 91 mitogenomes (coverage > 100 ×) from this study with 107 sequences from previous studies (Supplementary table 10) [12]. Swamp buffaloes can be assigned into the five previously defined lineages: two major haplogroups (SA and SB with various sub-clades) and three rare ones (SC, SD, and SE) (figure 2b, Supplementary figure 7, Supplementary table 10). This larger dataset confirmed the geographic differentiation of current swamp buffalo populations (figure 1e), as previously reported by analyzing partial and complete mtDNA data [12, 13]. The Upper and Middle-Lower Yangtze buffalo breeds primarily belong to lineage SA1. The Southwest China and Southeast Asia buffaloes almost harbor all lineages except for the rare lineage SE, and also showing a high frequency in lineage SA2 and SB2. The highest variety of lineages was identified in Southwest China and Southeast Asia, thus confirming the hypothesized maternal origin of swamp buffalo at the border of the two regions [12, 14].

In river buffalo, we only analyzed the Y-chromosome variation due to the low coverage of mitogenome. We identified one ancestral node (YR) and two haplogroups (YR1 and YR2). The YR haplotype was found in one Indian, two south Asian and nine Southern Chinese buffaloes. The latter finding was probably due to recent importation of bulls in China through the cross-breeding programs [16], consistently with the autosomal analyses (PCA, ADMIXTURE and NJ tree, figure 1b-1e). The haplogroups YR1 and YR2 were found in South Asia and Italy, respectively (figure 2a, 2b, and Supplementary figure 4).

Demographic history

We employed the multiple sequentially Markovian coalescent (MSMC) method to detect the changes in the effective population size (N_e) of four "ancestry" buffalo groups. We applied this method to all groups with two deep-coverage (> 16×) individuals per group.

Both the river and swamp buffaloes underwent two apparent expansions and two bottlenecks that mirrored the glacial cycles (figure 3a). Initially, the ancestral N_e of both river and swamp buffaloes showed similar demographic trajectories with a peak at ~0.8 Mya and then quickly declined during the Naynayxungla glaciation (NG, 0.78-0.50 Mya) which was the most extensive glaciation during the Quaternary Period. The ancestral N_e of river buffalo recovered very quickly and reached the highest peak at ~70 kya after a short bottleneck ~0.23 Mya. On the contrary, the ancestors of the swamp buffalo suffered a long period of population decline until the retreat of the Penultimate glaciation (PG, ~0.30-0.13 Mya), and then, the N_e slightly increased starting from ~0.10 Mya. During the interglacial period, both river and swamp buffalo population reached another peak and then quickly declined during the last glaciation (LG). These results confirmed that the glaciations had a strong effect on the demographic history of swamp buffalo, as already observed analyzing complete mitochondrial DNA [12]. The decline from ~6.0 to ~4.5 kya is consistent with the onset of domestication, before the final increase until present time.

The MSMC approach was also used to calculate the divergence time among four buffalo ancestry population: SC, SEA, SA and ITA (figure 3b). We observed a decrease in the cross-coalescence rate between river and swamp buffaloes to 0.5 at approximately 0.21~0.23 Mya (0.25 at approximately at 0.15 ~ 0.18 Mya; to 0.75 at approximately 0.28~0.38 Mya). The splitting time of SC and SA ancestors was observed at ~28 kya, while a decline to 0.5 between ITA and SA was detected later, at ~11 kya.

Genome-wide differential selection in river and swamp buffalo

We applied the four methods (F_{ST}, π ln ratio, XP-CLR, XP-EHH) to detect genomic regions related to selection in river and swamp buffalo. Two or more methods showed outlier signals (*P*-value < 0.005) in overlapping regions and were therefore considered as candidate selective regions. Then, we performed Kyoto Encyclopedia of Genes and Genomes (KEGG) and Gene Ontology (GO) enrichment analysis of candidate genes to provide distinctive results for each subspecies.

In river buffalo, a total of 502 candidate selective region containing 569 genes were detected (Supplementary table 11-14). Candidate selected genes in river buffalo were significantly over-represented (Corrected *P*-value < 0.05) in Jak-STAT signaling pathway, Glioma, and pathways associated with cancer (Supplementary table 15-16). The Jak-Stat pathway plays a crucial role in prolactin signal transduction of mammary gland [17] and control of immune responses [18, 19]. We also identified GO terms associated with immunity, DNA damage and repair (Supplementary table 16, Supplementary figure 8a). Among these candidate genes, four genes (*AP4B1*, *BCL2L15*, *PHTF1*, and *PTPN22*) are involved in the immune system response, and *MMS22L* may be associated with heat stress. These regions show lower nucleotide diversity and high level of haplotype homozygosity in river buffalo (figure 4a, 4c). In particular, we detected several non-synonymous variants that are completely fixed at *PTPN22*, *BCL2L15* in river buffaloes (figure 4b, Supplementary figure 8b), as well as one at *MMS22L* (figure 4d). We also detected some genes under selection that are associated with productive, or economically significant traits of the river buffalo, such as *NUMB* [20] and *SGMS2* [21] associated with milk production, while others related to growth (*NRF1*) [22], feed efficiency (*TNPO3*) [23].

In swamp buffalo, a total of 171 candidate selective region containing 209 genes were detected (Supplementary table 11, Supplementary tables 17-19). Four KEGG pathways (Glutamatergic synapse, Cytokine-cytokine receptor interaction; Glyoxylate and dicarboxylate metabolism, Homologous recombination) were significant over-represented (Corrected *P*-Value < 0.05) for swamp buffalo (Supplementary tables 20-21). The most significantly over-represented pathway was "Glutamatergic synapse" involving five genes (*HOMER1*, *GRIK2*, *DLGAP1*,*GNG7*, *LOC102398542*), which plays an important role in the behavioral adaptation of stress and fear responses [24]. We also found significantly over-represented GO categories associated with nervous system (neuron, dendrite, synapse, et al.) (Supplementary table 21, Supplementary figure 9). All these selected regions show lower nucleotide diversity and almost pure haplotype homozygosity in swamp buffalo (figure 5).

Discussion

In this study, we analyzed the whole genome sequence of 121 buffaloes (91 swamp and 30 river buffaloes). Our autosomal data revealed an ancient separation between river and swamp buffaloes ~0.23 Mya ago predating buffalo domestication, indicating that river and swamp buffalo might be descendants from divergent wild populations. This divergence time overlapped with the molecular divergence of swamp and river buffaloes (~10 Kya to ~0.9 Mya) [9, 11, 12]. The demographic histories of swamp and river buffaloes were differentially linked to climatic changes, and a similar pattern was observed in taurus and indicine, which probably suggested the similar habitat requirements [25]. After divergence, the two types of buffalo evolved independently. We are able to identify two different ancestral components for each of them (figure 1). Distinctive lineages were also revealed by the Y-chromosome analysis of river buffalo, with a basal haplogroup (YR) unique to breeds from India and Pakistan, YR1 typical of South Asia and YR2 identified only in Italy. The most likely scenario based on previous studies [14] points to an early river buffalo domestication in the Indo-Pakistan region, and then migrated westward. Later, YR1 remained in South Asia, where is still highly diffused, whereas YR2 became unique to the buffaloes bred in Italy. In swamp buffalo, YS2 is found mostly (84.62%) in in Southwest China and Southeast Asia, while YS1 dominates buffaloes from Upper and Middle-Lower Yangtze (76.09%). Considering that YS2 diverge earlier in the phylogeny, we might speculate that swamp buffalo population migrated from the southern regions towards the north, where the YS1 experienced a population expansion. A clear structure was also identified in the mitochondrial gene pool of swamp buffalo. Taking into account the frequencies of uniparental haplogroups in swamp buffalo (figure 2), we could observe a correlation between Y-chromosome and mtDNA haplogroups (i.e YS1 with SA and YS2 with SB) which could mark some similarities between maternal and paternal histories.

We identified significant distinct signatures of selective sweeps in these two types buffalo. River buffaloes are mainly distributed in Western India to Mediterranean areas, which are more disease resistance despite of tropical environment [16]. In river buffalo, candidate selected genes are significantly over-represented in GO terms associated with immunity. Among these,

PTPN22 encodes a negative regulator of T-cell receptor (TCR), which was associated with human autoimmune diseases [26-29], bovine leukemia virus [30], and milk somatic cell counts of cow [30]. Several studies have proved that *PTPN22*, *AP4B1*, *BCL2L15*, *PHTF1* may be associated with bovine leukemia virus [31], and other human autoimmune diseases [29]. *BCL2L15* gene A226G (acid changed: T76A) locus of river buffalo is almost fixed with the A allele (P > 0.90), and the G allele is dominant in swamp buffalo. This site is conservation in many mammals except for the allele present in river buffalo (Supplementary figure 8b). The selective sweep region including these four genes showed lower nucleotide diversity and almost pure haplotype homozygosity in river buffalo. So, we speculate that *PTPN22, AP4B1, BCL2L15, PHTF1* may be associated with the immune response for river buffalo. In addition, heat stress is a significant issue for many livestock, particularly for dairy animal, which can result in impairment of reproduction and slower growth. River buffalo is mainly selected for milk production and well adapted to hot climate [32]. There are three significant over-represented GO terms associated with DNA damage and repair (cellular response to DNA damage stimulus, GO:0006974; DNA repair, GO:0006281; double-strand break repair, GO:0006302) which are significantly over-represented in river buffalo. DNA synthesis remains inhibited during a longer period after the heat exposure, and heat stress will induce DNA polymerization brokendown [33]. *MMS22L*, a component of the *MMS22L-TONSL* complex, is involved in these three GO terms, which is important for the DNA repair system [34, 35]. Actually, heat stress can induce the formation of double-stranded DNA break (DSB) [36] and inhibit the functioning of the homologous recombination system [37]. Whereas, DSB can be repaired by homologous recombination, allowing DNA replication to continue at stalled or broken forks [38, 39]. *MMS22L* can facilitate HR-mediated maintenance of genome stability during DNA replication [40]. *MMS22L* shows a local reduction in nucleotide diversity and almost pure haplotype homozygosity in river buffalo, which may be act as a candidate gene associated with the heat adaption in river buffalo. These results suggest that these three genes might play an important role in the heat adaptability of river buffalo. Finally, we were able to identify signatures of selection also on some genes for important economic and reproductive traits, which is expected

considering the great effort undertaken by the herders to improve their breeds.

Swamp buffalo was historically used as a draft animal to provide farm power in rice cultivation, which is very docile and easy to handle and train. The "Glutamatergic synapse" pathway (*HOMER1*, *GRIK2*, *DLGAP1*, *GNG7*, *LOC102398542*) was most significantly over-represented in swamp buffalo (Supplementary table 20), which plays an important role in the behavioral adaptation of stress and fear responses [24]. We also found significantly over-represented GO categories associated with nervous system (neuron, dendritic spine, synapse, et al) involving 42 genes (Supplementary table 21). In addition, there are several over-represented GO categories involved in the dendritic spines (*TIAM1*, *RELN*, *DISC1*, *NLGN1*, *LOC102398542*) (Supplementary figure 9a). The structural and functional plasticity of dendritic spines is the cellular basis of learning and memory [41]. *TIAM1* plays an important role in the formation and morphogenesis of dendritic spines [42-44]. *RELN* is involved in the development of cerebrums, and related to schizophrenia [45-47]. *DISC1* also has been reported to be associated with schizophrenia and mood disorders [48-50]. *NLGN1* plays an important role in memory consolidation and strengthening [51]. *LOC102398542*, whose homologous gene is *DLG4* in human, plays a key role in memory, and is also related to schizophrenia [52, 53]. Further analysis showed that *LOC102398542* (acid changed: P152L) locus of swamp buffalo is almost fixed with the T allele, and the C allele is dominant in river buffalo. This site is conservation in other mammals except for swamp buffalo (Supplementary figure 9b). Among the 42 candidate genes involving in the nervous system, *HDAC9*, *HOMER1*, *BIN1*, *GRIK2* showed higher values. Due to the pleiotropic effects of genes, selection may possibly act on other biological function of the genes than those highlighted here. *HDAC9*, a member of class II HDAC proteins, plays a crucial role in neuronal differentiation during cortical development [54] and muscle development [55-58]. A study showed that degradation of class II HDAC proteins can activate myocyte enhancer factor 2, which enhances muscle endurance and fatigue resistance [59]. *HOMER1*, encodes a member of the homer family of dendritic proteins, involving in the several psychiatric disorders, such as schizophrenia [60, 61], major depression [62]. *HOMER1* plays an important role in brain development and behavior, and the *HOMER1*

knockout mice showed deficits of learning and memory, and impairment of pain perception [60, 63-66]. *HOMER1* is also an important scaffold for TRP channels and regulates mechanotransduction in skeletal muscle [67]. Mice lacking *HOMER1* showed myopathy with decreased muscle fiber cross-sectional area and reduced skeletal muscle strength generation [67]. The candidate regions with the two genes showed lower nucleotide diversity and almost pure haplotype homozygosity in swamp buffalo (figure 5). Studies have showed that *BIN1* is associated with Alzheimer Disease [68-70]. The *BIN1* is also involved in the biogenesis of T-tubules, which are responsible for the plasma membrane invaginations that allow for the excitation-contraction coupling machinery in cardiac and skeletal muscles [71-73]. *GRIK2*, encodes for GluR6, a kainite receptor which is highly expressed in the brain and is associated with autosomal recessive mental retardation [74]. The *GRIK2* knockout mice exhibited the reduction in fear memory [75], less anxious or more risk-taking type behavior and less despair-type manifestations [76]. Notably, *GRIK2* was also identified as a candidate selective gene in domestic rabbits [77]. *HDAC9*, *HOMER1*, *GRIK2* show local reduction in nucleotide diversity and almost pure haplotype homozygosity in swamp buffalo, which may be act as candidate genes associated with the development of nervous system in swamp buffalo.

Potential implications

This is the first population genetics study on buffalo using a large amount of whole-genome resequencing data. We reconstructed the genetic history and population structure of buffalo from all genetic perspectives using both uniparental and biparental markers. The final scenario indicates that the ancestors of swamp and river buffalo diverged about 0.23 Mya. The swamp at the border between Southwest China to Southeast Asia, while the river in South Asia (between Northern India and Pakistan); then migrated to other regions and further differentiated. In fact, we were able to identify two ancestral and distinctive components in the current genomes of both swamp (South China and Southeast Asia components) and river (South Asia and Italy) buffalo populations. River buffalo was selected to improve milk production, while the swamp buffalo was mainly raised to provide power for the rice cultivation. Our result

showed that river and swamp buffalo experienced distinct selective pressures. We were able to intercept the distinctive marks by identifying distinctive signature of selection in genes associated with nervous and muscle development in swamp buffaloes and in genes related to economic and reproductive traits in river breeds. In summary, this is the first study providing a large amount of genomic data of buffalo needed to describe their current genetic diversity and population structure, to scan the distinct selective pressures in river and swamp buffalo.

Methods

Sample Collection and Sequencing

We sampled a total of 98 buffaloes from different locations: China (81), Laos (5), Vietnam (4) and India (one Nili-Ravi, two Murrah, and five Indian buffaloes). Genomic DNA was extracted from ear tissue or blood samples using the standard phenol-chloroform protocol[78], amplified in genomic libraries with an average insert size of 500 bp, and sequenced (150-bp paired-end reads) on an Illumina HiSeq 2000. We also considered 23 available genome sequences from river buffalo, including 22 Mediterranean and one Murrah buffaloes. Additional details are provided in Supplementary tables 1 and 2. This study was approved by Institutional Animal Care and Use Committee of Northwest A&F University (Permit number: NWAFAC1019).

Alignments and Variant Identification

All cleaned reads were aligned to the reference genome (GCA_000471725.1) linked to "24+X+unplaced" pseudo-chromosomes (Supplementary Note 1, Supplementary table 3) using BWA-MEM with default settings [79]. Duplicate reads were filtered using Picard tools. The single nucleotide polymorphisms (SNPs) were detected with the Genome Analysis Toolkit (GATK, version 3.6-0-g89b7209) [80] and filtered using the "VariantFiltration" tool, as described in Supplementary Note 1.

Phylogenetic and Population Structure Analyses

The neighbour-joining (NJ) tree, principal component analysis (PCA), and ADMIXTURE methods were used to explore the genetic relationships among buffalo populations (Supplementary Note 2). An individual-based NJ tree based on the matrix of pairwise genetic distances from the autosomal SNP data of 121 buffaloes was constructed with PLINK (version 1.9) and visualized with FigTree. TreeMix program was used to construct a population-level phylogeny [81]. The principal component analysis (PCA) was performed using SmartPCA program in the package EIGENSOFT v5.0 [82] and eigenvectors' significance was detected by the Tracy-Widom test. The population genetic structure was estimated using ADMIXTURE v. 1.3.0 [83] considering from 2 to 5 clusters (K).

Y-chromosome and mitogenome phylogenies

After removing sites shared with female buffaloes, heterozygous sites and sites with a genotyping rate <5%, a total of 520 male-specific SNPs were used to construct the phylogenetic tree with BEAST 1.8.0 (Supplementary Note 3). A total of 98 mitochondrial genomes with an average coverage > 100X were assembled from the whole-genome resequencing data. Additional 107 whole mtDNA sequences were obtained from GenBank. A phylogenetic tree based on the final alignment was constructed using RaxML with the following parameters: -f a -x 123 -p 23 -# 100 -k -m 132 GTRGAMMA. The phylogenies were built using pegas [84].

Estimates of the effective population size and divergence time

A multiple sequential coalescent Markovian model (MSMC) was used to infer effective population sizes (N_e) and divergence times considering two samples with average coverage > 16× for each population. Autosomal SNPs of each sample were identified using GATK. After removing variant outliers (with extremely low or high coverage), all sites were phased using BEAGLE v. 4.1 [85]. The same high-coverage samples were also used to infer relative cross-coalescence rate (RCCR), considering a value of 0.5 as a reference to extrapolate split times between populations (samples). The time scale is calculated using an average generation time

of six years (g=6) and a mutation rate of $\mu_g = 1.26\times10^{-8}$ [86].

Genome-wide selective sweep test

To detect selective sweeps in swamp and river buffalo, we performed comparisons between these two types of buffaloes: (i) the swamp buffalo as the reference and the river buffalo as the object population; (ii) the river buffalo the reference and the swamp buffalo as the object population. A total of four methods were used: (i) The fixation index (F_{ST}) values [87] were calculated in sliding 50-kb windows with 20-kb steps along the autosomes using VCFtools [88]; (ii) High differences in genetic diversity (π ln ratio) were calculated with 50-kb sliding-windows and 20-kb steps along the autosomes using VCFtools and in-house scripts (iii) The cross-population composite likelihood ratio (XP-CLR) is a likelihood method for detecting selective sweeps that the change in allele frequency at the locus occurred too quickly to be due to random drift between two population [89]. We used non-overlapping sliding windows of 50 kb, maximum number of SNPs within each window as 600, and correlation level from which the SNPs contribution to XP-CLR result was down weighted to 0.95. (iv) We also performed the cross-population extended haplotype homozygosity (XP-EHH) test for every SNP using the default settings of the selscan v1.1 [90], which was designed to detect ongoing or nearly fixed selective sweeps by comparing haplotypes from two populations [91]. For the XP-EHH selection scan, our test statistic was the average normalized XP-EHH score in each 50-kb region. Significant genomic regions were identified by P-value < 0.005. Two or more methods showed outlier signals (P-value < 0.005) in overlapping regions and were therefore considered as the candidate selective regions. The KOBAS 3.0 (http://kobas.cbi.pku.edu.cn/anno_iden.php) was used to gain a better understanding of their biological functions and involved pathways.

Additional files

Supplementary figure 1. Genome-wide distribution of nucleotide diversity of buffaloes in six geographical regions in 50-kb sliding windows with 20-kb steps.

Supplementary figure 2. TreeMix relationships between 25 buffalo breeds.

Supplementary figure 3. Principal component analysis (PCA) of 196 river buffaloes, with PC1 plotted against PC2.

Supplementary figure 4. Principal component analysis (PCA) of swamp buffalo with PC1 plotted against PC2.

Supplementary figure 5. Model-based clustering of buffalo using the ADMIXTURE program with K = 2 to 5.

Supplementary figure 6. Maximum likelihood phylogeny of the Y-chromosome using 520 SNPs for 89 buffaloes.

Supplementary figure 7. Maximum likelihood phylogeny of the mitochondrial genome.

Supplementary figure 8. (a) The partial significant over-represented GO terms (Corrected *P*-value < 0.05) associated with immunity were showed by hierarchical graph. The colour bar is positively correlated to the Corrected *P*-value of the GO term. (b) Nonsynonymous SNP A226G (acid changed: T76A) located in the first exon of *BCL2L15*. Amino acids at this site are highly conserved in other mammals.

Supplementary figure 9. (a) The partial significant over-represented (Corrected *P*-value < 0.05) GO terms associated with nervous system were showed by hierarchical graph. The colour bar is positively correlated to the Corrected *P*-value of the GO term. (b) Nonsynonymous SNP C455T (acid changed: P152L) located in the first exon of *LOC102398542*. Amino acids at this site are highly conserved in other mammals.

Supplementary table 1. Overview of sample information and sequencing statistics.

Supplementary table 2. Summary information of the 121 individuals from 25 buffalo breeds.

Supplementary table 3. The information of the linked pseudo-chromosomes.

Supplementary table 4. Distribution of SNPs within various genomic regions.

Supplementary table 5. The $\theta\pi$ value for the buffalo population.

Supplementary table 6. Pairwise F_{ST} values calculated at continent scale.

Supplementary table 7. Tracy-Widom (TW) statistics and *P*-value for the ten first eigenvalues in the PCA of buffaloes.

Supplementary table 8. Cross-validation (CV) errors for ADMIXTURE ancestry models with K ranging from 2 to 5.

Supplementary Table 9. The genotype of 520 SNPs in the Y chromosome.

Supplementary table 10. Mapping results for 198 buffalo mitochondrial genomes and reference genomes.

Supplementary table 11. A summary of genes from F_{ST}.

Supplementary table 12. A summary of genes from XP-CLR (*P*-value < 0.5%) in river buffalo.

Supplementary table 13. A summary of genes from ln ratio(π_{swamp}/π_{river}) (*P*-value < 0.5%) in river buffalo.

Supplementary table 14. A summary of genes from XP-EHH in river buffalo.

Supplementary table 15. KEGG pathway analysis of candidate genes in river buffalo.

Supplementary table 16. Go enrichment of candidate genes in river buffalo.

Supplementary table 17. A summary of genes from XP-CLR (*P*-value < 0.5%) in swamp buffalo.

Supplementary table 18. A summary of genes from ln ratio(π_{river}/π_{swamp}) (*P*-value < 0.5%) in swamp buffalo.

Supplementary table 19. A summary of genes from XP-EHH in swamp buffalo.

Supplementary table 20. KEGG pathway analysis of candidate genes in swamp buffalo.

Supplementary table 21. Go enrichment of candidate genes in swamp buffalo.

Supplementary Note 1 Linking pseudo-chromosomes.

Supplementary Note 2 Population structure analysis.

Supplementary Note 3 Y-chromosome and Whole mitochondrial genome phylogeny.

Abbreviations

NJ tree: Neighbour-joining tree; PCA: principal component analysis; mtDNA: mitochondrial DNA; MSMC: multiple sequentially Markovian coalescent; Ne: effective population size; XP-CLR: the cross-population composite likelihood ratio; XP-EHH: the cross-population extended haplotype homozygosity; KEGG: Kyoto Encyclopedia of Genes and Genomes; GO: Gene Ontology.

Competing interests

We declare we have no competing interests.

Funding

The work was supported by the National Beef Cattle and Yak Industrial Technology System (CARS-37), Natural Science Foundation of China (31872317) to Chuzhao Lei, and National Thousand Youth Talents Plan to Yu Jiang; the Italian Ministry of Education, University and Research (MIUR), i.e. Dipartimenti di Eccellenza Program (2018-2022)-Dept. of Biology and Biotechnology "L. Spallanzani," University of Pavia (to A.A.).

Authors' contributions

Y.J. and C.Z.L. conceived and supervised the experiments. T.S., J.F.SH. performed majority of analysis with contributions from Q.M.CH. and ZH.Q.ZH. T.S. wrote the manuscript. N.B.CH., A. A. revised the manuscript. R.H.D., H.C.ZH, X.M.ZH, M.R.C., Y.ZH.H., X.Y.L., and H.CH. provided and prepared the samples. All authors reviewed the manuscript and gave final approval for publication.

Acknowledgements

We thank Wen Wang sharing the data of *Syncerus caffer*.

Figure legends

Figure 1. Population structure and relationships among buffaloes. (a) Geographic map indicating the origins of the buffalo breeds. (b) Neighbour-joining tree of buffaloes constructed using whole-genome autosomal SNP data. (c, d) Principal component analyses (PCA) showing PC1 against PC2 and PC1 against PC3, respectively. Each breed was labeled with different colors and shapes as showed in the top of Figure 1e. (e) Genetic structure of buffalo breeds using ADMIXTURE program with K = 2, 4. Population acronyms are explained in

Supplementary Tables 1 and 5.

Figure 2. Y-chromosome and mitogenome phylogenies. The width of the edges is proportional to the number of pairwise differences between the joined haplotypes. (a) Y-chromosome network using 520 SNPs. (b) Mitogenome network of swamp buffalo.

Figure 3. Demographic history and divergence of buffalo populations using MSMC. (a) Population size history inference of swamp and river buffalo based on four high-coverage haplotypes from Southwest China (SC), Southeast Asia (SEA), South Asia (SA), and Italy individuals (ITA). (b) Inferred relative cross-coalescence rates between pairs of populations over time based on the same four haplotypes.

Figure 4. Signatures of selective sweep regions at *PTPN22* and *MMSL22* genes in river buffalo. Different parameters were estimated for each gene (*PTPN22* and *MMSL22*): nucleotide diversity, degree of haplotype sharing across populations (a and c). A red arrow notes the specific gene region. A schematic structure of each gene (b and d) is also depicted with exons indicated by vertical bars and reference/alternative alleles noted with different colors (green/yellow) and combined to form different haplotypes (each with a specific haplotype frequency next to it). Non-synonymous SNPs are highlighted in gray.

Figure 5. Signatures of selective sweep regions at *HOMER1*, *HDAC9* and *GRIK2* genes in swamp buffalo. See the legend of Figure 4 for further details.

References

1. Fischer H and Ulbrich F. Chromosomes of the Murrah buffalo and its crossbreds with the Asiatic swamp buffalo (Bubalus bubalis). Zeitschrift für Tierzüchtung und Züchtungsbiologie. 1967;84 1-4:110-4. doi:10.1111/j.1439-0388.1967.tb01102.x.
2. Iannuzzi L. Standard karyotype of the river buffalo (Bubalus bubalis L., 2n = 50). Report of the committee for the standardization of banded karyotypes of the river buffalo. Cytogenetics and cell genetics. 1994;67 doi:10.1159/000133807.
3. Cockrill WR. The water buffalo: a review. The British veterinary journal. 1981;137 1:8-16. doi:10.1016/S0007-1935(17)31782-7.
4. Pietrusewsky M. The People of Ban Chiang: Bioarchaeology of the 1974 and 1975 Skeletons. In:

The International Conference on the Anniversary of the Discovery of the Ban Chiang Site 2016.

5. Cluttonbrock J. A natural history of domesticated mammals. Zoologica Africana. 1990;36 1:113-20. doi:10.1080/15627020.2001.11657122.
6. Nagarajan M, Nimisha K and Kumar S. Mitochondrial DNA Variability of Domestic River Buffalo (Bubalus bubalis) Populations: Genetic Evidence for Domestication of River Buffalo in Indian Subcontinent. Genome Biology & Evolution. 2015;7 5:496-503. doi:10.1093/gbe/evv067.
7. Yindee M, Vlamings BH, Wajjwalku W, Techakumphu M, Lohachit C, Sirivaidyapong S, et al. Y-chromosomal variation confirms independent domestications of swamp and river buffalo. Animal Genetics. 2010; doi:10.1111/j.1365-2052.2010.02020.x.
8. Lei CZ, Zhang W, Chen H, Lu F, Ge QL, Liu RY, et al. Two Maternal Lineages Revealed by Mitochondrial DNA D-loop Sequences in Chinese Native Water Buffaloes (Bubalus bubalis). Asian Australasian Journal of Animal Sciences. 2007;20 4:471-6.
9. Lei CZ, Zhang W, Chen H, Lu F, Liu RY, Yang XY, et al. Independent maternal origin of Chinese swamp buffalo (Bubalus bubalis). Animal Genetics. 2007;38 2:97-102. doi:10.1111/j.1365-2052.2007.01567.x.
10. Kumar S, Nagarajan M, Sandhu JS, Kumar N and Behl V. Phylogeography and domestication of Indian river buffalo. BMC Evolutionary Biology. 2007;7 1:186. doi:10.1186/1471-2148-7-186.
11. Kumar S, Nagarajan M, Sandhu J, Kumar N, Behl V and Nishanth G. Mitochondrial DNA analyses of Indian water buffalo support a distinct genetic origin of river and swamp buffalo. Animal genetics. 2007;38 3:227-32. doi:10.1111/j.1365-2052.2007.01602.x.
12. Wang S, Chen N, Capodiferro MR, Zhang T, Lancioni H, Zhang H, et al. Whole Mitogenomes Reveal the History of Swamp Buffalo: Initially Shaped by Glacial Periods and Eventually Modelled by Domestication. Scientific Reports. 2017;7 1:4708. doi:10.1038/s41598-017-04830-2.
13. Zhang Y, Lu Y, Yindee M, Li K-Y, Kuo H-Y, Ju Y-T, et al. Strong and stable geographic differentiation of swamp buffalo maternal and paternal lineages indicates domestication in the China/Indochina border region. Molecular Ecology. 2016;25 7:1530-50. doi:doi:10.1111/mec.13518.
14. Colli L, Milanesi M, Vajana E, Iamartino D, Bomba L, Puglisi F, et al. New Insights on Water Buffalo Genomic Diversity and Post-Domestication Migration Routes From Medium Density SNP Chip Data. Frontiers in Genetics. 2018;9 53 doi:10.3389/fgene.2018.00053.
15. Whitacre LK, Hoff JL, Schnabel RD, Albarella S, Ciotola F, Peretti V, et al. Elucidating the genetic basis of an oligogenic birth defect using whole genome sequence data in a non-model organism, Bubalus bubalis. Scientific Reports. 2017;7:39719. doi:10.1038/srep39719.
16. Borghese A. Buffalo production and research. Italian Journal of Animal Science. 2005;5 2.
17. Watson CJ and Burdon TG. Prolactin signal transduction mechanisms in the mammary gland: the role of the Jak/Stat pathway. Reviews of reproduction. 1996;1 1:1-5.
18. Shuai K and Liu B. Regulation of JAK-STAT signalling in the immune system. Nature reviews Immunology. 2003;3 11:900-11. doi:10.1038/nri1226.
19. O'Shea John J and Plenge R. JAK and STAT Signaling Molecules in Immunoregulation and Immune-Mediated Disease. Immunity. 2012;36 4:542-50. doi:10.1016/j.immuni.2012.03.014.
20. Liu L-L, Fang C and Liu W-J. Identification on novel locus of dairy traits of Kazakh horse in Xinjiang. Gene. 2018;677:105-10. doi:10.1016/j.gene.2018.07.009.

21. Li H, Wang Z, Moore SS, Schenkel FS and Stothard P. Genome-wide Scan For Positional And Functional Candidate Genes Affecting Milk Production Traits In Canadian Holstein Cattle. 2010.
22. Wei X, Li H, Yang J, Hao D, Dong D, Huang Y, et al. Circular RNA profiling reveals an abundant circLMO7 that regulates myoblasts differentiation and survival by sponging miR-378a-3p. Cell Death &Amp; Disease. 2017;8:e3153. doi:10.1038/cddis.2017.541.
23. Hardie LC, Vandehaar MJ, Tempelman RJ, Weigel KA, Armentano LE, Wiggans GR, et al. The genetic and biological basis of feed efficiency in mid-lactation Holstein dairy cows. Journal of Dairy Science. 2017;100 11 doi:10.3168/jds.2017-12604.
24. Kamprath K, Plendl W, Marsicano G, Deussing JM, Wurst W, Lutz B, et al. Endocannabinoids mediate acute fear adaptation via glutamatergic neurons independently of corticotropin-releasing hormone signaling. Genes, Brain and Behavior. 2009;8 2:203-11. doi:10.1111/j.1601-183X.2008.00463.x.
25. Mei C, Wang H, Liao Q, Wang L, Cheng G, Wang H, et al. Genetic Architecture and Selection of Chinese Cattle Revealed by Whole Genome Resequencing. Molecular Biology and Evolution. 2018;35 3:688-99. doi:10.1093/molbev/msx322.
26. Begovich AB, Carlton VEH, Honigberg LA, Schrodi SJ, Chokkalingam AP, Alexander HC, et al. A missense single-nucleotide polymorphism in a gene encoding a protein tyrosine phosphatase (PTPN22) is associated with rheumatoid arthritis. American journal of human genetics. 2004;75 2:330-7.
27. Bottini N, Musumeci L, Alonso A, Rahmouni S, Nika K, Rostamkhani M, et al. A functional variant of lymphoid tyrosine phosphatase is associated with type I diabetes. Nature Genetics. 2004;36:337.
28. Kyogoku C, Langefeld CD, Ortmann WA, Lee A, Selby S, Carlton VEH, et al. Genetic association of the R620W polymorphism of protein tyrosine phosphatase PTPN22 with human SLE. American journal of human genetics. 2004;75 3:504-7.
29. Ban Y, Tozaki T and Nakano Y. Association Studies of the GPR103 and BCL2L15 Genes in Autoimmune Thyroid Disease in the Japanese Population. Frontiers in Endocrinology. 2016;7 92 doi:10.3389/fendo.2016.00092.
30. Ibeagha-Awemu EM, Peters SO, Akwanji KA, Imumorin IG and Zhao X. High density genome wide genotyping-by-sequencing and association identifies common and low frequency SNPs, and novel candidate genes influencing cow milk traits. Scientific Reports. 2016;6:31109. doi:10.1038/srep31109.
31. Brym P, Bojarojćnosowicz B, Oleński K, Hering DM, Ruść A, Kaczmarczyk E, et al. Genome-wide association study for host response to bovine leukemia virus in Holstein cows. Vet Immunol Immunopathol. 2016;175:24-35.
32. Marai IFM and Haeeb AAM. Buffalo's biological functions as affected by heat stress-A review. Livestock Science. 2010;127 2:89-109. doi:10.1016/j.livsci.2009.08.001.
33. Belhadj Slimen I, Najar T, Ghram A and Abdrrabba M. Heat stress effects on livestock: molecular, cellular and metabolic aspects, a review. Journal of Animal Physiology and Animal Nutrition. 2016;100 3:401-12. doi:10.1111/jpn.12379.
34. Saredi G, Huang H, Hammond CM, Alabert C, Bekker-Jensen S, Forne I, et al. H4K20me0 marks post-replicative chromatin and recruits the TONSL–MMS22L DNA repair complex. Nature. 2016;534:714. doi:10.1038/nature18312.

35. Ben-Aroya S, Agmon N, Yuen K, Kwok T, McManus K, Kupiec M, et al. Proteasome Nuclear Activity Affects Chromosome Stability by Controlling the Turnover of Mms22, a Protein Important for DNA Repair. PLOS Genetics. 2010;6 2:e1000852. doi:10.1371/journal.pgen.1000852.

36. George I, Wenqi W and Minli W. DNA double strand break repair inhibition as a cause of heat radiosensitization: re-evaluation considering backup pathways of NHEJ. International Journal of Hyperthermia the Official Journal of European Society for Hyperthermic Oncology North American Hyperthermia Group. 2008;24 1:17. doi:10.1080/02656730701784782.

37. Kantidze OL, Velichko AK, Luzhin AV and Razin SV. Heat Stress-Induced DNA Damage. Acta naturae. 2016;8 2:75-8.

38. Branzei D and Foiani M. Maintaining genome stability at the replication fork. Nature Reviews Molecular Cell Biology. 2010;11:208. doi:10.1038/nrm2852.

39. Filippo JS, Sung P and Klein H. Mechanism of Eukaryotic Homologous Recombination. Annual Review of Biochemistry. 2008;77 1:229-57. doi:10.1146/annurev.biochem.77.061306.125255.

40. Duro E, Lundin C, Ask K, Sanchez-Pulido L, MacArtney TJ, Toth R, et al. Identification of the MMS22L-TONSL Complex that Promotes Homologous Recombination. Molecular Cell. 2010;40 4:632-44. doi:10.1016/j.molcel.2010.10.023.

41. Kasai H, Matsuzaki M, Noguchi J, Yasumatsu N and Nakahara H. Structure–stability–function relationships of dendritic spines. Trends in Neurosciences. 2003;26 7:360-8. doi:10.1016/S0166-2236(03)00162-0.

42. Zhang H and Macara IG. The polarity protein PAR-3 and TIAM1 cooperate in dendritic spine morphogenesis. Nature Cell Biology. 2006;8 3:227-37. doi:10.1038/ncb1368.

43. Tolias KF, Bikoff JB, Kane CG, Tolias CS, Hu L and Greenberg ME. The Rac1 guanine nucleotide exchange factor Tiam1 mediates EphB receptor-dependent dendritic spine development. Proceedings of the National Academy of Sciences. 2007;104 17:7265. doi:10.1073/pnas.0702044104.

44. Tolias KF, Bikoff JB, Burette A, Paradis S, Harrar D, Tavazoie S, et al. The Rac1-GEF Tiam1 Couples the NMDA Receptor to the Activity-Dependent Development of Dendritic Arbors and Spines. Neuron. 2005;45 4:525-38. doi:10.1016/j.neuron.2005.01.024.

45. Li M, Luo X-J, Xiao X, Shi L, Liu X-Y, Yin L-D, et al. Analysis of common genetic variants identifies RELN as a risk gene for schizophrenia in Chinese population. The World Journal of Biological Psychiatry. 2013;14 2:91-9. doi:10.3109/15622975.2011.587891.

46. Abdolmaleky HM, Cheng K-h, Russo A, Smith CL, Faraone SV, Wilcox M, et al. Hypermethylation of the reelin (RELN) promoter in the brain of schizophrenic patients: A preliminary report. American Journal of Medical Genetics Part B: Neuropsychiatric Genetics. 2005;134B 1:60-6. doi:10.1002/ajmg.b.30140.

47. Zhou Z, Hu Z, Zhang L, Hu Z, Liu H, Liu Z, et al. Identification of RELN variation p.Thr3192Ser in a Chinese family with schizophrenia. Scientific Reports. 2016;6:24327. doi:10.1038/srep24327.

48. Hennah W, Thomson P, Peltonen L and Porteous D. Genes and Schizophrenia: Beyond Schizophrenia: The Role of DISC1 in Major Mental Illness. Schizophrenia Bulletin. 2006;32 3:409-16. doi:10.1093/schbul/sbj079.

49. Mackie S, Millar JK and Porteous DJ. Role of DISC1 in neural development and schizophrenia. Current Opinion in Neurobiology. 2007;17 1:95-102. doi:10.1016/j.conb.2007.01.007.

50. Kamiya A, Kubo K-i, Tomoda T, Takaki M, Youn R, Ozeki Y, et al. A schizophrenia-associated mutation of DISC1 perturbs cerebral cortex development. Nature Cell Biology. 2005;7 12:1167-78. doi:10.1038/ncb1328.

51. Katzman A and Alberini CM. NLGN1 and NLGN2 in the prefrontal cortex: their role in memory consolidation and strengthening. Current Opinion in Neurobiology. 2018;48:122-30. doi:10.1016/j.conb.2017.12.003.

52. Balan S, Yamada K, Hattori E, Iwayama Y, Toyota T, Ohnishi T, et al. Population-Specific Haplotype Association of the Postsynaptic Density Gene DLG4 with Schizophrenia, in Family-Based Association Studies. PLOS ONE. 2013;8 7:e70302. doi:10.1371/journal.pone.0070302.

53. Cheng M-C, Lu C-L, Luu S-U, Tsai H-M, Hsu S-H, Chen T-T, et al. Genetic and Functional Analysis of the DLG4 Gene Encoding the Post-Synaptic Density Protein 95 in Schizophrenia. PLOS ONE. 2010;5 12:e15107. doi:10.1371/journal.pone.0015107.

54. Sugo N, Oshiro H, Takemura M, Kobayashi T, Kohno Y, Uesaka N, et al. Nucleocytoplasmic translocation of HDAC9 regulates gene expression and dendritic growth in developing cortical neurons. European Journal of Neuroscience. 2010;31 9:1521-32. doi:10.1111/j.1460-9568.2010.07218.x.

55. Zhang S, Xu H, Liu X, Yang Q, Pan C, Lei C, et al. The muscle development transcriptome landscape of ovariectomized goat. Royal Society Open Science. 2017;4 12:171415. doi:10.1098/rsos.171415.

56. Haberland M, Arnold MA, McAnally J, Phan D, Kim Y and Olson EN. Regulation of HDAC9 Gene Expression by MEF2 Establishes a Negative-Feedback Loop in the Transcriptional Circuitry of Muscle Differentiation. Molecular and Cellular Biology. 2007;27 2:518. doi:10.1098/rsos.171415.

57. Mei C, Wang H, Liao Q, Khan R, Raza SHA, Zhao C, et al. Genome-wide analysis reveals the effects of artificial selection on production and meat quality traits in Qinchuan cattle. Genomics. 2018; doi:10.1016/j.ygeno.2018.09.021.

58. Haberland M, Montgomery RL and Olson EN. The many roles of histone deacetylases in development and physiology: implications for disease and therapy. Nature Reviews Genetics. 2009;10:32. doi:10.1038/nrg2485.

59. Potthoff MJ, Wu H, Arnold MA, Shelton JM, Backs J, McAnally J, et al. Histone deacetylase degradation andMEF2 activation promote the formation of slow-twitch myofibers. The Journal of Clinical Investigation. 2007;117 9:2459-67. doi:10.1172/JCI31960.

60. Szumlinski KK, Lominac KD, Kleschen MJ, Oleson EB, Dehoff MH, Schwartz MK, et al. Behavioral and neurochemical phenotyping of Homer1 mutant mice: possible relevance to schizophrenia. Genes, Brain and Behavior. 2005;4 5:273-88. doi:10.1111/j.1601-183X.2005.00120.x.

61. Spellmann I, Rujescu D, Musil R, Mayr A, Giegling I, Genius J, et al. Homer-1 polymorphisms are associated with psychopathology and response to treatment in schizophrenic patients. Journal of Psychiatric Research. 2011;45 2:234-41. doi:10.1016/j.jpsychires.2010.06.004.

62. Rietschel M, Mattheisen M, Frank J, Treutlein J, Degenhardt F, Breuer R, et al. Genome-Wide Association-, Replication-, and Neuroimaging Study Implicates HOMER1 in the Etiology of Major Depression. Biological Psychiatry. 2010;68 6:578-85. doi:10.1016/j.biopsych.2010.05.038.

63. Jaubert PJ, Golub MS, Lo YY, Germann SL, Dehoff MH, Worley PF, et al. Complex, multimodal behavioral profile of the Homer1 knockout mouse. Genes, Brain and Behavior. 2007;6 2:141-54.

doi:10.1111/j.1601-183X.2006.00240.x.
64. Gerstein H, O'Riordan K, Osting S, Schwarz M and Burger C. Rescue of synaptic plasticity and spatial learning deficits in the hippocampus of Homer1 knockout mice by recombinant Adeno-associated viral gene delivery of Homer1c. Neurobiology of Learning and Memory. 2012;97 1:17-29. doi:10.1016/j.nlm.2011.08.009.
65. Inoue N, Nakao H, Migishima R, Hino T, Matsui M, Hayashi F, et al. Requirement of the immediate early gene vesl-1S/homer-1a for fear memory formation. Molecular Brain. 2009;2 1:7. doi:10.1186/1756-6606-2-7.
66. Klugmann M and Szumlinski KK. Targeting Homer genes using adeno-associated viral vector: lessons learned from behavioural and neurochemical studies. Behavioural pharmacology. 2008;19 5-6:485-500. doi:10.1097/FBP.0b013e32830c369f.
67. Stiber JA, Zhang Z-S, Burch J, Eu JP, Zhang S, Truskey GA, et al. Mice Lacking Homer 1 Exhibit a Skeletal Myopathy Characterized by Abnormal Transient Receptor Potential Channel Activity. Molecular and Cellular Biology. 2008;28 8:2637-47. doi:10.1128/MCB.01601-07.
68. Chapuis J, Hansmannel F, Gistelinck M, Mounier A, Van Cauwenberghe C, Kolen KV, et al. Increased expression of BIN1 mediates Alzheimer genetic risk by modulating tau pathology. Molecular Psychiatry. 2013;18:1225. doi:10.1038/mp.2013.1.
69. Wijsman EM, Pankratz ND, Choi Y, Rothstein JH, Faber KM, Cheng R, et al. Genome-Wide Association of Familial Late-Onset Alzheimer's Disease Replicates BIN1 and CLU and Nominates CUGBP2 in Interaction with APOE. PLOS Genetics. 2011;7 2:e1001308. doi:10.1371/journal.pgen.1001308.
70. Yu L, Chibnik LB, Srivastava GP, Pochet N, Yang J, Xu J, et al. Association of Brain DNA Methylation in SORL1, ABCA7, HLA-DRB5, SLC24A4, and BIN1 With Pathological Diagnosis of Alzheimer DiseaseBrain DNA Methylation and Pathological AD DiagnosisBrain DNA Methylation and Pathological AD Diagnosis. JAMA Neurology. 2015;72 1:15-24. doi:10.1001/jamaneurol.2014.3049.
71. Lee E, Marcucci M, Daniell L, Pypaert M, Weisz OA, Ochoa G-C, et al. Amphiphysin 2 (Bin1) and T-Tubule Biogenesis in Muscle. Science. 2002;297 5584:1193. doi:10.1126/science.1071362.
72. Razzaq A, Robinson IM, McMahon HT, Skepper JN, Su Y, Zelhof AC, et al. Amphiphysin is necessary for organization of the excitation-contraction coupling machinery of muscles, but not for synaptic vesicle endocytosis in Drosophila. Genes & development. 2001;15 22:2967-79. doi:10.1101/gad.207801.
73. Butler MH, David C, Ochoa G-C, Freyberg Z, Daniell L, Grabs D, et al. Amphiphysin II (SH3P9; BIN1), a Member of the Amphiphysin/Rvs Family, Is Concentrated in the Cortical Cytomatrix of Axon Initial Segments and Nodes of Ranvier in Brain and around T Tubules in Skeletal Muscle. The Journal of Cell Biology. 1997;137 6:1355. doi:10.1083/jcb.137.6.1355.
74. Motazacker MM, Rost BR, Hucho T, Garshasbi M, Kahrizi K, Ullmann R, et al. A Defect in the Ionotropic Glutamate Receptor 6 Gene (GRIK2) Is Associated with Autosomal Recessive Mental Retardation. The American Journal of Human Genetics. 2007;81 4:792-8. doi:doi.org/10.1086/521275.
75. Ko S, Zhao M-G, Toyoda H, Qiu C-S and Zhuo M. Altered Behavioral Responses to Noxious Stimuli and Fear in Glutamate Receptor 5 (GluR5)- or GluR6-Deficient Mice. The Journal of Neuroscience.

2005;25 4:977. doi:10.1523/JNEUROSCI.4059-04.2005.
76. Shaltiel G, Maeng S, Malkesman O, Pearson B, Schloesser RJ, Tragon T, et al. Evidence for the involvement of the kainate receptor subunit GluR6 (GRIK2) in mediating behavioral displays related to behavioral symptoms of mania. Molecular Psychiatry. 2008;13:858. doi:10.1038/mp.2008.20.
77. Carneiro M, Rubin C-J, Di Palma F, Albert FW, Alföldi J, Barrio AM, et al. Rabbit genome analysis reveals a polygenic basis for phenotypic change during domestication. Science. 2014;345 6200:1074. doi:10.1126/science.1253714.
78. Green MR and Sambrook J. Molecular Cloning: A Laboratory Manual (Fourth Edition): Three-Volume Set. Cold Spring Harbor Laboratory Pr. 2012.
79. Li H and Durbin R. Fast and accurate short read alignment with Burrows–Wheeler transform. Bioinformatics. 2009;25 14:1754-60. doi:10.1093/bioinformatics/btp324.
80. Nekrutenko A and Taylor J. Next-generation sequencing data interpretation: enhancing reproducibility and accessibility. Nature Reviews Genetics. 2012;13 9:667-72. doi:10.1038/nrg3305.
81. Pickrell JK and Pritchard JK. Inference of Population Splits and Mixtures from Genome-Wide Allele Frequency Data. PLOS Genetics. 2012;8 11:e1002967. doi:10.1371/journal.pgen.1002967.
82. Patterson N, Price AL and Reich D. Population Structure and Eigenanalysis. PLOS Genetics. 2006;2 12:e190. doi:10.1371/journal.pgen.0020190.
83. Alexander DH, Novembre J and Lange K. Fast model-based estimation of ancestry in unrelated individuals. Genome Research. 2009;19 9:1655-64. doi:10.1101/gr.094052.109.
84. Paradis E. pegas: an R package for population genetics with an integrated–modular approach. Bioinformatics. 2010;26 3:419-20. doi:10.1093/bioinformatics/btp696.
85. Browning SR and Browning BL. Rapid and Accurate Haplotype Phasing and Missing-Data Inference for Whole-Genome Association Studies By Use of Localized Haplotype Clustering. American Journal of Human Genetics. 2007;81 5:1084-97. doi:10.1086/521987.
86. Chen N, Cai Y, Chen Q, Li R, Wang K, Huang Y, et al. Whole-genome resequencing reveals world-wide ancestry and adaptive introgression events of domesticated cattle in East Asia. Nature Communications. 2018;9 1:2337. doi:10.1038/s41467-018-04737-0.
87. Weir BS and Cockerham CC. Estimating F-statistics for the analysis of population-structure. Evolution. 1984;38 6:1358-70. doi:10.2307/2408641.
88. Danecek P, Auton A, Abecasis G, Albers CA, Banks E, DePristo MA, et al. The variant call format and VCFtools. Bioinformatics. 2011;27 15:2156-8. doi:10.1093/bioinformatics/btr330.
89. Chen H, Patterson N and Reich D. Population differentiation as a test for selective sweeps. Genome Research. 2010;20 3:393-402.
90. Szpiech ZA and Hernandez RD. selscan: An Efficient Multithreaded Program to Perform EHH-Based Scans for Positive Selection. Molecular Biology and Evolution. 2014;31 10:2824-7. doi:10.1093/molbev/msu211.
91. Sabeti PC, Varilly P, Fry B, Lohmueller J, Hostetter E, Cotsapas C, et al. Genome-wide detection and characterization of positive selection in human populations. Nature. 2007;449:913. doi:10.1038/nature06250.

Figure Click here to download Figure figure 1.tif

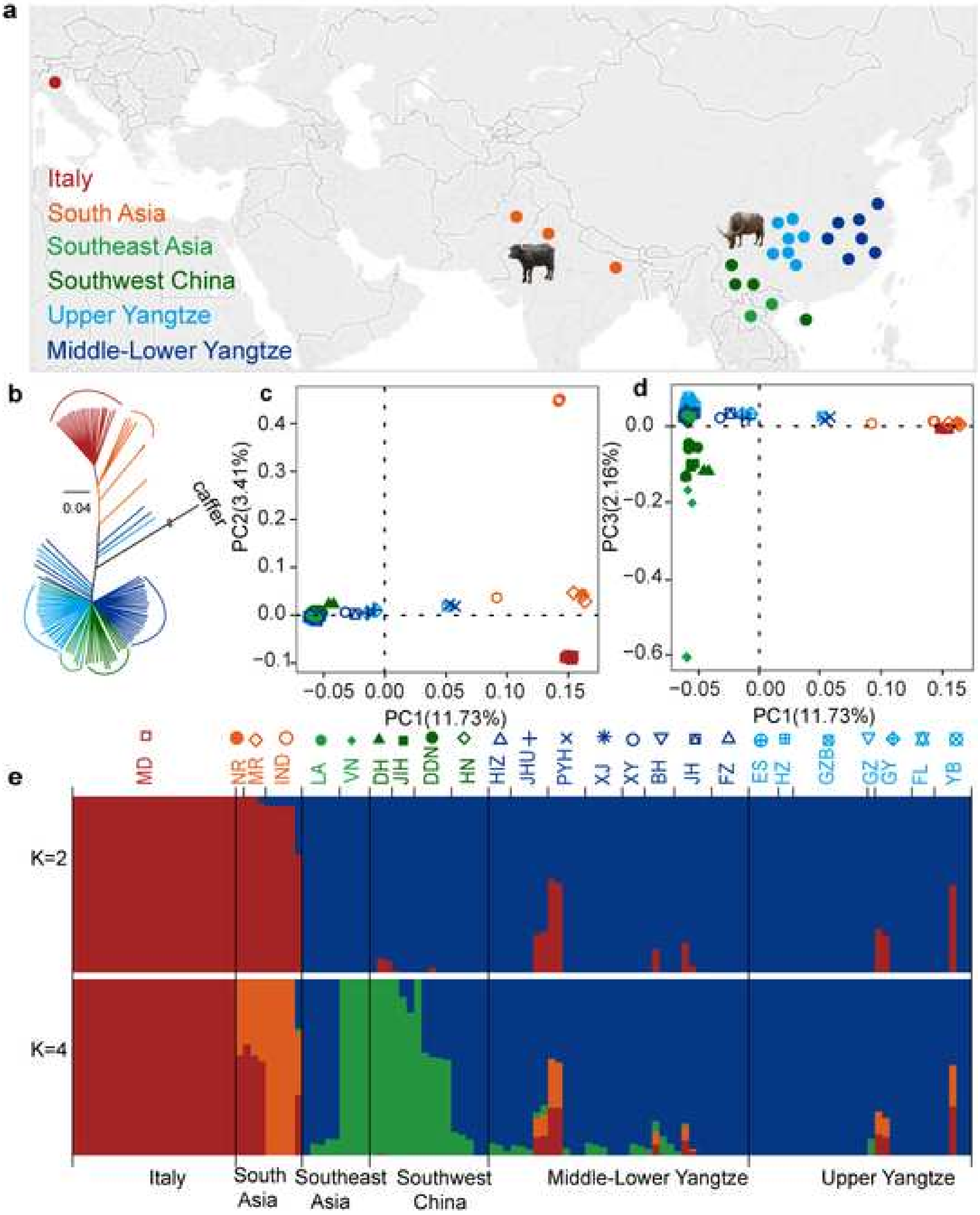

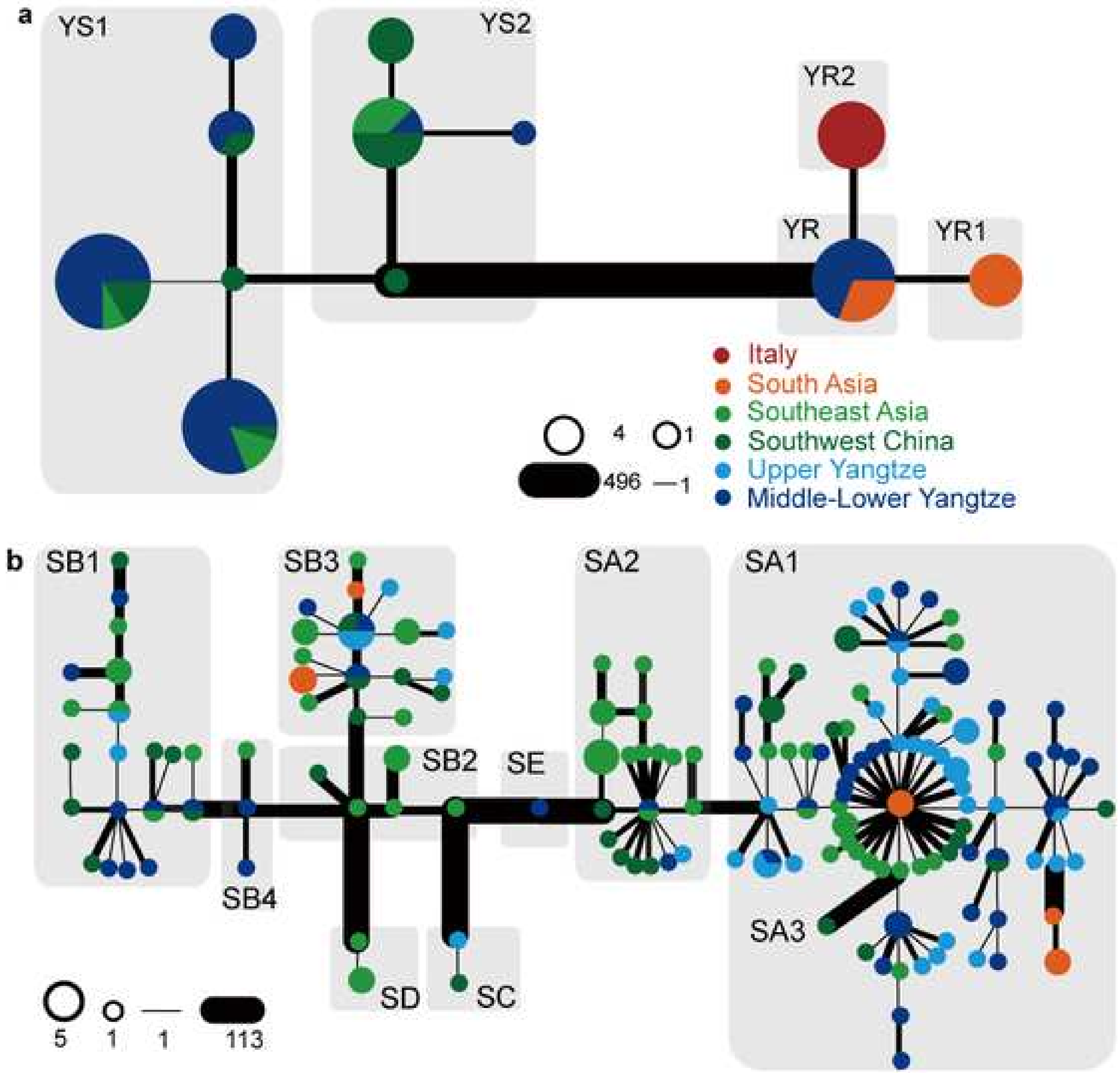
a
YS1
YS2
YR2
YR
YR1
Italy
South Asia
Southeast Asia
Southwest China
Upper Yangtze
Middle-Lower Yangtze
4
1
496
1
b
SB1
SB3
SA2
SA1
SB2
SE
SB4
SA3
SD
SC
5
1
1
113

Click here to download Figure figure 3.tif

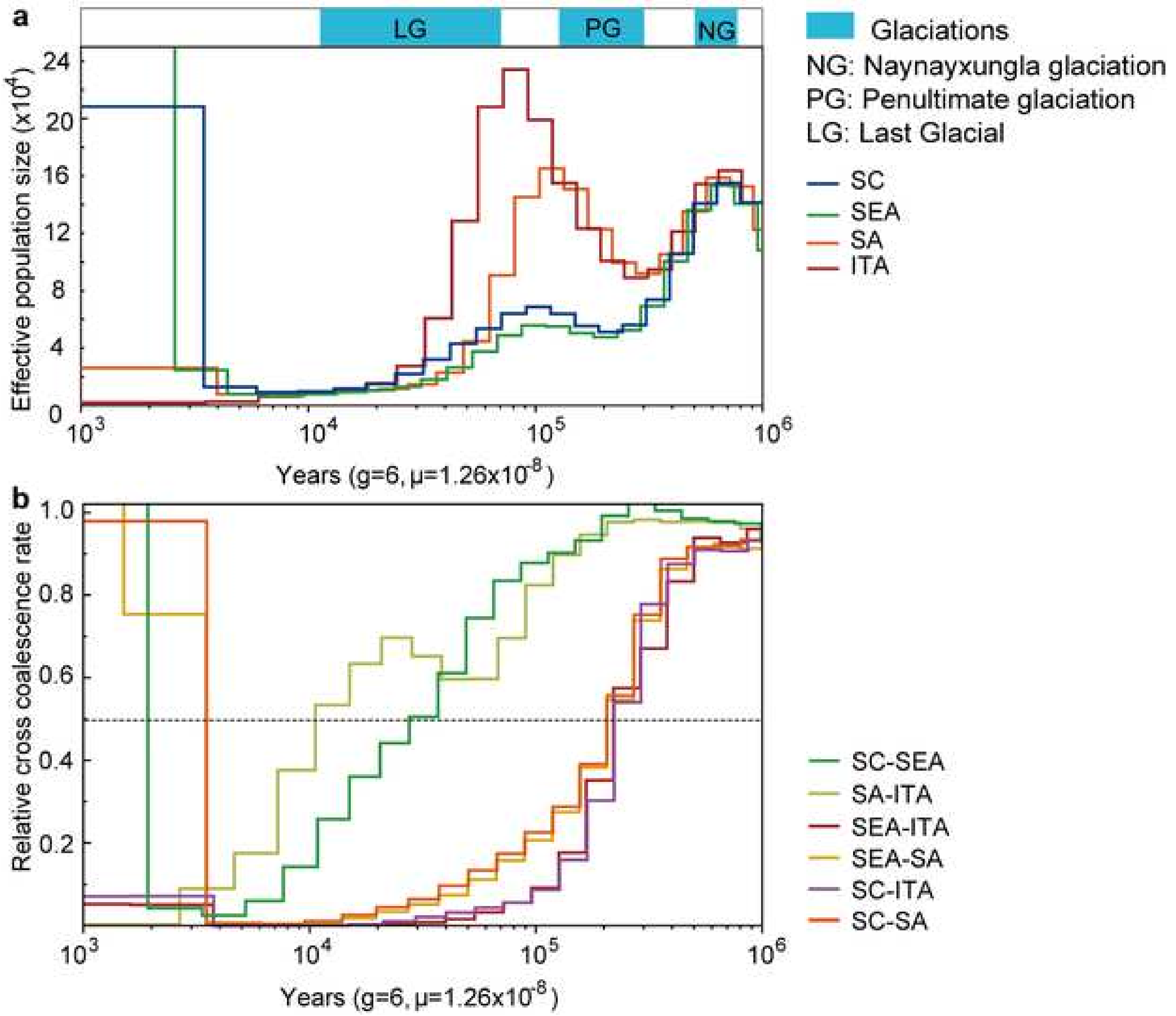

 Click here to download Figure figure4.tif

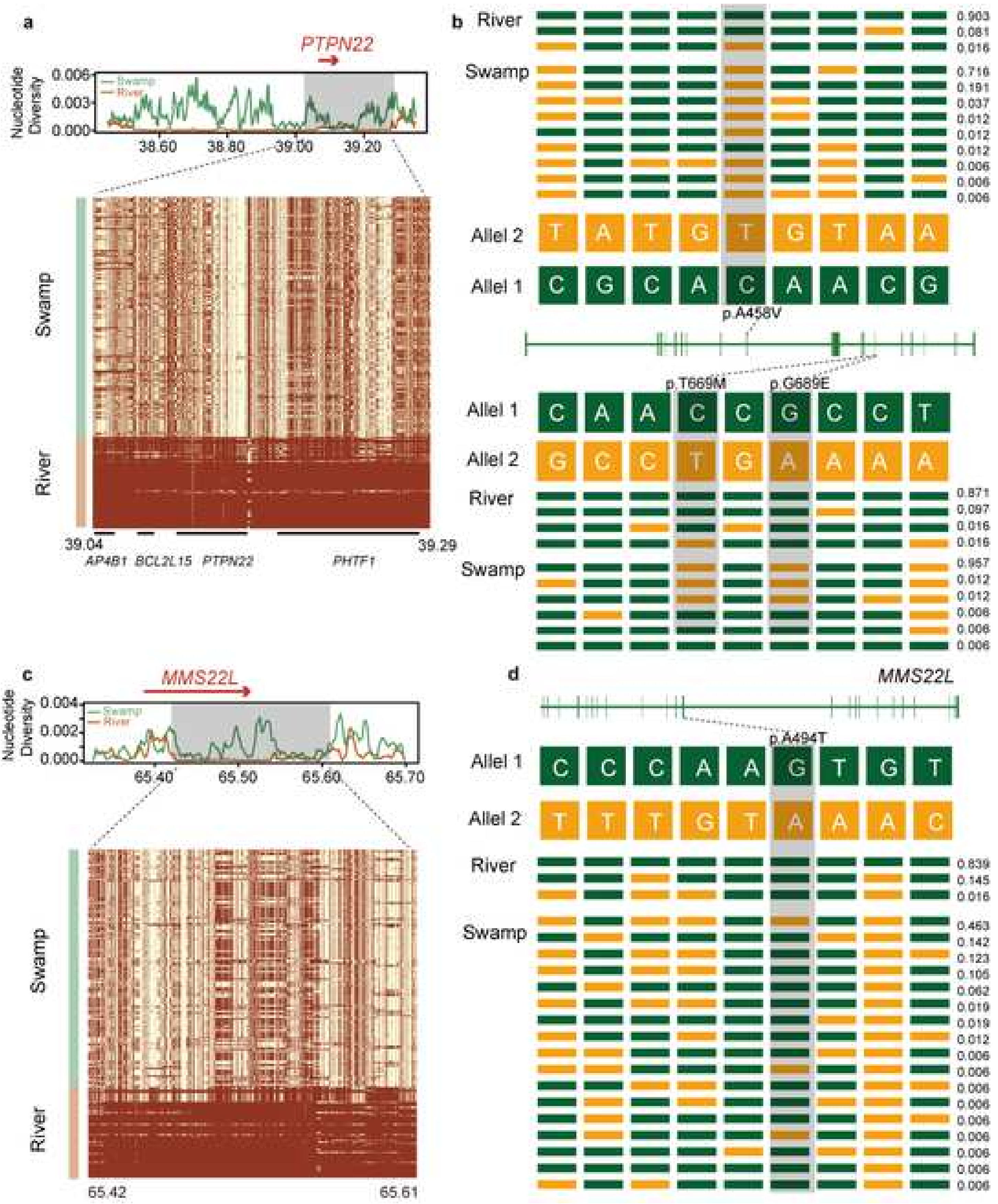

Click here to download Figure figure5.tif

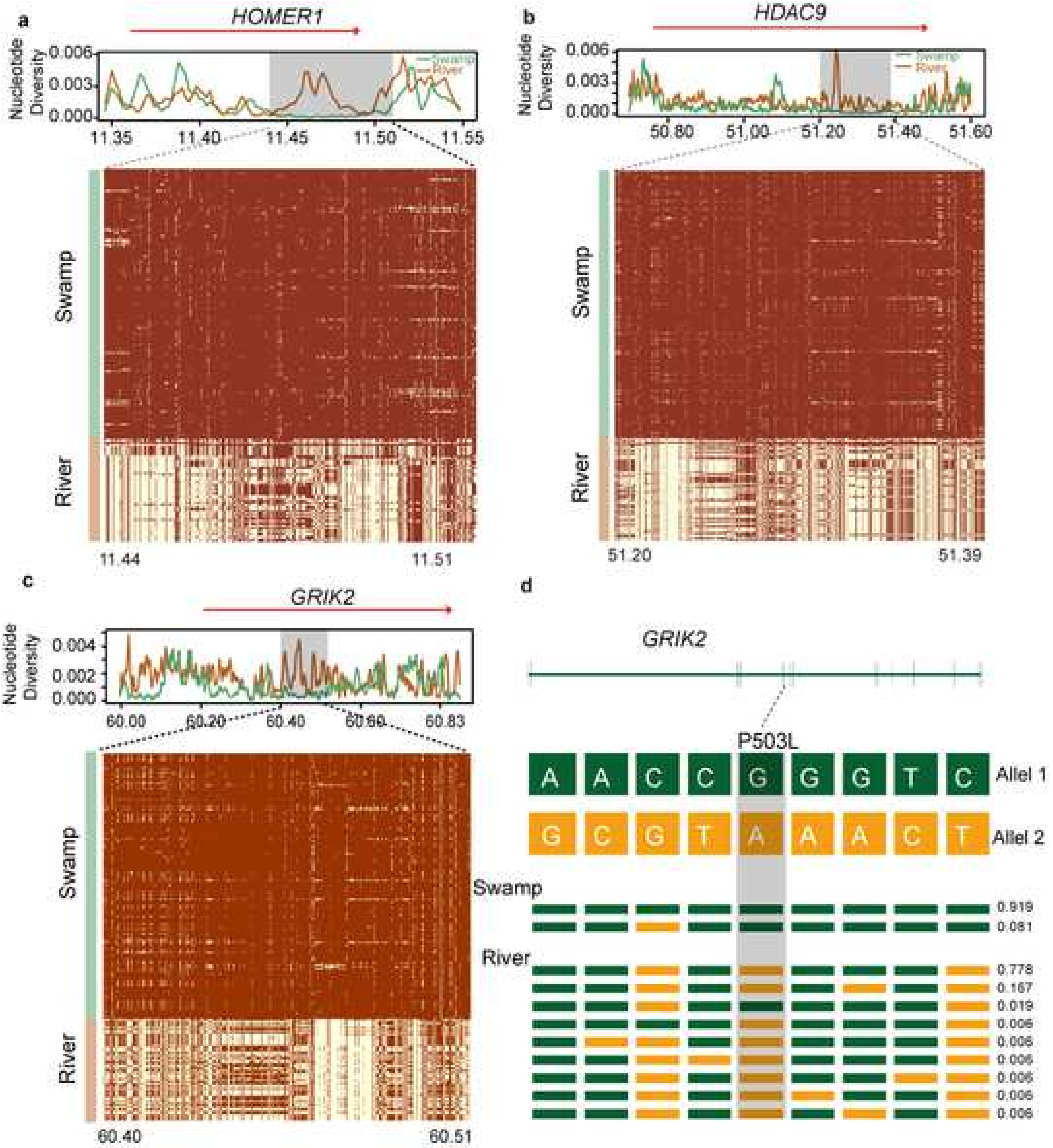

Supplementary Tables

Click here to access/download
Supplementary Material
Supplementary Tables.xlsx

Click here to access/download
Supplementary Material
Supplementary Materials.pdf
