# Supplementary material for: Genomic analyses reveal distinct genetic architectures and selective pressures in buffaloes
Source: Gigascience. 2020 Feb 21;9(2):giz166. doi: 10.1093/gigascience/giz166 (PMC7033652; doi:10.1093/gigascience/giz166)
Supplement: giz166_GIGA-D-19-00183_Revision_1 [file giz166_giga-d-19-00183_revision_1.pdf]

GigaScience

Genomic Analyses Reveal Distinct Genetic Architectures and Selective Pressures in Buffaloes

--Manuscript Draft--

**Manuscript Number:**	GIGA-D-19-00183R1
**Full Title:**	Genomic Analyses Reveal Distinct Genetic Architectures and Selective Pressures in Buffaloes
**Article Type:**	Research
**Funding Information:**	National Beef Cattle and Yak Industrial Technology System (CARS-37) — Prof. Chuzhao Lei<br>Natural Science Foundation of China (31872317) — Prof. Chuzhao Lei<br>National Thousand Youth Talents Plan (none) — Prof. Yu Jiang<br>Dipartimenti di Eccellenza Program (none) — Not applicable
**Abstract:**	Backgroud<br>The domestic buffalo (Bubalus bubalis) is an essential farm animal in tropical and subtropical regions, whose genomic diversity is yet to be fully discovered.<br>Results<br>In this study, we describe the demographic events and selective pressures of buffalo by analyzing 121 whole genomes (98 newly reported) from 25 swamp and river buffalo breeds. Both uniparental and biparental markers were investigated to provide the final scenario. The ancestors of swamp and river buffalo diverged ~0.23 Mya and then experienced independent demographic histories. They were domesticated in different regions, the swamp buffalo at the border between Southwest China and Southeast Asia, while the river buffalo in South Asia. The domestic stocks migrated to other regions and further differentiated, as testified by (at least) two ancestral components identified in each subspecies. Different signals of selective pressures were also detected in these two types of buffalo. The swamp buffalo, historically used as a draft animal, shows selection signatures in genes associated with nervous system, while in river dairy breeds, genes under selection are related to heat-stress and immunity.<br>Conclusions<br>Our findings substantially expand the catalogue of genetic variants in buffalo, reveal new insights into the evolutionary history and distinct selective pressures in river and swamp buffalo.
**Corresponding Author:**	Yu Jiang, Ph.D<br>Northwest Agriculture and Forestry University<br>Yangling, Shaanxi CHINA
**Corresponding Author Secondary Information:**	
**Corresponding Author's Institution:**	Northwest Agriculture and Forestry University
**Corresponding Author's Secondary Institution:**	
**First Author:**	Ting Sun
**First Author Secondary Information:**	
**Order of Authors:**	Ting Sun
	Jiafei Shen
	Ningbo Chen
	Qiuming Chen
	Ruihua Dang

	Zhuqing Zheng
	Hucai Zhang
	Xiaoming Zhang
	Shaoqiang Wang
	Tao Zhang
	Hongzhao Lu
	Yun Ma
	Yutang Jia
	Marco Rosario Capodiferro
	Yongzhen Huang
	Xianyong Lan
	Hong Chen
	Alessandro Achilli
	Yu Jiang, Ph.D
	Chuzhao Lei
**Order of Authors Secondary Information:**	

Response to Reviewers:

Response to Reviewers' comments

Genomic Analyses Reveal Distinct Genetic Architectures and Selective Pressures in Buffaloes
GigaScience
Dear Zhou,
Thank you very much for handling our manuscript Manuscript ID entitled " Genomic Analyses Reveal Distinct Genetic Architectures and Selective Pressures in Buffaloes" (GIGA-D-19-00183). We appreciate all the comments from the reviewers, which helped us to improve our manuscript. We have now revised the manuscript according to the reviewers' comments and your instructions.
We addressed the comments and questions of the reviewers as explained below; we have modified the manuscript according to the suggestions of the reviewers. Revised sentences are marked in red in the paper.

Reviewer reports:
[Reviewer #1:]
Karyotyping for confirmation of riverine, swamp or hybrid buffaloes has not been done. Then, how, the classification of the groups has been done?
Response: Riverine buffaloes and swamp buffaloes belong to two types of buffalo, with divergent genomes and karyotypes. According to the results of NJ tree, ML tree, ADMIXTURE, and PCA using the whole-genome SNP information, the buffaloes can be clearly divided into river buffaloes, swamp buffaloes, and hybrid buffaloes. Furthermore, the uniparental markers (Y chromosomal and mitochondrial DNA) have been also used as a further confirmation.

No out group has been taken in the phylogenetic analysis.
Response: Actually, we used the Syncerus caffer as outgroup to perform the phylogenetic analysis as shown in the Figure 1b and Supplementary figure 2. This information has been added in the revised text (Line 107 - Line 108).

There is no mention about the parameters used in the various tools in the study.
Response: The necessary parameters are provided in the methods and the supplementary notes, please check.

Many of the notations used in the supplementary need to be abbreviated.
Response: Whenever possible, we have now abbreviated the notions, as reported in supplementary figures 2-5 and supplementary Notes. Please check.

Powered by Editorial Manager® and ProduXion Manager® from Aries Systems Corporation

Selective sweep regions usually show lower nucleotide diversity and high level of haplotype homozygosity. Please delete the general statements about the selective sweep.
Response: We completely agree with the reviewer. The statement has been deleted accordingly.

What is meant by candidate selective sweep regions?
Response: They indicate genomic regions in which based on our analysis it would be more likely to identify signs of selective sweep. These regions show reduction of variation due to genetic hitchhiking with a site under selection. In other words, a selective sweep can occur when a rare or previously non-existing allele that increases the fitness of the carrier (relative to other members of the population) increases rapidly in frequency. A selective sweep due to a strongly selected allele, which arose on a single genomic background therefore results in a region of the genome with a large reduction of genetic variation in that chromosome region. Such detected regions were identified as selective regions. These selective regions containing genes which may be associated with specific phenotype, adaption, specific characters of a specie/breed/population, etc. Therefore, we called candidate selective regions.

Data need to be submitted in the public domain
Response: We have submitted the data to the NCBI Short Read Archive under the BioProject accession number PRJNA547460. We have now added the information in the main text (Line 97-99), please check.

Reviewer #2:
In this manuscript Sun et al. analysed 121 buffalo whole genomes, out of which 98 genomes are newly reported. The authors provide a detailed description of the genetic diversity both addressing demographic questions and investigating selection signals. First, the authors assessed the split time between the ancestor of swamp and river buffalo and described the possible domestication scenarios for both of them. Moreover, they analysed uniparental markers which support their previous results even though they did not have mtDNA sequences for river buffalos. Finally, the authors investigated possible signals of selection using different approaches and they identified distinctive genes under selection in river and swamp buffalo.
Overall, the manuscript represents a comprehensive and detailed description of the genetic diversity, demographic histories and selection signals for river and swamp buffalo. I am happy with both the data generation and the analyses. Multiple approaches have also been used to confirm their results. However, I personally feel that the authors reported too many technical details regarding the amount the data generated, specifically in the paragraph titled "Data Description". I would rather move these technical details to the supplementary information and incorporate the estimates of genetic diversity into the next paragraph called "Analysis".
Response: Thanks for your kind suggestion. Due to the "Data Description" is a needed part according to the guideline of GigaScience. So, we only retain the brief information in the main text (line 96 - 101), and other information removed to the supplementary (supplementary: line 39 -line 45). Please check.

Considering the interesting results of this study, I would suggest the authors to highlight the main messages of the story around swamp and river buffalos and leave the more technical and descriptive sections to the supplementary information. The current version of the manuscript is a precise detailed description of all analyses performed but it would be nice to have the manuscript more structured and centred around the interesting story of the river and swamp buffalos. The authors could restructure some paragraphs focusing more on the main messages of this study and providing the analyses performed as evidence to support such messages. In this way the paper would be more engaging and easier to read.
Response: Thanks for your kind suggestion. We have revised the manuscript in order to make it more fluent, therefore some information has been moved to the supplementary material (Line 39-57) as suggested. We also tried our best to restructure some paragraphs (Line 244- Line 258, Line 274-279, Line 197-302), please check.

	Regarding the mtDNA: I would like the authors to clarify why they were not able to get mtDNA sequences from the river buffalo genomes as they average coverage is pretty good. Possibly I misread the supplementary tables but some of samples of river buffalo, for examples, from India and Murrah have coverage between 9x and ~20x (from supplementary table 1).<br>Response: We really thank the reviewer for this advice. In previous analyses, we mapped the sequencing reads only on the swamp mitochondrial reference sequence (NC_006295.1). The results showed so many 'N' in the assembled river sequences that we didn't perform further analyses using the reconstructed river mtDNAs. Now, we mapped the sequencing reads of river buffaloes on the specific river mitochondrial genome (AF547270.1). Therefore, we were able to obtain high quality riverine mitochondrial genomes (Line 160-163) that allowed us to construct the phylogenetic tree (Supplementary figure 7) and network (Figure 2).<br><br>Minor points:<br>Supplementary tables: please report the tag "river" and "swamp" in all tables to make it easier to understand which samples belong to which categories instead of cross check multiple tables every time.<br>Response: We have revised, please check.<br>Line 88: I would suggest the author to change the word remainder referring to Indonesia as not appropriate.<br>Response: We have changed "the remainder of Indonesia" to "the rest of Indonesia", please check (Line 89).<br>Supplementary Table 3 is mentioned for the first time after Supplementary Table 4.<br>Response: We have now resolved this inconsistency, please check.
**Additional Information:**	
**Question**	**Response**
Are you submitting this manuscript to a special series or article collection?	No
**Experimental design and statistics**<br><br>Full details of the experimental design and statistical methods used should be given in the Methods section, as detailed in our Minimum Standards Reporting Checklist. Information essential to interpreting the data presented should be made available in the figure legends.<br><br>Have you included all the information requested in your manuscript?	Yes
**Resources**<br><br>A description of all resources used, including antibodies, cell lines, animals and software tools, with enough information to allow them to be uniquely identified, should be included in the Methods section. Authors are strongly encouraged to cite Research Resource Identifiers (RRIDs) for antibodies, model	Yes

organisms and tools, where possible.<br><br>Have you included the information requested as detailed in our Minimum Standards Reporting Checklist?	
**Availability of data and materials**<br><br>All datasets and code on which the conclusions of the paper rely must be either included in your submission or deposited in publicly available repositories (where available and ethically appropriate), referencing such data using a unique identifier in the references and in the “Availability of Data and Materials” section of your manuscript.<br><br>Have you have met the above requirement as detailed in our Minimum Standards Reporting Checklist?	No
If not, please give reasons for any omissions below.<br><br>as follow-up to "**Availability of data and materials**<br><br>All datasets and code on which the conclusions of the paper rely must be either included in your submission or deposited in publicly available repositories (where available and ethically appropriate), referencing such data using a unique identifier in the references and in the “Availability of Data and Materials” section of your manuscript.<br><br>Have you have met the above requirement as detailed in our Minimum Standards Reporting Checklist?<br><br>"	The 98 whole genomes resequecing data will be submit to the NCBI, and the accession number will be provided soon.

Manuscript
Click here to access/download;Manuscript;GIGA-D-19-00183-R1-authors.docx
Click here to view linked References

Genomic Analyses Reveal Distinct Genetic Architectures and Selective Pressures in Buffaloes

Ting Sun[1#], Jiafei Shen[1#], Alessandro Achilli[7#], Ningbo Chen[1], Qiuming Chen[1], Ruihua Dang[1], Zhuqing Zheng[1], Hucai Zhang[2], Xiaoming Zhang[3], Shaoqiang Wang[1], Tao Zhang[4], Hongzhao Lu[4], Yun Ma[5], Yutang Jia[6], Marco Rosario Capodiferro[7], Yongzhen Huang[1], Xianyong Lan[1], Hong Chen[1], Yu Jiang[1*], Chuzhao Lei[1*]

[1]Key Laboratory of Animal Genetics, Breeding and Reproduction of Shaanxi Province, College of Animal Science and Technology, Northwest A&F University, Yangling, Shaanxi 712100, China.

[2]Key Laboratory of Plateau Lake Ecology and Environment Change, Yunnan University, Kunming 650504, China.

[3]State Key Laboratory of Genetic Resources and Evolution, Kunming Institute of Zoology, Chinese Academy of Sciences, Kunming 650223, China.

[4]School of Bioscience and Engineering, Shaanxi University of Technology, Hanzhong, Shaanxi 723000, China.

[5]Agricultural College, Ningxia University, Yinchuan 750021, China

[6]Institute of Animal Science and Veterinary Medicine, Anhui Academy of Agriculture Science, Hefei 230001, China.

[7]Dipartimento di Biologia e Biotecnologie "L. Spallanzani", Università di Pavia, Pavia, 27100, Italy.

e-mail addresses:

Ting Sun: sunting_sim07@163.com

Jiafei Shen: shenjiafei0118@163.com

Ningbo Chen: ningboch@126.com

Qiuming Chen: cqm19860612@126.com

Ruihua Dang: dangruihua@nwsuaf.edu.cn

Zhuqing Zheng: zzq1207@126.com

Hucai Zhang: hucaizhang@yahoo.com

Xiaoming Zhang: zhangxiaoming@mail.kiz.ac.cn

Shaoqiang Wang: oracle_2@163.com

Tao Zhang: zl780823@163.com

Hongzhao Lu:zl780823@126.com

Yun Ma: mayun666@yahoo.com.cn

Yutang Jia: yutang2018@163.com

Marco Rosario Capodiferro: marcorosario.capodiferro01@universitadipavia.it

Yongzhen Huang: huangyongzhen126@126.com

Xianyong Lan: lanxianyong79@nwafu.edu.cn

Hong Chen: chenhong1212@126.com

Alessandro Achilli: alessandro.achilli@unipv.it

Yu Jiang: yu.jiang@nwafu.edu.cn

Chuzhao Lei: leichuzhao1118@nwafu.edu.cn

[#]These authors contributed equally to this work.

*Corresponding author. E-mail: yu.jiang@nwafu.edu.cn (Y.J.) and leichuzhao1118@126.com (C.L.).

ORCIDs:

Alessandro Achilli, 0000-0001-6871-3451;

Yu Jiang, 0000-0003-4821-3585;

Chuzhao Lei, 0000-0003-1647-1037.

Abstract

Background

The domestic buffalo (*Bubalus bubalis*) is an essential farm animal in tropical and subtropical regions, whose genomic diversity is yet to be fully discovered.

Results

In this study, we describe the demographic events and selective pressures of buffalo by analyzing 121 whole genomes (98 newly reported) from 25 swamp and river buffalo breeds. Both uniparental and biparental markers were investigated to provide the final scenario. The ancestors of swamp and river buffalo diverged ~0.23 Mya and then experienced independent demographic histories. They were domesticated in different regions, the swamp buffalo at the border between Southwest China and Southeast Asia, while the river buffalo in South Asia. The domestic stocks migrated to other regions and further differentiated, as testified by (at least) two ancestral components identified in each subspecies. Different signals of selective pressures were also detected in these two types of buffalo. The swamp buffalo, historically used as a draft animal, shows selection signatures in genes associated with nervous system, while in river dairy breeds, genes under selection are related to heat-stress and immunity.

Conclusions

Our findings substantially expand the catalogue of genetic variants in buffalo, reveal new insights into the evolutionary history and distinct selective pressures in river and swamp buffalo.

Key words: buffalo; whole-genome resequencing; genetic history; selection

Background

The domestic buffalo is an important farm animal in tropical and subtropical regions, which can provide milk, meat, and draught power for the rice cultivation. The domestic buffalo can be divided into two types: swamp and river buffalo. These two types show differences concerning body size, outward appearance, biological characteristics and chromosome karyotype (2n = 48 in swamp buffalo; 2n = 50 in river buffalo) [1, 2]. The swamp buffalo is mainly bred in extensive rural areas in Northeast India, Southeast Asia and South China, while the river buffalo is distributed from Western India to Mediterranean areas. Swamp buffalo was traditionally raised as a draught animal for rice cultivation, while the river type was mainly selected for milk production [3].

There is a large agreement on the common ancestor of river and swamp buffaloes, both descending from the wild Asian buffalo (*Bubalus arnee*) [3]. However, details on the domestication process and its consequences are still missing. The oldest domestic buffalo remains of Southeast Asia were found in Northern Thailand and dated to 2,900-2,300 years ago [4], while other archeozoological evidence is scarce [5]. Therefore, the genomic screening of current breeds was often employed to clarify the overall scenario. In particular, both uniparental markers were initially studied [6-13], while further details were eventually provided by autosomal analyses [14]. The current data on river buffalo point to an initial domestication in the Indian subcontinent 6,300-4,600 YBP and a following migration westwards into Southern Europe [6, 10, 14]. The swamp buffalo was probably domesticated in the China/Indochina border. Then, the original stocks migrated to other regions: northward to China and then bending southwards into the Philippines; southward initially across the Mekong, then to Sumatra (Mekong colonization) and finally eastwards to the rest of Indonesia [12-14].

Considering that the entire genome variation of buffalo was largely unexplored [15], we sequenced the whole genome of 98 buffaloes from 21 swamp and four river breeds (Supplementary tables 1 and 2) with different geographic origins in order to fully describe the genomic diversity, population structure, and demographic history of this important livestock species and to reveal possible signs of natural and artificial selection.

Data Description

We sampled a total of 98 buffaloes (NCBI:txid89462) from different locations: China (81), Laos (5), Vietnam (4) and India (one Nili-Ravi, two Murrah, and five Indian buffaloes). All genomic data have been submitted to the NCBI Short Read Archive under the BioProject accession number PRJNA547460. After adding 23 available genomes (figure 1a, Supplementary tables 1 and 2) [15], the final dataset of 121 genomes was subdivided into six geographic groups: Upper Yangtze, Middle-Lower Yangtze, Southwest China, Southeast Asia, South Asia and Italy (figure 1a, Supplementary figure 1).

Analyses

Population genetic structure and relationships

Neighbour-joining (NJ) trees, principal component analysis (PCA) and ADMIXTURE were used to explore the genetic relationships among the examined 121 buffaloes. The NJ tree, rooted with *Syncerus caffer*, showed a deep division between swamp and river buffaloes, then buffaloes from adjacent geographical regions form distinctive clades (figure 1b). The same geographic/genomic proximity was also confirmed by the maximum-likelihood (ML) tree (Supplementary figure 2). The PCA (figure 1c and 1d) showed that the first principal component (PC1) was driven by differences between swamp and river buffaloes. The PC2 (figure 1c) separates Italian and South Asian river buffaloes. This structure was also confirmed (Supplementary Note2, Supplementary figure 3) when whole genome data were merged with samples genotyped using the 90K Axiom^{TM} Buffalo Genotyping Array [14]. The PC3 (figure 1d, Supplementary figure 4, Supplementary table 7) highlights the variability among swamp breeds separating the Southwest Chinese and Vietnamese buffaloes from other swamp breeds. The ADMIXTURE analysis confirmed this genetic structure (figure 1e, Supplementary table 8, Supplementary figure 5). At K = 4, all individuals were unambiguously assigned to two ancestries in swamp buffalo (cold colors: South China, SC; Southeast Asia, SEA) and two in river buffalo (warm colors: South Asia, SA; Italy, ITA) (figure 1e). Within swamp buffalo, the SC ancestral component (blue) characterizes most of Upper and Middle-Lower Yangtze Valley

buffaloes, but it was also detected in Southwest Chinese and Laotian (LA) breeds. The SEA ancestry (green) was shared between Vietnamese and three Southwest Chinese breeds, and found at low level in Middle-Lower Yangtze showing evidence of recent admixture, probably due to the frequent trading of buffaloes among these regions. As for the river buffalo, the SA ancestry (orange) was abundant in Murrah and Nili-Ravi buffaloes, while the ITA ancestry (red) is unique to Italian breeds. Some swamp buffaloes showed evidence of admixture, which may be attributable to introgression events by way of recent crossbreeding with river buffalo for improving milk production traits [16].

Uniparental phylogenies

Y-chromosome and mitochondrial DNA (mtDNA) are very useful to investigate genetic origins and ancient migrations (Supplementary Note 3). After quality control and filtering, 520 Y-chromosome SNPs were retrieved from 89 male buffaloes and used to build a phylogenetic tree that clearly divides swamp (YS) and river (YR) clades. Most of the variants defined the branch that connects swamp and river common ancestors. Two haplogroups (YS1 and YS2) were identified in the swamp branch, both retrieved in all geographic regions. The haplogroup YS1 dominated the buffaloes from Upper and Middle-Lower Yangtze (76.09%), while the haplogroup YS2 was extremely frequent (84.62%) in Southwest China and Southeast Asia (figure 2a, Supplementary figure 6, and Supplementary table 9).

We also inferred the maternal history of buffalo combining the novel 118 mitogenomes from this study with 107 sequences from previous studies (Supplementary table 10) [12]. Swamp buffaloes can be assigned to five previously defined lineages: two major haplogroups (SA and SB with various sub-clades) and three rare ones (SC, SD, and SE) (figure 2b, Supplementary figure 7, Supplementary table 10). This larger dataset confirmed the geographic differentiation of current swamp buffalo populations (figure 1e), as previously reported by analyzing partial and complete mtDNA data [12, 13]. The Upper and Middle-Lower Yangtze buffalo breeds primarily belonged to lineage SA1. Buffaloes from Southwest China and Southeast Asia almost harbor all lineages, except for the rare lineage SE, showed high

frequencies of SA2 and SB2. The highest variety of lineages was identified in Southwest China and Southeast Asia, thus confirming the hypothesized domestication of swamp buffalo at the border of the two regions [12, 14].

As for the Y-chromosome variation of river buffalo, we identified one ancestral node (YR) and two haplogroups (YR1 and YR2). The YR haplotype was found in one Indian, two south Asian and nine Southern Chinese buffaloes. The latter finding was probably due to recent importation of bulls in China through the cross-breeding programs [16], consistently with the autosomal analyses (PCA, ADMIXTURE and NJ tree, figure 1b-1e). The haplogroups YR1 and YR2 were found in South Asia and Italy, respectively (figure 2a, 2b, and Supplementary figure 4). Four different mtDNA haplogroups have been identified in river buffaloes (figure 2b), thus adding a new lineage (R4) to the three previously defined ones (R1, R2, and R3). However, considering le low number (and coverage) of river mitogenomes (22 Italian and 10 South Asian buffaloes) no further phylogeographic analyses have been carried out (Figure 2).

Demographic history

We employed the multiple sequentially Markovian coalescent (MSMC) method to detect the changes in the effective population size (N_e) of four "ancestral" buffalo groups. River and swamp buffaloes underwent two apparent expansions and two bottlenecks that seemed to overlap with three major glacial cycles (figure 3a). Initially, the N_e of river and swamp buffaloes showed similar demographic trajectories with a peak at ~0.8 Mya and then quickly declining during the Naynayxungla glaciation (NG, 0.78-0.50 Mya), which was the most extensive glaciation during the Quaternary Period. The ancestral N_e of river buffalo recovered very quickly and reached the highest peak at ~70 Kya after a short bottleneck ~0.23 Mya. On the contrary, the ancestors of the swamp buffalo suffered a long period of population decline until the retreat of the Penultimate glaciation (PG, ~0.30-0.13 Mya), and then, the N_e slightly increased starting from ~0.10 Mya. During the interglacial period, both river and swamp buffalo population reached another peak and quickly declined during the last glaciation (LG). These results confirmed that the glaciations had a strong effect on the demographic history of swamp

buffalo, as already observed analyzing complete mtDNAs [12]. The decline from ~6.0 to ~4.5 Kya is consistent with the onset of domestication, before the final increase until present time.

The MSMC approach was also used to calculate the divergence time among four buffalo ancestral populations: SC, SEA, SA and ITA (figure 3b). We observed a decrease in the cross-coalescence rate between river and swamp buffaloes to 0.5 approximately 0.21~0.23 Mya (0.25 at approximately at 0.15 ~ 0.18 Mya; to 0.75 at approximately 0.28~0.38 Mya). The splitting time of SC and SA ancestors was observed ~28 Kya, while a decline to 0.5 between ITA and SA was detected later ~11 Kya.

Genome-wide differential selection in river and swamp buffalo

We applied four methods (F_{ST}, π ln ratio, XP-CLR, XP-EHH) to detect genomic regions related to selection in river and swamp buffalo. Two or more methods showed outlier signals (*p-value* < 0.005) in overlapping regions and were therefore considered as candidate selective regions. Functional gene-set enrichment was used to identify Kyoto Encyclopedia of Genes and Genomes (KEGG) pathways and Gene Ontology (GO) terms that are statistically significant associated with the genes.

In river buffalo, a total of 502 candidate regions under selection containing 569 genes were detected (Supplementary table 11-14). Candidate genes in river buffalo are significantly over-represented (corrected *p-value* < 0.05) in Jak-STAT signaling pathway, Glioma, and pathways associated with cancer (Supplementary table 15-16). The Jak-STAT pathway plays a crucial role in prolactin signal transduction of mammary gland [17] and control of immune responses [18, 19]. We also identified GO terms associated with immunity and other with DNA damage and repair (Supplementary table 16, Supplementary figure 8a). In particular, four candidate genes (*AP4B1*, *BCL2L15*, *PHTF1*, and *PTPN22*) (figure 4a) are associated with the immune system response, while *MMS22L* (figure 4c) may be associated with heat stress. Moreover, we detected few non-synonymous variants that are completely fixed at *PTPN22* and *BCL2L15* in river buffaloes (figure 4b, Supplementary figure 8b), as well as one at *MMS22L* (figure 4d). Additional signs of selection were identified in genes coding for productive and economically

significant traits, such as *NUMB* [20] and *SGMS2* [21] associated with milk production, while others related to growth (*NRF1*) [22], and feed efficiency (*TNPO3*) [23].

In swamp buffalo, a total of 171 candidate regions under selection containing 209 genes were detected (Supplementary tables 11, and 17-19). These genes can be significantly associated (corrected *p-value* < 0.05) to four KEGG pathways (Supplementary tables 20-21). The most significant one was "Glutamatergic synapse" involving five genes (*HOMER1*, *GRIK2*, *DLGAP1*, *GNG7*, *LOC102398542*) (figure 5, Supplementary tables 20, Supplementary figure 9). We also found significantly over-represented GO terms associated with dendritic spines (*TIAM1*, *RELN*, *DISC1*, *NLGN1*, *LOC102398542*) and nervous system (neuron, dendritic spine, synapse, etc.) involving 42 genes (*HDAC9*, *HOMER1*, *BIN1*, and *GRIK2* showed higher values in the detected methods) (Supplementary table 21, Supplementary figure 9). Among the over- these detected candidate genes, *HDAC9*, *HOMER1*, and *GRIK2* (figure 5a, b and c) may be associated with the development of the nervous system in swamp buffalo (figure 5d).

Discussion

In this study, we analyzed the whole genome sequence of 121 buffaloes (91 swamp and 30 river buffaloes). Our autosomal data reveal an ancient separation between river and swamp buffaloes ~0.23 Mya, overlapping with previously reported data [9, 11, 12] and certainly predating buffalo domestication. Therefore, we can assume that river and swamp buffaloes probably descended from different wild populations. The demographic histories of swamp and river buffaloes were differentially linked to climatic changes. A similar pattern was observed in taurine and indicine cattle, suggesting similar habitat requirements [24]. After diverging, the two types of buffalo evolved independently. In fact, two different ancestral components were identified for each of them (figure 1e). Distinctive Y-chromosome lineages were also revealed in river buffalo, with a basal haplogroup (YR) unique to breeds from India and Pakistan, YR1 typical of South Asia and YR2 identified only in Italy. The most likely scenario based on previous studies [14] points to an early river buffalo domestication in the Indo-Pakistan region, then migrating westward. Later, YR1 remained in South Asia, where is still highly diffused,

whereas YR2 became unique to the buffaloes bred in Italy. In swamp buffalo, YS2 is found mostly in Southwest China and Southeast Asia (84.62%), while YS1 dominates buffaloes from Upper and Middle-Lower Yangtze (76.09%). Considering that YS2 diverge earlier in the phylogeny, we might speculate that swamp buffalo population migrated from the southern regions towards the north, where the YS1 experienced a population expansion. A clear geographic pattern was also identified in the mitochondrial gene pool of swamp buffalo. Taking into account the frequencies of uniparental haplogroups in swamp buffalo (figure 2), we could observe a correlation between Y-chromosome and mtDNA haplogroups (i.e YS1 with SA and YS2 with SB) which could mark some similarities between maternal and paternal histories.

We identified significant and distinctive signatures of selective sweeps in these two types buffalo. In order to better understand possible explanations for the selective pressures, we have explored the most likely biological functions of these genes. River buffaloes are mainly distributed in Western India to Mediterranean areas, where domestic herds are usually more resistant to various diseases than in tropical regions [16]. In river genomes, positively selected genes are significantly over-represented in GO terms associated with immunity. Among these, *PTPN22* encodes a negative regulator of T-cell receptor (TCR), which was associated with human autoimmune diseases [25-28], bovine leukemia virus [29], and milk somatic cell counts of cow [29]. Among the other genes (*PTPN22, AP4B1, BCL2L15,* and *PHTF1*) were related to bovine leukemia virus [30] and human autoimmune diseases [28]. The *BCL2L15* SNP A226G (acid changed: T76A) is a conserved region and almost fixed with the allele A in river buffalo (P > 0.90) (Supplementary figure 8b). In addition, heat stress is a significant issue for many livestock species, particularly for dairy animals, leading to different problems in the phenotypic features (e.g. impairment of reproduction and slower growth), as well as in the cellular stability, causing a reduced efficiency of the DNA synthesis [31]. We found three GO terms associated with DNA damage and repair (cellular response to DNA damage stimulus; DNA repair; double-strand break repair) that are significantly over-represented in river buffalo. *MMS22L*, a component of the *MMS22L-TONSL* complex important for the DNA repair system [32, 33], was involved in these three GO terms. Actually, heat stress can induce the formation of double-

stranded DNA break (DSB) [34] and inhibit the functionality of the homologous recombination system [35]. DSB can be repaired by homologous recombination (HR), allowing DNA replication to continue at stalled or broken forks [36, 37]. *MMS22L* can facilitate HR-mediated maintenance of genome stability during DNA replication [38]. Taking into account that river buffalo is mainly selected for milk production and well adapted to hot climate [39], these gene might play an important role in heat adaptability of river buffalo. Finally, we were able to identify signatures of selection also on some genes for important economic and reproductive traits, which is not unexpected considering the great effort undertaken by the herders to improve the river breeds.

Swamp buffalo is historically used as a draft animal to provide farm power in rice cultivation. Therefore, these animals are very docile and easy to handle and train. The swamp buffalo genome showed signs of selection in some genes of the "Glutamatergic synapse" pathway (*HOMER1*, *GRIK2*, *DLGAP1*, *GNG7*, and *LOC102398542*), which plays an important role in the behavior, particularly concerning adaptation to stress and fear responses (Supplementary table 20) [40]. We also found over-represented genes in GO categories associated with nervous system involving 42 genes. Among them *HDAC9*, *HOMER1*, *BIN1*, and *GRIK2* seem the best candidates we have identified (Supplementary table 21). *HDAC9* (figure 5a), a member of class II HDAC proteins, plays a crucial role in neuronal differentiation during cortical development [41] and muscle development [42-45]. A study showed that degradation of class II HDAC proteins can activate myocyte enhancer factor 2, which enhances muscle endurance and fatigue resistance [46]. *HOMER1* (figure 5b), encodes a member of the homer family of dendritic proteins, involving in the several psychiatric disorders, such as schizophrenia [47, 48], major depression [49]. *HOMER1* plays an important role in brain development and behavior; *HOMER1* knockout mice showed deficits in learning and memorizing, and impairment of pain perception [47, 50-53]. *HOMER1* is also an important scaffold for TRP channels and regulates mechanotransduction in skeletal muscle [54]. Mice lacking *HOMER1* showed myopathy with decreased muscle fiber cross-sectional area and reduced skeletal muscle strength generation [54]. Studies have showed that *BIN1* is associated

with the Alzheimer Disease [55-57]. *BIN1* is also involved in the biogenesis of T-tubules, which are responsible for the plasma membrane invaginations that allow for the excitation-contraction coupling machinery in cardiac and skeletal muscles [58-60]. *GRIK2* (figure 5c, 5d), encodes for GluR6, a kainite receptor which is highly expressed in the brain and is associated with autosomal recessive mental retardation [61]. The *GRIK2* knockout mice exhibited reduction in fear memory [62], less anxiety and more risk-taking type than despair-type behavior [63]. Notably, *GRIK2* was also identified as a candidate selective gene in domestic rabbits [64]. In addition, we also identified over-represented GO categories associated with dendritic spines (*TIAM1*, *RELN*, *DISC1*, *NLGN1*, *LOC102398542*) (Supplementary figure 9). The structural and functional plasticity of dendritic spines is crucial for learning and memorizing [65]. *TIAM1* plays an important role in the formation and morphogenesis of dendritic spines [66-68]. *RELN*, *DISC1*, *NLGN1* were related to schizophrenia, mood disorders, memory [69-77]. Therefor these genes could be associated with the nervous system development in swamp buffalo.

Potential implications

This is the first population genetics study on buffalo using a large amount of whole-genome resequencing data. We reconstructed the genetic history and population structure of buffalo from all genetic perspectives using both uniparental and biparental markers. The final scenario indicates that the ancestors of swamp and river buffalo diverged about 0.23 Mya. The swamp was then domesticated at the border between Southwest China to Southeast Asia, while the river in South Asia (between Northern India and Pakistan). The domestic herds then migrated to other regions and further differentiated. In fact, we were able to identify two ancestral and distinctive components in the current genomes of swamp (South China and Southeast Asia components) and river (South Asia and Italy) populations. The different genetic history of these two subspecies is also evident by two distinct selection patterns identified in their genomes. River buffalo was selected to improve milk production, while the swamp buffalo was mainly raised to provide power for the rice cultivation. We were able to intercept distinctive signature of selection in genes associated with nervous and muscle development in swamp buffaloes and

in genes related to economic and reproductive traits in river breeds.

In summary, this is the first study providing a large amount of genomic data on river and swamp buffaloes, which was needed to describe their current genetic diversities and population structures, to reconstruct their demographic histories and to scan for distinctive selective pressures.

Methods

Sample Collection and Sequencing

We sampled a total of 98 buffaloes from different locations: China (81), Laos (5), Vietnam (4) and India (one Nili-Ravi, two Murrah, and five Indian buffaloes). Genomic DNA was extracted from ear tissue or blood samples using the standard phenol-chloroform protocol [78], amplified in genomic libraries with an average insert size of 500 bp, and sequenced (150-bp paired-end reads) on an Illumina HiSeq 2000. We also considered 23 available genome sequences from river buffalo, including 22 Mediterranean and one Murrah buffaloes. Additional details are provided in Supplementary tables 1 and 2. This study was approved by Institutional Animal Care and Use Committee of Northwest A&F University (Permit number: NWAFAC1019).

Alignments and Variant Identification

All cleaned reads were aligned to the reference genome (GCA_000471725.1) linked to "24+X+unplaced" pseudo-chromosomes (Supplementary Note 1, Supplementary table 4) using BWA-MEM with default settings [79]. Duplicate reads were filtered using Picard tools. The single nucleotide polymorphisms (SNPs) were detected with the Genome Analysis Toolkit (GATK, version 3.6-0-g89b7209) [80] and filtered using the "VariantFiltration" tool, as described in Supplementary Note 1.

Phylogenetic and Population Structure Analyses

The neighbour-joining (NJ) tree, principal component analysis (PCA), and ADMIXTURE methods were used to explore the genetic relationships among buffalo populations

(Supplementary Note 2). An individual-based NJ tree based on the matrix of pairwise genetic distances from the autosomal SNP data of 121 buffaloes was constructed with PLINK (version 1.9; PLINK, RRID:SCR_001757) and visualized with FigTree (FigTree, RRID:SCR_008515). TreeMix program was used to construct a population-level phylogeny [81]. The principal component analysis (PCA) was performed using SmartPCA program in the package EIGENSOFT v5.0 (Eigensoft, RRID:SCR_004965) [82] and eigenvectors' significance was detected by the Tracy-Widom test. The population genetic structure was estimated using ADMIXTURE v. 1.3.0 (ADMIXTURE, RRID:SCR_001263) [83] considering from 2 to 5 clusters (K).

Y-chromosome and mitogenome phylogenies

After removing sites shared with female buffaloes, heterozygous sites and sites with a genotyping rate <5%, a total of 520 male-specific SNPs were used to construct the phylogenetic tree with BEAST 1.8.0 (BEAST, RRID:SCR_010228) (Supplementary Note 3). A total of 98 mitochondrial genomes with an average coverage > 100× were assembled from the whole-genome resequencing data. Additional 107 whole mtDNA sequences were obtained from GenBank. A phylogenetic tree based on the final alignment was constructed using RaxML (RAxML, RRID:SCR_006086) with the following parameters: -f a -x 123 -p 23 -# 100 -k -m 132 GTRGAMMA. The phylogenies were built using pegas [84].

Estimates of the effective population size and divergence time

A multiple sequential coalescent Markovian model (MSMC) was used to infer effective population sizes (N_e) and divergence times considering two samples with average coverage > 16× for each population. Autosomal SNPs of each sample were identified using GATK (GATK, RRID:SCR_001876). After removing variant outliers (with extremely low or high coverage), all sites were phased using BEAGLE v. 4.1 (BEAGLE, RRID:SCR_001789) [85]. The same high-coverage samples were also used to infer relative cross-coalescence rate (RCCR), considering a value of 0.5 as a reference to extrapolate split times between populations

(samples). The time scale is calculated using an average generation time of six years (g=6) and a mutation rate of $\mu_g = 1.26\times10^{-8}$ [86].

Genome-wide selective sweep test

To detect selective sweeps in swamp and river buffalo, we performed the following comparisons: (i) the swamp buffalo as a reference and the river buffalo as the object population; (ii) the river buffalo as a reference and the swamp buffalo as the object population. A total of four methods were used: (i) The fixation index (F_{ST}) values [87] were calculated in sliding 50kb windows with 20kb steps along the autosomes using VCFtools (VCFtools, RRID:SCR_001235) [88]; (ii) high differences in genetic diversity (π ln ratio) were calculated with 50kb sliding-windows and 20kb steps along the autosomes using VCFtools and in-house scripts (iii) The cross-population composite likelihood ratio (XP-CLR) is a likelihood method for detecting selective sweeps that the change in allele frequency at the locus occurred too quickly to be due to random drift between two populations [89]. We used non-overlapping sliding windows of 50kb, maximum number of SNPs within each window as 600, and correlation level from which the SNPs contribution to XP-CLR result was down weighted to 0.95. (iv) We also performed the cross-population extended haplotype homozygosity (XP-EHH) test for every SNP using the default settings of the selscan v1.1 [90], which was designed to detect ongoing or nearly fixed selective sweeps by comparing haplotypes from two populations [91]. For the XP-EHH selection scan, our test statistic was the average normalized XP-EHH score in each 50-kb region. Significant genomic regions were identified by *p-value* < 0.005. Two or more methods showed significant signals (*p-value* < 0.005) in overlapping regions and were therefore considered as the candidate regions affected by selection. The KOBAS 3.0 tool (KOBAS, RRID:SCR_006350) [92]was used to gain a better understanding of their biological functions and involved pathways as enriched GO terms and KEEG pathways.

Availability of supporting data

Raw sequencing data is available from EBI European Nucleotide Archive Bioproject number

PRJNA547460. All supporting genotype data and additional materials are available in the *GigaScience* GigaDB database [93].

Additional files

Supplementary Note 1 Linking pseudo-chromosomes.

Supplementary Note 2 The whole-genome diversity of buffalo.

Supplementary Note 3 Population structure analysis.

Supplementary Note 4 Y-chromosome and Whole mitochondrial genome phylogeny.

Supplementary figure 1. Genome-wide distribution of nucleotide diversity of buffaloes in six geographical regions in 50-kb sliding windows with 20-kb steps.

Supplementary figure 2. TreeMix relationships between 25 buffalo breeds.

Supplementary figure 3. PCA of river buffaloes, with PC1 plotted against PC2.

Supplementary figure 4. PCA of swamp buffalo with PC1 plotted against PC2.

Supplementary figure 5. Model-based clustering of buffalo using the ADMIXTURE program with K from 2 to 5.

Supplementary figure 6. ML phylogeny of the Y-chromosome using 520 SNPs for 89 buffaloes.

Supplementary figure 7. ML phylogeny of the mitochondrial genome.

Supplementary figure 8. (a) Hierarchical graph of the over-represented (with significant corrected p-values < 0.05) GO terms associated with immunity. The color intensity is positively correlated to the corrected p-value of the GO term. (b) Amino acid conservation of *BCL2L15* (first exon) in mammals. Most amino acids are highly conserved with only few exceptions, including SNP A226G (acid changed: T76A).

Supplementary figure 9. (a) Hierarchical graph of the over-represented (with significant corrected p-values < 0.05) GO terms associated with the nervous system. The color intensity is positively correlated to the corrected p-value of the GO term. (b) Amino acid conservation of *LOC102398542*. Nonsynonymous SNP C455T (acid changed: P152L) located in the first exon Amino acids at this site are highly conserved in other mammals.

Supplementary table 1. Overview of sample information and sequencing statistics concerning the 121 buffaloes analyzed in this study.

Supplementary table 2. Summary information on 25 buffalo breeds.

Supplementary table 3. Distribution of SNPs within various genomic regions.

Supplementary table 4. Summary information of the linked pseudo-chromosomes.

Supplementary table 5. The θπ value for the buffalo population groups.

Supplementary table 6. Pairwise F_{ST} values.

Supplementary table 7. Tracy-Widom (TW) statistics and *p-value* for the ten first eigenvalues in the PCA of buffaloes.

Supplementary table 8. Cross-validation (CV) errors for ADMIXTURE ancestry models with K ranging from 2 to 5.

Supplementary Table 9. The genotype of 520 SNPs in the Y chromosome.

Supplementary table 10. Mapping results for 118 novel buffalo mitochondrial genomes plus 107 published mitogenomes.

Supplementary table 11. A summary of genes from F_{ST}.

Supplementary table 12. A summary of genes from XP-CLR (*p-value* < 0.5%) in river buffalo.

Supplementary table 13. A summary of genes from ln ratio(π_{swamp}/π_{river}) (*p-value* < 0.5%) in river buffalo.

Supplementary table 14. A summary of genes from XP-EHH in river buffalo.

Supplementary table 15. KEGG pathway analysis of candidate genes in river buffalo.

Supplementary table 16. Go enrichment of candidate genes in river buffalo.

Supplementary table 17. A summary of genes from XP-CLR (*p-value* < 0.5%) in swamp buffalo.

Supplementary table 18. A summary of genes from ln ratio(π_{river}/π_{swamp}) (*p-value* < 0.5%) in swamp buffalo.

Supplementary table 19. A summary of genes from XP-EHH in swamp buffalo.

Supplementary table 20. KEGG pathway analysis of candidate genes in swamp buffalo.

Supplementary table 21. Go enrichment of candidate genes in swamp buffalo.

Abbreviations

NJ tree: Neighbour-joining tree; PCA: principal component analysis; mtDNA: mitochondrial

DNA; MSMC: multiple sequentially Markovian coalescent; Ne: effective population size; XP-CLR: the cross-population composite likelihood ratio; XP-EHH: the cross-population extended haplotype homozygosity; KEGG: Kyoto Encyclopedia of Genes and Genomes; GO: Gene Ontology.

Competing interests

We declare we have no competing interests.

Funding

The work was supported by the National Beef Cattle and Yak Industrial Technology System (CARS-37), Natural Science Foundation of China (31872317) to Chuzhao Lei, and National Thousand Youth Talents Plan to Yu Jiang; the Italian Ministry of Education, University and Research (MIUR), i.e. Dipartimenti di Eccellenza Program (2018-2022)-Dept. of Biology and Biotechnology "L. Spallanzani," University of Pavia (to A.A.).

Authors' contributions

Y.J. and C.Z.L. conceived and supervised the experiments. T.S., J.F.SH. performed majority of analysis with contributions from Q.M.CH., N.B.CH.,and ZH.Q.ZH. T.S., A. A. wrote and revised the manuscript. R.H.D., H.C.ZH, X.M.ZH, M.R.C., Y.ZH.H., X.Y.L., and H.CH. provided and prepared the samples. All authors reviewed the manuscript and gave final approval for publication.

Acknowledgements

We thank Wen Wang sharing the genome data of *Syncerus caffer*.

Figure legends

Figure 1. Population structure and relationships among buffaloes. (a) Geographic map indicating the origins of the buffalo breeds. (b) Neighbour-joining tree of buffaloes constructed

using whole-genome autosomal SNP data. (c, d) Principal component analyses (PCA) showing PC1 against PC2 and PC1 against PC3, respectively. Each breed was labeled with different colors and shapes as showed in the top of Figure 1e. (e) Genetic structure of buffalo breeds using ADMIXTURE program with K = 2, 4. Population acronyms are explained in Supplementary Tables 1 and 5.

Figure 2. Y-chromosome and mitogenome phylogenies. The width of the edges is proportional to the number of pairwise differences between the joined haplotypes. (a) Y-chromosome network using 520 SNPs. (b) Mitogenome network of swamp buffalo.

Figure 3. Demographic history and divergence of buffalo populations using MSMC. (a) Population size history inference of swamp and river buffalo based on four high-coverage haplotypes from Southwest China (SC), Southeast Asia (SEA), South Asia (SA), and Italy individuals (ITA). (b) Inferred relative cross-coalescence rates between pairs of populations over time based on the same four haplotypes.

Figure 4. Signatures of selective sweep regions at *PTPN22* and *MMSL22* genes in river buffalo. Different parameters were estimated for each gene (*PTPN22* and *MMSL22*)*:* nucleotide diversity, degree of haplotype sharing across populations (a and c). A red arrow notes the specific gene region. A schematic structure of each gene (b and d) is also depicted with exons indicated by vertical bars and reference/alternative alleles noted with different colors (green/yellow) and combined to form different haplotypes (each with a specific haplotype frequency next to it). Non-synonymous SNPs are highlighted in gray.

Figure 5. Signatures of selective sweep regions at *HDAC9*, *HOMER1* and *GRIK2* genes in swamp buffalo. See the legend of Figure 4 for further details.

References

1. Fischer H and Ulbrich F. Chromosomes of the Murrah buffalo and its crossbreds with the Asiatic swamp buffalo (*Bubalus bubalis*). Zeitschrift für Tierzüchtung und Züchtungsbiologie. 1967;84 1 - 4:110-4. doi:10.1111/j.1439-0388.1967.tb01102.x.
2. Iannuzzi L. Standard karyotype of the river buffalo (Bubalus bubalis L., 2n = 50). Report of the committee for the standardization of banded karyotypes of the river buffalo. Cytogenetics and cell genetics. 1994;67 doi:10.1159/000133807.
3. Cockrill WR. The water buffalo: a review. The British veterinary journal. 1981;137 1:8-16. doi:10.1016/S0007-1935(17)31782-7.
4. Pietrusewsky M. The People of Ban Chiang: Bioarchaeology of the 1974 and 1975 Skeletons. In: *The International Conference on the Anniversary of the Discovery of the Ban Chiang Site* 2016.
5. Cluttonbrock J. A natural history of domesticated mammals. Zoologica Africana. 1990;36 1:113-20. doi:10.1080/15627020.2001.11657122.
6. Nagarajan M, Nimisha K and Kumar S. Mitochondrial DNA Variability of Domestic River Buffalo (Bubalus bubalis) Populations: Genetic Evidence for Domestication of River Buffalo in Indian Subcontinent. Genome Biology & Evolution. 2015;7 5:496-503. doi:10.1093/gbe/evv067.
7. Yindee M, Vlamings BH, Wajjwalku W, Techakumphu M, Lohachit C, Sirivaidyapong S, et al. Y-chromosomal variation confirms independent domestications of swamp and river buffalo. Animal Genetics. 2010; doi:10.1111/j.1365-2052.2010.02020.x.
8. Lei CZ, Zhang W, Chen H, Lu F, Ge QL, Liu RY, et al. Two Maternal Lineages Revealed by Mitochondrial DNA D-loop Sequences in Chinese Native Water Buffaloes (Bubalus bubalis). Asian Australasian Journal of Animal Sciences. 2007;20 4:471-6.
9. Lei CZ, Zhang W, Chen H, Lu F, Liu RY, Yang XY, et al. Independent maternal origin of Chinese swamp buffalo (*Bubalus bubalis*). Animal Genetics. 2007;38 2:97-102. doi:10.1111/j.1365-2052.2007.01567.x.
10. Kumar S, Nagarajan M, Sandhu JS, Kumar N and Behl V. Phylogeography and domestication of Indian river buffalo. BMC Evolutionary Biology. 2007;7 1:186. doi:10.1186/1471-2148-7-186.
11. Kumar S, Nagarajan M, Sandhu J, Kumar N, Behl V and Nishanth G. Mitochondrial DNA analyses of Indian water buffalo support a distinct genetic origin of river and swamp buffalo. Animal genetics. 2007;38 3:227-32. doi:10.1111/j.1365-2052.2007.01602.x.
12. Wang S, Chen N, Capodiferro MR, Zhang T, Lancioni H, Zhang H, et al. Whole Mitogenomes Reveal the History of Swamp Buffalo: Initially Shaped by Glacial Periods and Eventually Modelled by Domestication. Scientific Reports. 2017;7 1:4708. doi:10.1038/s41598-017-04830-2.
13. Zhang Y, Lu Y, Yindee M, Li K-Y, Kuo H-Y, Ju Y-T, et al. Strong and stable geographic differentiation of swamp buffalo maternal and paternal lineages indicates domestication in the China/Indochina border region. Molecular Ecology. 2016;25 7:1530-50.

doi:doi:10.1111/mec.13518.

14. Colli L, Milanesi M, Vajana E, Iamartino D, Bomba L, Puglisi F, et al. New Insights on Water Buffalo Genomic Diversity and Post-Domestication Migration Routes From Medium Density SNP Chip Data. Frontiers in Genetics. 2018;9 53 doi:10.3389/fgene.2018.00053.

15. Whitacre LK, Hoff JL, Schnabel RD, Albarella S, Ciotola F, Peretti V, et al. Elucidating the genetic basis of an oligogenic birth defect using whole genome sequence data in a non-model organism, Bubalus bubalis. Scientific Reports. 2017;7:39719. doi:10.1038/srep39719.

16. Borghese A. Buffalo production and research. Italian Journal of Animal Science. 2005;5 2.

17. Watson CJ and Burdon TG. Prolactin signal transduction mechanisms in the mammary gland: the role of the Jak/Stat pathway. Reviews of reproduction. 1996;1 1:1-5.

18. Shuai K and Liu B. Regulation of JAK-STAT signalling in the immune system. Nature reviews Immunology. 2003;3 11:900-11. doi:10.1038/nri1226.

19. O'Shea John J and Plenge R. JAK and STAT Signaling Molecules in Immunoregulation and Immune-Mediated Disease. Immunity. 2012;36 4:542-50. doi:10.1016/j.immuni.2012.03.014.

20. Liu L-L, Fang C and Liu W-J. Identification on novel locus of dairy traits of Kazakh horse in Xinjiang. Gene. 2018;677:105-10. doi:10.1016/j.gene.2018.07.009.

21. Li H, Wang Z, Moore SS, Schenkel FS and Stothard P. Genome-wide Scan For Positional And Functional Candidate Genes Affecting Milk Production Traits In Canadian Holstein Cattle. 2010.

22. Wei X, Li H, Yang J, Hao D, Dong D, Huang Y, et al. Circular RNA profiling reveals an abundant circLMO7 that regulates myoblasts differentiation and survival by sponging miR-378a-3p. Cell Death &Amp; Disease. 2017;8:e3153. doi:10.1038/cddis.2017.541.

23. Hardie LC, Vandehaar MJ, Tempelman RJ, Weigel KA, Armentano LE, Wiggans GR, et al. The genetic and biological basis of feed efficiency in mid-lactation Holstein dairy cows. Journal of Dairy Science. 2017;100 11 doi:10.3168/jds.2017-12604.

24. Mei C, Wang H, Liao Q, Wang L, Cheng G, Wang H, et al. Genetic Architecture and Selection of Chinese Cattle Revealed by Whole Genome Resequencing. Molecular Biology and Evolution. 2018;35 3:688-99. doi:10.1093/molbev/msx322.

25. Begovich AB, Carlton VEH, Honigberg LA, Schrodi SJ, Chokkalingam AP, Alexander HC, et al. A missense single-nucleotide polymorphism in a gene encoding a protein tyrosine phosphatase (PTPN22) is associated with rheumatoid arthritis. American journal of human genetics. 2004;75 2:330-7.

26. Bottini N, Musumeci L, Alonso A, Rahmouni S, Nika K, Rostamkhani M, et al. A functional variant of lymphoid tyrosine phosphatase is associated with type I diabetes. Nature Genetics. 2004;36:337.

27. Kyogoku C, Langefeld CD, Ortmann WA, Lee A, Selby S, Carlton VEH, et al. Genetic association of the R620W polymorphism of protein tyrosine phosphatase PTPN22 with

human SLE. American journal of human genetics. 2004;75 3:504-7.
28. Ban Y, Tozaki T and Nakano Y. Association Studies of the GPR103 and BCL2L15 Genes in Autoimmune Thyroid Disease in the Japanese Population. Frontiers in Endocrinology. 2016;7 92 doi:10.3389/fendo.2016.00092.
29. Ibeagha-Awemu EM, Peters SO, Akwanji KA, Imumorin IG and Zhao X. High density genome wide genotyping-by-sequencing and association identifies common and low frequency SNPs, and novel candidate genes influencing cow milk traits. Scientific Reports. 2016;6:31109. doi:10.1038/srep31109.
30. Brym P, Bojarojćnosowicz B, Oleński K, Hering DM, Ruść A, Kaczmarczyk E, et al. Genome-wide association study for host response to bovine leukemia virus in Holstein cows. Vet Immunol Immunopathol. 2016;175:24-35.
31. Belhadj Slimen I, Najar T, Ghram A and Abdrrabba M. Heat stress effects on livestock: molecular, cellular and metabolic aspects, a review. Journal of Animal Physiology and Animal Nutrition. 2016;100 3:401-12. doi:10.1111/jpn.12379.
32. Saredi G, Huang H, Hammond CM, Alabert C, Bekker-Jensen S, Forne I, et al. H4K20me0 marks post-replicative chromatin and recruits the TONSL–MMS22L DNA repair complex. Nature. 2016;534:714. doi:10.1038/nature18312.
33. Ben-Aroya S, Agmon N, Yuen K, Kwok T, McManus K, Kupiec M, et al. Proteasome Nuclear Activity Affects Chromosome Stability by Controlling the Turnover of Mms22, a Protein Important for DNA Repair. PLOS Genetics. 2010;6 2:e1000852. doi:10.1371/journal.pgen.1000852.
34. George I, Wenqi W and Minli W. DNA double strand break repair inhibition as a cause of heat radiosensitization: re-evaluation considering backup pathways of NHEJ. International Journal of Hyperthermia the Official Journal of European Society for Hyperthermic Oncology North American Hyperthermia Group. 2008;24 1:17. doi:10.1080/02656730701784782.
35. Kantidze OL, Velichko AK, Luzhin AV and Razin SV. Heat Stress-Induced DNA Damage. Acta naturae. 2016;8 2:75-8.
36. Branzei D and Foiani M. Maintaining genome stability at the replication fork. Nature Reviews Molecular Cell Biology. 2010;11:208. doi:10.1038/nrm2852.
37. Filippo JS, Sung P and Klein H. Mechanism of Eukaryotic Homologous Recombination. Annual Review of Biochemistry. 2008;77 1:229-57. doi:10.1146/annurev.biochem.77.061306.125255.
38. Duro E, Lundin C, Ask K, Sanchez-Pulido L, MacArtney TJ, Toth R, et al. Identification of the MMS22L-TONSL Complex that Promotes Homologous Recombination. Molecular Cell. 2010;40 4:632-44. doi:10.1016/j.molcel.2010.10.023.
39. Marai IFM and Haeeb AAM. Buffalo's biological functions as affected by heat stress-A review. Livestock Science. 2010;127 2:89-109. doi:10.1016/j.livsci.2009.08.001.
40. Kamprath K, Plendl W, Marsicano G, Deussing JM, Wurst W, Lutz B, et al. Endocannabinoids mediate acute fear adaptation via glutamatergic neurons independently of corticotropin-releasing hormone signaling. Genes, Brain and Behavior. 2009;8 2:203-11. doi:10.1111/j.1601-183X.2008.00463.x.

41. Sugo N, Oshiro H, Takemura M, Kobayashi T, Kohno Y, Uesaka N, et al. Nucleocytoplasmic translocation of HDAC9 regulates gene expression and dendritic growth in developing cortical neurons. European Journal of Neuroscience. 2010;31 9:1521-32. doi:10.1111/j.1460-9568.2010.07218.x.
42. Zhang S, Xu H, Liu X, Yang Q, Pan C, Lei C, et al. The muscle development transcriptome landscape of ovariectomized goat. Royal Society Open Science. 2017;4 12:171415. doi:10.1098/rsos.171415.
43. Haberland M, Arnold MA, McAnally J, Phan D, Kim Y and Olson EN. Regulation of HDAC9 Gene Expression by MEF2 Establishes a Negative-Feedback Loop in the Transcriptional Circuitry of Muscle Differentiation. Molecular and Cellular Biology. 2007;27 2:518. doi:10.1098/rsos.171415.
44. Mei C, Wang H, Liao Q, Khan R, Raza SHA, Zhao C, et al. Genome-wide analysis reveals the effects of artificial selection on production and meat quality traits in Qinchuan cattle. Genomics. 2018; doi:10.1016/j.ygeno.2018.09.021.
45. Haberland M, Montgomery RL and Olson EN. The many roles of histone deacetylases in development and physiology: implications for disease and therapy. Nature Reviews Genetics. 2009;10:32. doi:10.1038/nrg2485.
46. Potthoff MJ, Wu H, Arnold MA, Shelton JM, Backs J, McAnally J, et al. Histone deacetylase degradation andMEF2 activation promote the formation of slow-twitch myofibers. The Journal of Clinical Investigation. 2007;117 9:2459-67. doi:10.1172/JCI31960.
47. Szumlinski KK, Lominac KD, Kleschen MJ, Oleson EB, Dehoff MH, Schwartz MK, et al. Behavioral and neurochemical phenotyping of Homer1 mutant mice: possible relevance to schizophrenia. Genes, Brain and Behavior. 2005;4 5:273-88. doi:10.1111/j.1601-183X.2005.00120.x.
48. Spellmann I, Rujescu D, Musil R, Mayr A, Giegling I, Genius J, et al. Homer-1 polymorphisms are associated with psychopathology and response to treatment in schizophrenic patients. Journal of Psychiatric Research. 2011;45 2:234-41. doi:10.1016/j.jpsychires.2010.06.004.
49. Rietschel M, Mattheisen M, Frank J, Treutlein J, Degenhardt F, Breuer R, et al. Genome-Wide Association-, Replication-, and Neuroimaging Study Implicates HOMER1 in the Etiology of Major Depression. Biological Psychiatry. 2010;68 6:578-85. doi:10.1016/j.biopsych.2010.05.038.
50. Jaubert PJ, Golub MS, Lo YY, Germann SL, Dehoff MH, Worley PF, et al. Complex, multimodal behavioral profile of the Homer1 knockout mouse. Genes, Brain and Behavior. 2007;6 2:141-54. doi:10.1111/j.1601-183X.2006.00240.x.
51. Gerstein H, O'Riordan K, Osting S, Schwarz M and Burger C. Rescue of synaptic plasticity and spatial learning deficits in the hippocampus of Homer1 knockout mice by recombinant Adeno-associated viral gene delivery of Homer1c. Neurobiology of Learning and Memory. 2012;97 1:17-29. doi:10.1016/j.nlm.2011.08.009.
52. Inoue N, Nakao H, Migishima R, Hino T, Matsui M, Hayashi F, et al. Requirement of the immediate early gene vesl-1S/homer-1a for fear memory formation. Molecular

Brain. 2009;2 1:7. doi:10.1186/1756-6606-2-7.
53. Klugmann M and Szumlinski KK. Targeting Homer genes using adeno-associated viral vector: lessons learned from behavioural and neurochemical studies. Behavioural pharmacology. 2008;19 5-6:485-500. doi:10.1097/FBP.0b013e32830c369f.
54. Stiber JA, Zhang Z-S, Burch J, Eu JP, Zhang S, Truskey GA, et al. Mice Lacking Homer 1 Exhibit a Skeletal Myopathy Characterized by Abnormal Transient Receptor Potential Channel Activity. Molecular and Cellular Biology. 2008;28 8:2637-47. doi:10.1128/MCB.01601-07.
55. Chapuis J, Hansmannel F, Gistelinck M, Mounier A, Van Cauwenberghe C, Kolen KV, et al. Increased expression of BIN1 mediates Alzheimer genetic risk by modulating tau pathology. Molecular Psychiatry. 2013;18:1225. doi:10.1038/mp.2013.1.
56. Wijsman EM, Pankratz ND, Choi Y, Rothstein JH, Faber KM, Cheng R, et al. Genome-Wide Association of Familial Late-Onset Alzheimer's Disease Replicates BIN1 and CLU and Nominates CUGBP2 in Interaction with APOE. PLOS Genetics. 2011;7 2:e1001308. doi:10.1371/journal.pgen.1001308.
57. Yu L, Chibnik LB, Srivastava GP, Pochet N, Yang J, Xu J, et al. Association of Brain DNA Methylation in SORL1, ABCA7, HLA-DRB5, SLC24A4, and BIN1 With Pathological Diagnosis of Alzheimer DiseaseBrain DNA Methylation and Pathological AD DiagnosisBrain DNA Methylation and Pathological AD Diagnosis. JAMA Neurology. 2015;72 1:15-24. doi:10.1001/jamaneurol.2014.3049.
58. Lee E, Marcucci M, Daniell L, Pypaert M, Weisz OA, Ochoa G-C, et al. Amphiphysin 2 (Bin1) and T-Tubule Biogenesis in Muscle. Science. 2002;297 5584:1193. doi:10.1126/science.1071362.
59. Razzaq A, Robinson IM, McMahon HT, Skepper JN, Su Y, Zelhof AC, et al. Amphiphysin is necessary for organization of the excitation-contraction coupling machinery of muscles, but not for synaptic vesicle endocytosis in Drosophila. Genes & development. 2001;15 22:2967-79. doi:10.1101/gad.207801.
60. Butler MH, David C, Ochoa G-C, Freyberg Z, Daniell L, Grabs D, et al. Amphiphysin II (SH3P9; BIN1), a Member of the Amphiphysin/Rvs Family, Is Concentrated in the Cortical Cytomatrix of Axon Initial Segments and Nodes of Ranvier in Brain and around T Tubules in Skeletal Muscle. The Journal of Cell Biology. 1997;137 6:1355. doi:10.1083/jcb.137.6.1355.
61. Motazacker MM, Rost BR, Hucho T, Garshasbi M, Kahrizi K, Ullmann R, et al. A Defect in the Ionotropic Glutamate Receptor 6 Gene (GRIK2) Is Associated with Autosomal Recessive Mental Retardation. The American Journal of Human Genetics. 2007;81 4:792-8. doi:doi.org/10.1086/521275.
62. Ko S, Zhao M-G, Toyoda H, Qiu C-S and Zhuo M. Altered Behavioral Responses to Noxious Stimuli and Fear in Glutamate Receptor 5 (GluR5)- or GluR6-Deficient Mice. The Journal of Neuroscience. 2005;25 4:977. doi:10.1523/JNEUROSCI.4059-04.2005.
63. Shaltiel G, Maeng S, Malkesman O, Pearson B, Schloesser RJ, Tragon T, et al. Evidence for the involvement of the kainate receptor subunit GluR6 (GRIK2) in mediating behavioral displays related to behavioral symptoms of mania. Molecular Psychiatry.

2008;13:858. doi:10.1038/mp.2008.20.

64. Carneiro M, Rubin C-J, Di Palma F, Albert FW, Alföldi J, Barrio AM, et al. Rabbit genome analysis reveals a polygenic basis for phenotypic change during domestication. Science. 2014;345 6200:1074. doi:10.1126/science.1253714.

65. Kasai H, Matsuzaki M, Noguchi J, Yasumatsu N and Nakahara H. Structure–stability–function relationships of dendritic spines. Trends in Neurosciences. 2003;26 7:360-8. doi:10.1016/S0166-2236(03)00162-0.

66. Zhang H and Macara IG. The polarity protein PAR-3 and TIAM1 cooperate in dendritic spine morphogenesis. Nature Cell Biology. 2006;8 3:227-37. doi:10.1038/ncb1368.

67. Tolias KF, Bikoff JB, Kane CG, Tolias CS, Hu L and Greenberg ME. The Rac1 guanine nucleotide exchange factor Tiam1 mediates EphB receptor-dependent dendritic spine development. Proceedings of the National Academy of Sciences. 2007;104 17:7265. doi:10.1073/pnas.0702044104.

68. Tolias KF, Bikoff JB, Burette A, Paradis S, Harrar D, Tavazoie S, et al. The Rac1-GEF Tiam1 Couples the NMDA Receptor to the Activity-Dependent Development of Dendritic Arbors and Spines. Neuron. 2005;45 4:525-38. doi:10.1016/j.neuron.2005.01.024.

69. Li M, Luo X-J, Xiao X, Shi L, Liu X-Y, Yin L-D, et al. Analysis of common genetic variants identifies RELN as a risk gene for schizophrenia in Chinese population. The World Journal of Biological Psychiatry. 2013;14 2:91-9. doi:10.3109/15622975.2011.587891.

70. Abdolmaleky HM, Cheng K-h, Russo A, Smith CL, Faraone SV, Wilcox M, et al. Hypermethylation of the reelin (RELN) promoter in the brain of schizophrenic patients: A preliminary report. American Journal of Medical Genetics Part B: Neuropsychiatric Genetics. 2005;134B 1:60-6. doi:10.1002/ajmg.b.30140.

71. Zhou Z, Hu Z, Zhang L, Hu Z, Liu H, Liu Z, et al. Identification of RELN variation p.Thr3192Ser in a Chinese family with schizophrenia. Scientific Reports. 2016;6:24327. doi:10.1038/srep24327.

72. Hennah W, Thomson P, Peltonen L and Porteous D. Genes and Schizophrenia: Beyond Schizophrenia: The Role of DISC1 in Major Mental Illness. Schizophrenia Bulletin. 2006;32 3:409-16. doi:10.1093/schbul/sbj079.

73. Mackie S, Millar JK and Porteous DJ. Role of DISC1 in neural development and schizophrenia. Current Opinion in Neurobiology. 2007;17 1:95-102. doi:10.1016/j.conb.2007.01.007.

74. Kamiya A, Kubo K-i, Tomoda T, Takaki M, Youn R, Ozeki Y, et al. A schizophrenia-associated mutation of DISC1 perturbs cerebral cortex development. Nature Cell Biology. 2005;7 12:1167-78. doi:10.1038/ncb1328.

75. Katzman A and Alberini CM. NLGN1 and NLGN2 in the prefrontal cortex: their role in memory consolidation and strengthening. Current Opinion in Neurobiology. 2018;48:122-30. doi:10.1016/j.conb.2017.12.003.

76. Balan S, Yamada K, Hattori E, Iwayama Y, Toyota T, Ohnishi T, et al. Population-Specific Haplotype Association of the Postsynaptic Density Gene DLG4 with

Schizophrenia, in Family-Based Association Studies. PLOS ONE. 2013;8 7:e70302. doi:10.1371/journal.pone.0070302.

77. Cheng M-C, Lu C-L, Luu S-U, Tsai H-M, Hsu S-H, Chen T-T, et al. Genetic and Functional Analysis of the DLG4 Gene Encoding the Post-Synaptic Density Protein 95 in Schizophrenia. PLOS ONE. 2010;5 12:e15107. doi:10.1371/journal.pone.0015107.

78. Green MR and Sambrook J. Molecular Cloning: A Laboratory Manual (Fourth Edition): Three-Volume Set. Cold Spring Harbor Laboratory Pr. 2012.

79. Li H and Durbin R. Fast and accurate short read alignment with Burrows–Wheeler transform. Bioinformatics. 2009;25 14:1754-60. doi:10.1093/bioinformatics/btp324.

80. Nekrutenko A and Taylor J. Next-generation sequencing data interpretation: enhancing reproducibility and accessibility. Nature Reviews Genetics. 2012;13 9:667-72. doi:10.1038/nrg3305.

81. Pickrell JK and Pritchard JK. Inference of Population Splits and Mixtures from Genome-Wide Allele Frequency Data. PLOS Genetics. 2012;8 11:e1002967. doi:10.1371/journal.pgen.1002967.

82. Patterson N, Price AL and Reich D. Population Structure and Eigenanalysis. PLOS Genetics. 2006;2 12:e190. doi:10.1371/journal.pgen.0020190.

83. Alexander DH, Novembre J and Lange K. Fast model-based estimation of ancestry in unrelated individuals. Genome Research. 2009;19 9:1655-64. doi:10.1101/gr.094052.109.

84. Paradis E. pegas: an R package for population genetics with an integrated–modular approach. Bioinformatics. 2010;26 3:419-20. doi:10.1093/bioinformatics/btp696.

85. Browning SR and Browning BL. Rapid and Accurate Haplotype Phasing and Missing-Data Inference for Whole-Genome Association Studies By Use of Localized Haplotype Clustering. American Journal of Human Genetics. 2007;81 5:1084-97. doi:10.1086/521987.

86. Chen N, Cai Y, Chen Q, Li R, Wang K, Huang Y, et al. Whole-genome resequencing reveals world-wide ancestry and adaptive introgression events of domesticated cattle in East Asia. Nature Communications. 2018;9 1:2337. doi:10.1038/s41467-018-04737-0.

87. Weir BS and Cockerham CC. Estimating F-statistics for the analysis of population-structure. Evolution. 1984;38 6:1358-70. doi:10.2307/2408641.

88. Danecek P, Auton A, Abecasis G, Albers CA, Banks E, DePristo MA, et al. The variant call format and VCFtools. Bioinformatics. 2011;27 15:2156-8. doi:10.1093/bioinformatics/btr330.

89. Chen H, Patterson N and Reich D. Population differentiation as a test for selective sweeps. Genome Research. 2010;20 3:393-402.

90. Szpiech ZA and Hernandez RD. selscan: An Efficient Multithreaded Program to Perform EHH-Based Scans for Positive Selection. Molecular Biology and Evolution. 2014;31 10:2824-7. doi:10.1093/molbev/msu211.

91. Sabeti PC, Varilly P, Fry B, Lohmueller J, Hostetter E, Cotsapas C, et al. Genome-wide detection and characterization of positive selection in human populations. Nature. 2007;449:913. doi:10.1038/nature06250.

92. Xie C, Mao X, Huang J, Ding Y, Wu J, Dong S, et al. KOBAS 2.0: a web server for annotation and identification of enriched pathways and diseases. Nucleic Acids Research. 2011;39 suppl_2:W316-W22. doi:10.1093/nar/gkr483.
93. Sun T; Shen J; Chen N; Chen Q; Dang R; Zheng Z; Zhang H; Zhang X; Wang S; Zhang T; Lu H; Ma Y; Jia Y; Capodiferro MR; Huang Y; Lan X; Chen H; Achilli A; Jiang Y; Lei C (2019): Supporting data for "Genomic Analyses Reveal Distinct Genetic Architectures and Selective Pressures in Buffaloes" *GigaScience* Database. http://dx.doi.org/10.5524/100682.

figure1

Click here to access/download;Figure;figure 1.tif

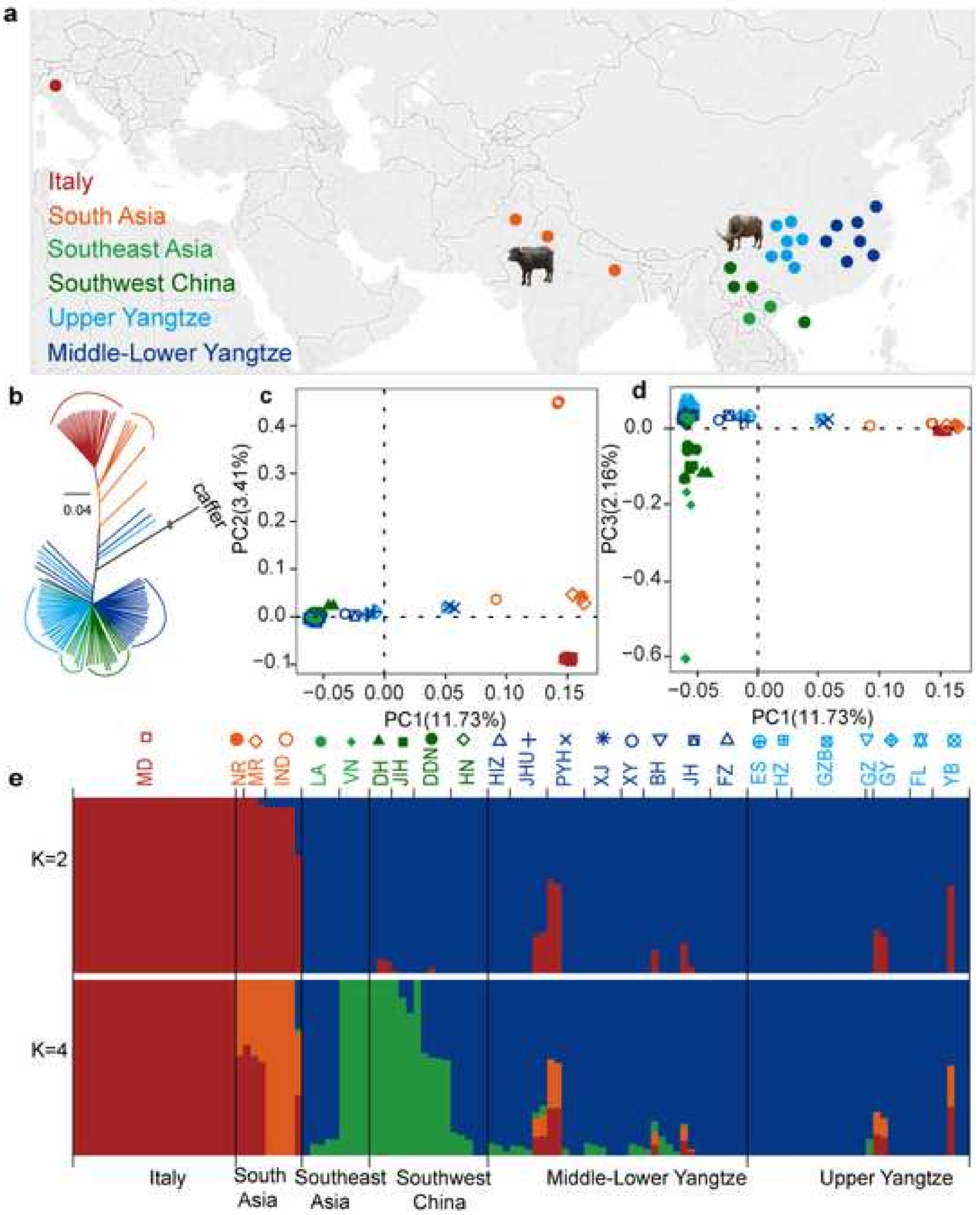

figure 2

Click here to access/download;Figure;figure 2.tif

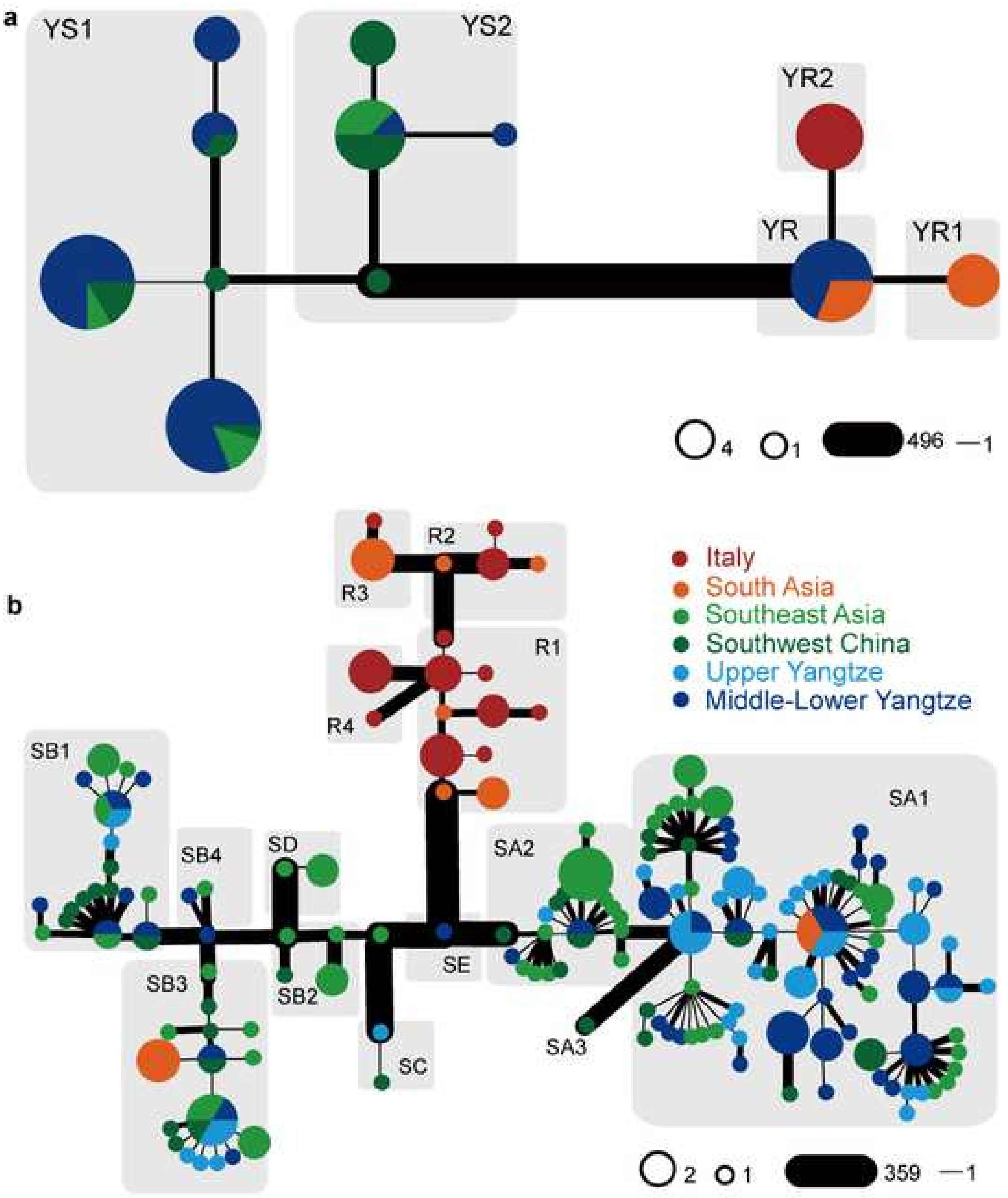

figure 3

Click here to access/download;Figure;figure 3.tif

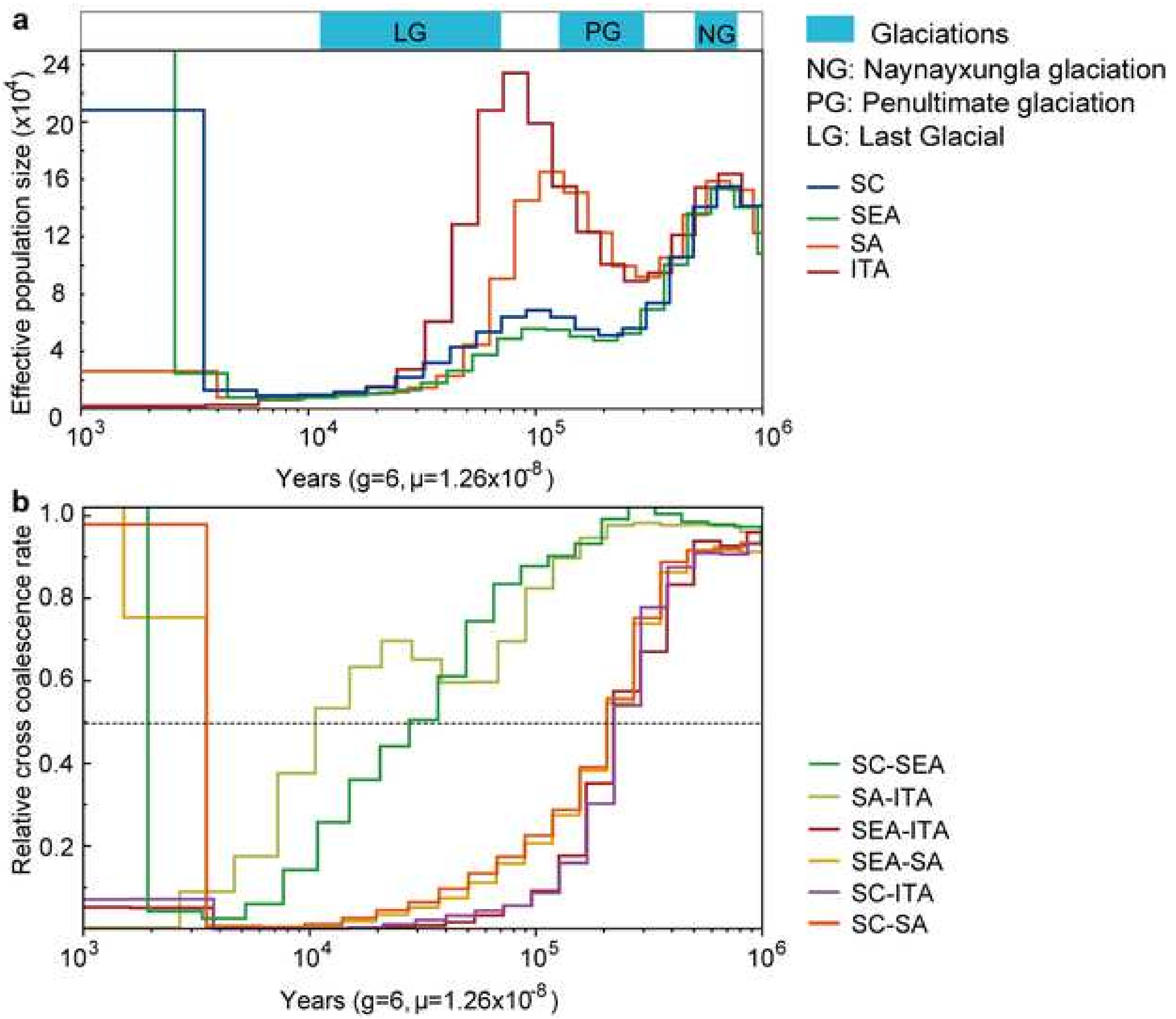

figure 4

Click here to access/download;Figure;figure4.tif

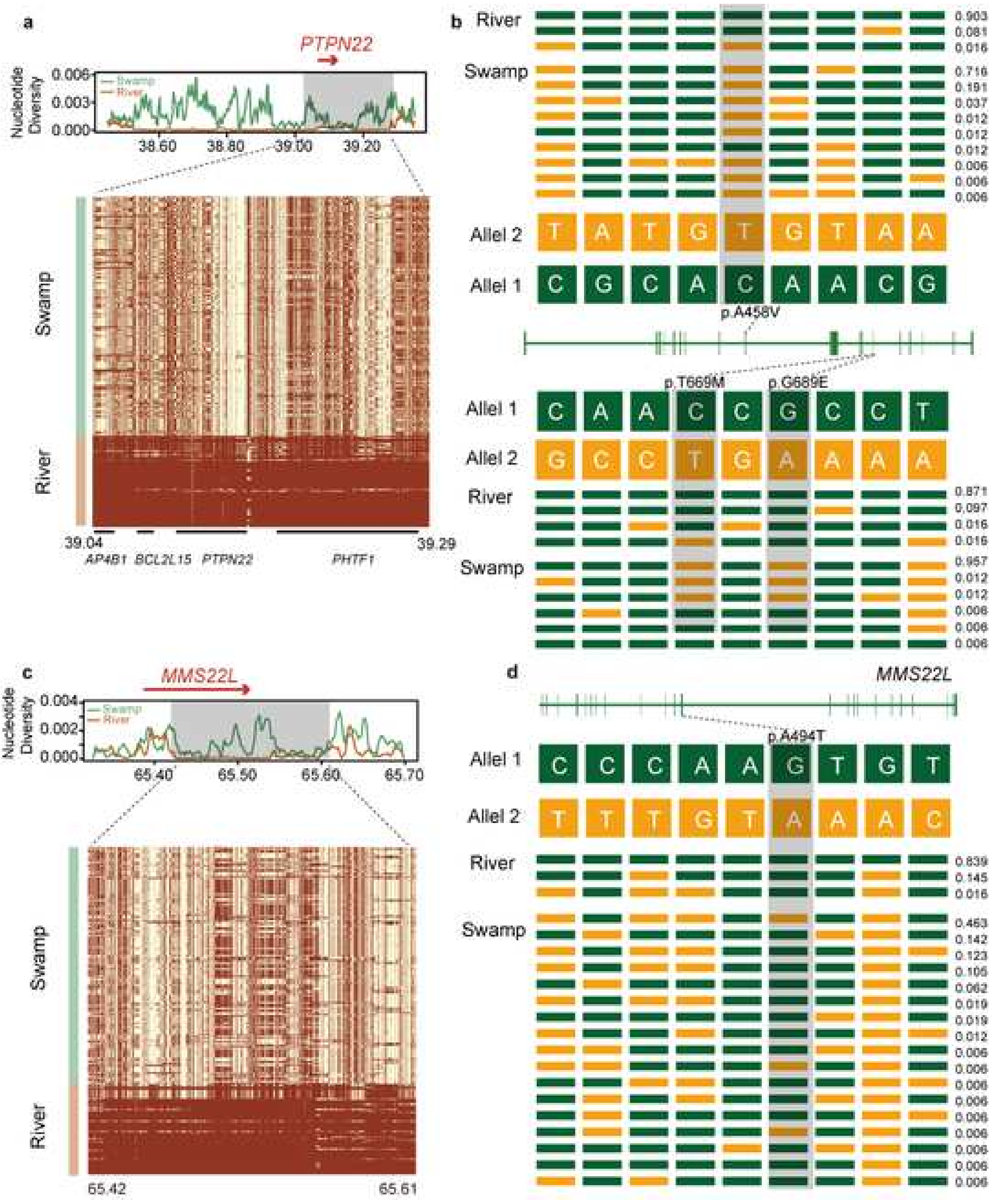

figure 5

Click here to access/download;Figure;figure5.tif

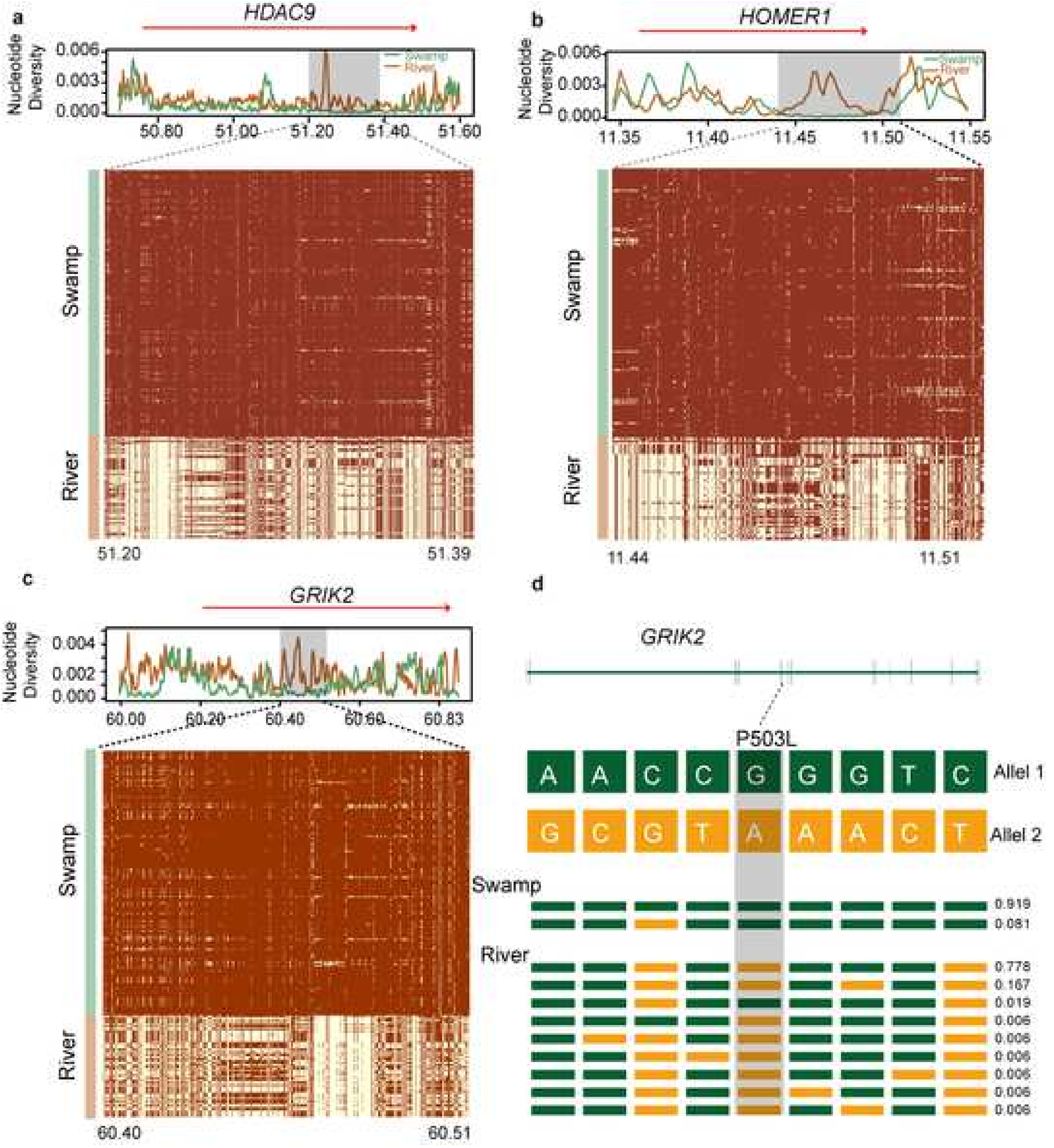

Click here to access/download
Supplementary Material
Supplementary Materials-GIGA-D-19-00183-R1.docx

Click here to access/download
Supplementary Material
Supplementary Tables S9-21-GIGA-D-19-00183-R1.xlsx
