# Supplementary material for: Genomic analyses reveal distinct genetic architectures and selective pressures in buffaloes
Source: Gigascience. 2020 Feb 21;9(2):giz166. doi: 10.1093/gigascience/giz166 (PMC7033652; doi:10.1093/gigascience/giz166)
Supplement: giz166_Supplemental_Files [file giz166_supplemental_files.zip › Supplementary Materials-GIGA-D-19-00183-R1.docx]

**Genomic Analyses Reveal the Genetic Architecture and Selection in Buffalo**

**Sun *et al.***

**Supplementary Information**

|  | **Supplementary Notes** |  | **Page** |  |
| --- | --- | --- | --- | --- |
|  | Supplementary Note 1 |  | 2-3 |  |
|  | Supplementary Note 2 |  | 3 |  |
|  | Supplementary Note 3 |  | 3-4 |  |
|  | Supplementary Note 4 |  | 4-5 |  |
|  | **Supplementary figures** |  | **Page** |  |
|  | Supplementary figure 1 |  | 6 |  |
|  | Supplementary figure 2 |  | 7 |  |
|  | Supplementary figure 3 |  | 8 |  |
|  | Supplementary figure 4 |  | 9 |  |
|  | Supplementary figure 5 |  | 10 |  |
|  | Supplementary figure 6 |  | 11 |  |
|  | Supplementary figure 7 |  | 12 |  |
|  | Supplementary figure 8 |  | 13 |  |
|  | Supplementary figure 9 |  | 14 |  |
|  |  |  |  |  |
|  | **Supplementary tables** |  | **Page** |  |
|  | Supplementary table 1 |  | 15-16 |  |
|  | Supplementary table 2 |  | 17 |  |
|  | Supplementary table 3 |  | 18 |  |
|  | Supplementary table 4 |  | 19 |  |
|  | Supplementary table 5 |  | 20 |  |
|  | Supplementary table 6 |  | 21 |  |
|  | Supplementary table 7 |  | 22 |  |
|  | Supplementary table 8 |  | 23 |  |
|  | **Supplementary Reference** |  | 23-24 |  |
|  |  |  |  |  |
|  |  |  |  |  |

**Supplementary Note 1**

**Linking pseudo-chromosomes**

We linked the scaffold-based water buffalo genome assembly deposited in NCBI (GCA_000471725.1) into “24 + X + unplaced” pseudo-chromosomes (Supplementary table 4). Because of the 500bp-size used for building the libraries, the scaffolds smaller than 400bp were discarded. Scaffolds were cut into 10,000 bps and then these fragments were aligned to the cattle genome sequence (GCF_000003205.7) using BLASR (Basic Local Alignment with Successive Refinement). If one scaffold aligned to more than one chromosome, the location of maximum coverage was used as the location of the scaffold. According to the alignment information, the mapped scaffolds were linked to chromosomes, and the scaffold unmapped to the cattle genome was linked to one “unplaced” chromosome. Each acrocentric river buffalo (BBU) chromosomes corresponded to one of the remaining cattle (BTA) chromosomes as follows: BBU1 for BTA1 and BTA27, BBU2 for BTA2 and BTA23, BBU3 for BTA8 and BTA19, BBU4 for BTA5 and BTA28, and BBU5 for BTA16 and BTA29 [[1](#_ENREF_1)]. Two random scaffolds could be derived from different chromosomes or different regions of the same chromosome. Therefore, we filled the gaps between two linked scaffolds with 1,000 bps of ‘N’, a length exceeding the paired-end read length of ~500 bps, which decreased the ability of the paired-end reads to align to different scaffolds.

All clean reads were aligned to the pseudo-chromosomes using BWA-MEM with the default settings [[2](#_ENREF_2)]. Duplicate reads were filtered using Picard tools. Then, we used the GATK (version 3.6-0-g89b7209) to detect SNPs [[3](#_ENREF_3)]. All SNPs were filtered using the “VariantFiltration” tool implemented in GATK with the following standards: (1) variants with quality depth (QD) <2; (2) variants with FS (Phred-scaled *p-value* using Fisher's exact test to detect strand bias) >60; (3) variants with MQRankSum (Z-score from Wilcoxon rank sum test of Alt vs. Ref read mapping qualities) <-12.5; (4) variants with ReadPosRankSum (Z-score from Wilcoxon rank sum test of Alt vs. Ref base qualities) <-8; (5) variants with MQ (Root Mean Square of the mapping quality) <40.0; (6) the mean sequencing depth of variants (including all individuals) <1/3× and >3×; (7) variants with SOR (Symmetric Odds Ratio of 2×2 contingency Table to detect strand bias) >3.0; (8) maximum missing rate <0.1; and (9) each SNP was restrict to the two alleles.

**Supplementary Note 2**

**The whole-genome diversity of buffalo**

Whole-genome sequencing of 98 modern buffaloes from China, Laos, and Vietnam generated a total of 26.59 billion paired-end reads giving an average depth (coverage) of 9.6× and a mean mapping rate of 98.89%. To place these buffaloes in a more widespread phylogeographic context, we combined our data with 23 available genomes (Supplementary tables 1 and 2) [[4](#_ENREF_4)]. The final dataset of 121 genomes was subdivided into six geographic groups: Upper Yangtze, Middle-Lower Yangtze, Southwest China, Southeast Asia, South Asia and Italy.

In total, around 34.4 million putative autosomal single nucleotide variants (SNPs) were identified (3,427,636 with a minor allele frequency (MAF) <1%, 2,643,818 between 1% and 5%, and 28,281,439 > 5%), including 69,396 nonsynonymous and 153,317 synonymous coding single nucleotide polymorphisms. In particular, 25,632,159 were retrieved from swamp and 21,454,466 from river buffaloes (Supplementary table 3).

As for the swamp buffaloes (except for the samples exhibiting admixture with river buffalo), populations from Southwest China and Southeast Asia showed a higher genomic diversity (1.74 × 10^-3^ and 1.66 × 10^-3^, respectively) than those from Upper (1.61 × 10^-3^) and Middle-lower Yangtze (1.65 × 10^-3^) (Supplementary figure 1, Supplementary table 5). The genomic diversity of river buffalo was much higher in South Asia (2.21 × 10^-3^) than in the Mediterranean area (1.59 × 10^-3^). The population-differentiation statistics (*F*_ST_) revealed a deep division between swamp and river buffaloes, but lower genetic differentiation among populations from closer geographical regions (Supplementary table 6).

**Supplementary Note 3**

**Population structure analysis**

SNPs were filtered for the MAF using the parameter “--maf 0.012” of PLINK 1.9 [[5](#_ENREF_5)] to insure at least three alleles were detected in the data set. We also used the option “--indep-pairwise 50 5 0.2” of PLINK to remove one of a pair of SNPs if the linkage disequilibrium (LD) with a squared correlation greater than 0.2, in windows of 50 variants and shifting by 5 variants. The SNPs that pass the pruning were used to perform PCA and ADMIXTURE analysis. The PCA analysis was performed using SmartPCA program in the package EIGENSOFT v5.0 [[6](#_ENREF_6)] . The significance of the eigenvectors was detected by the Tracy-Widom test. Three PCA were built using different datasets: (1) all 121 buffaloes; (2) only swamp buffaloes (Supplementary figure 4); (3) combining our data with 366 samples genotyped with the Axiom® Buffalo Genotyping Array 90K from Affymetrix [[7](#_ENREF_7)]. In the last PCA, the first PC divided the river buffalo into three groups: buffaloes from Italy and Mozambique, buffaloes from Romania, and river buffaloes from other regions (Supplementary figure 3). The low power in differentiating swamp breeds is due to the breeds/species used to develop the Affimetrix array (only river buffalo). These results clearly highlight the relevance of the present work that was able to identify SNPs specific of the swamp buffalo breeds.

The population genetic structure analyses were conducted using ADMIXTURE (version 1.3.0) [[8](#_ENREF_8)] from 2 to 5 postulated ancestral populations (K) For each K 20 runs were carried out and the cross-validation error for each K is reported in the Supplementary table 8. The results were visualized with R.

**NJ tree and maximum likelihood (ML) tree**

To identify closely related individuals, we calculate the matrix of pairwise genetic distances by the PLINK 1.9 using the option “--distance-matrix”. The matrix of pairwise genetic distances was used to construct an individual-based NJ tree as implemented in MEGA v6.0. The NJ tree was rooted by the *Syncerus caffer* [[9](#_ENREF_9)]. The constructed NJ tree was visualized with FigTree (<http://tree.bio.ed.ac.uk/software/figtree/>). A population-level phylogeny was built using the ML approach implemented in TreeMix (version. 1.12) [[10](#_ENREF_10)]. The option “--indep-pairwise 50 5 0.2” of PLINK was used to remove one of a pair of SNPs if the linkage disequilibrium (LD) is greater than 0.2. The option of “-k 1000 -global” of TreeMix was used to generate the ML tree.

**Supplementary Note 4**

**Y-chromosome and Whole mitochondrial genome phylogeny**

**Y-chromosome phylogeny**

Since the water buffalo reference genome was derived from a female individual, in order to retrieve the Y-chromosome reads, we added the cattle Y-chromosome sequence to the pseudo-chromosomes (Supplementary Note1). The sex determination of the buffalo was performed by comparing the genomic coverage on the X chromosome with the genomic coverage on the autosomal chromosomes. GATK was used to detect Y-chromosome SNPs in the male and female populations; the sites shared with the female buffaloes were all removed in order to retain only the variants in the male specific portion of the Y chromosome. We also removed heterozygous sites and sites with missing genotypes (5%). The final SNPs were filtered based on an MAF of 0.006. Then, we used BEAGLE (version 4.1) [[11](#_ENREF_11)] to infer the haplotype phase and impute missing alleles. We also used the 520 SNPs showing polymorphism in buffalo population to build a Minimum Spanning Network using pegas [[12](#_ENREF_12)]. The phylogenetic tree based on the final alignment was constructed using BEAST v2.4.8 program [[13](#_ENREF_13)].

**Whole mitochondrial genome phylogeny**

To assemble complete mitochondrial genomes (mtDNAs), we mapped the sequencing reads of swamp and river buffalo to the respective reference mitogenome (NC_006295.1 for the swamp and AF547270.1 for the river). Considering that the mitochondrial genome is circular, we duplicated the first 30bps at the end of the reference sequences to assure equal coverage. The samples with a depth-of-coverage lower than 100X were removed, generating a total of 118 buffalo mitochondrial genome sequences. Firstly, the BAM alignments were transformed into fastq, and then mapped to the reference mitochondrial genome with Mapping Iterative Assembler V 1.0 (MIA) [[14](#_ENREF_14)] (parameters: -H 1 -F -i -c -r). Additional whole mtDNA sequences corresponding to individuals of known haplogroup affiliation were retrieved from GenBank. ML Phylogenetic trees were created using RaxML (Randomized Axelerated Maximum Likelihood) with the following parameters: -f a -x 123 -p 23 -# 100 -k -m 132 GTRGAMMA. The Network of mitochondrial genomes was constructed using pegas [[12](#_ENREF_12)].

**Supplementary figures**

**
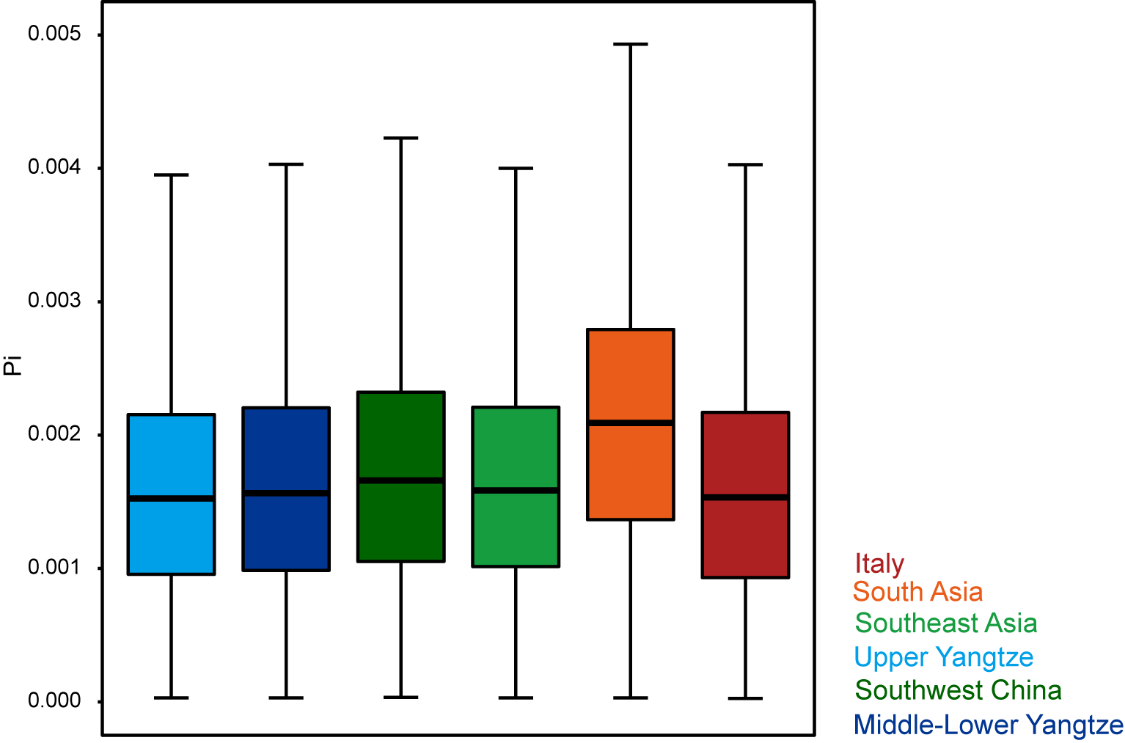
**

**Supplementary figure 1. Genome-wide distribution of nucleotide diversity of buffaloes in six geographical regions in 50-kb sliding windows with 20-kb steps.** The horizontal line inside the box corresponds to the median of each distribution; bottom and top of each box are the first and third quartiles, respectively.


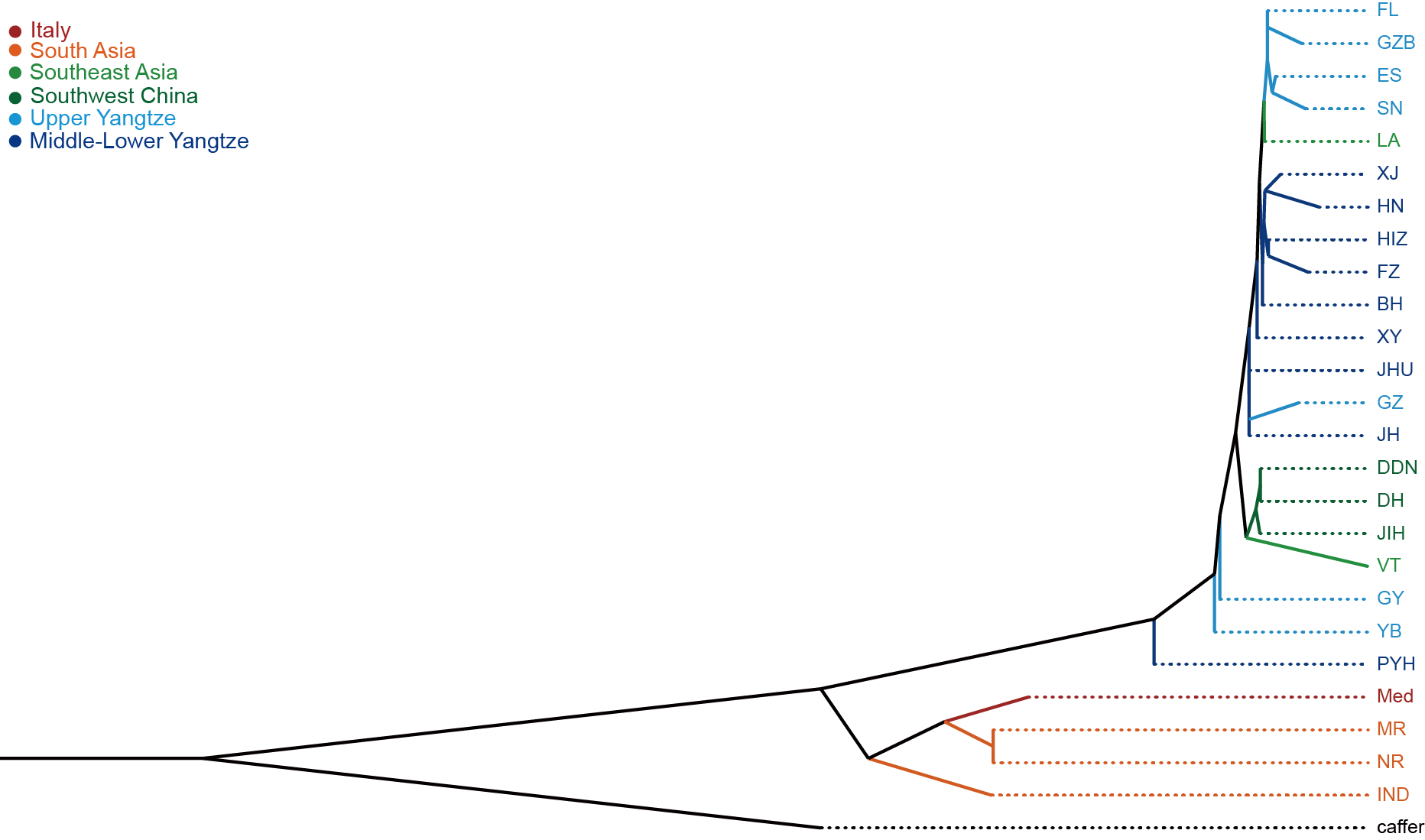


**Supplementary figure 2. TreeMix relationships among the 25 buffalo breeds.** A window size of 1000 was used to account for linkage disequilibrium (-k) and “-global” to generate the ML tree. Each breed was colored according to its geographic group.


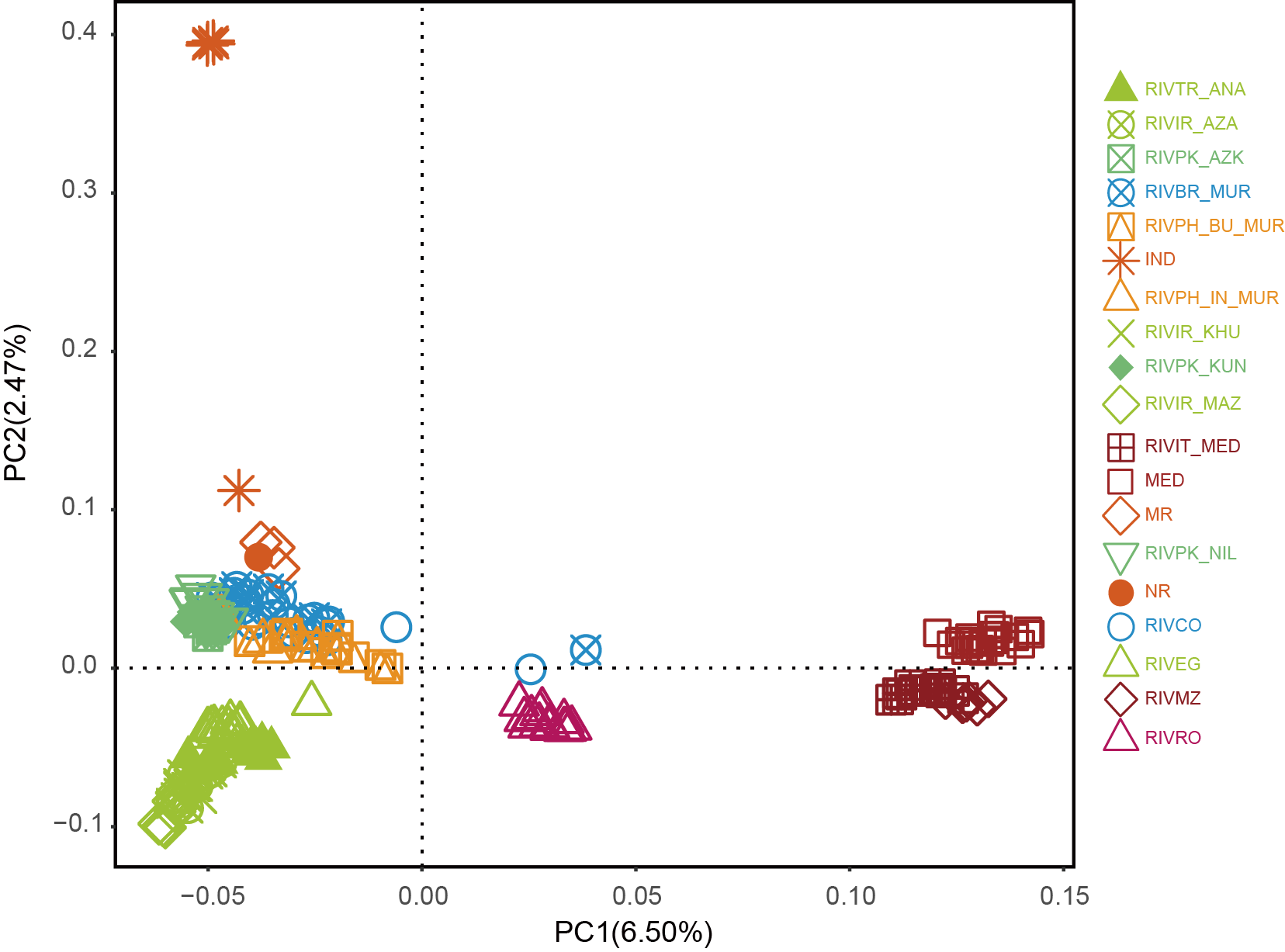


**Supplementary figure 3.** **PCA of river buffaloes, with PC1 plotted against PC2.** The first PC divided the river buffalo into three groups: buffaloes from Italy and Mozambique, buffaloes from Romania, and river buffaloes from other regions.


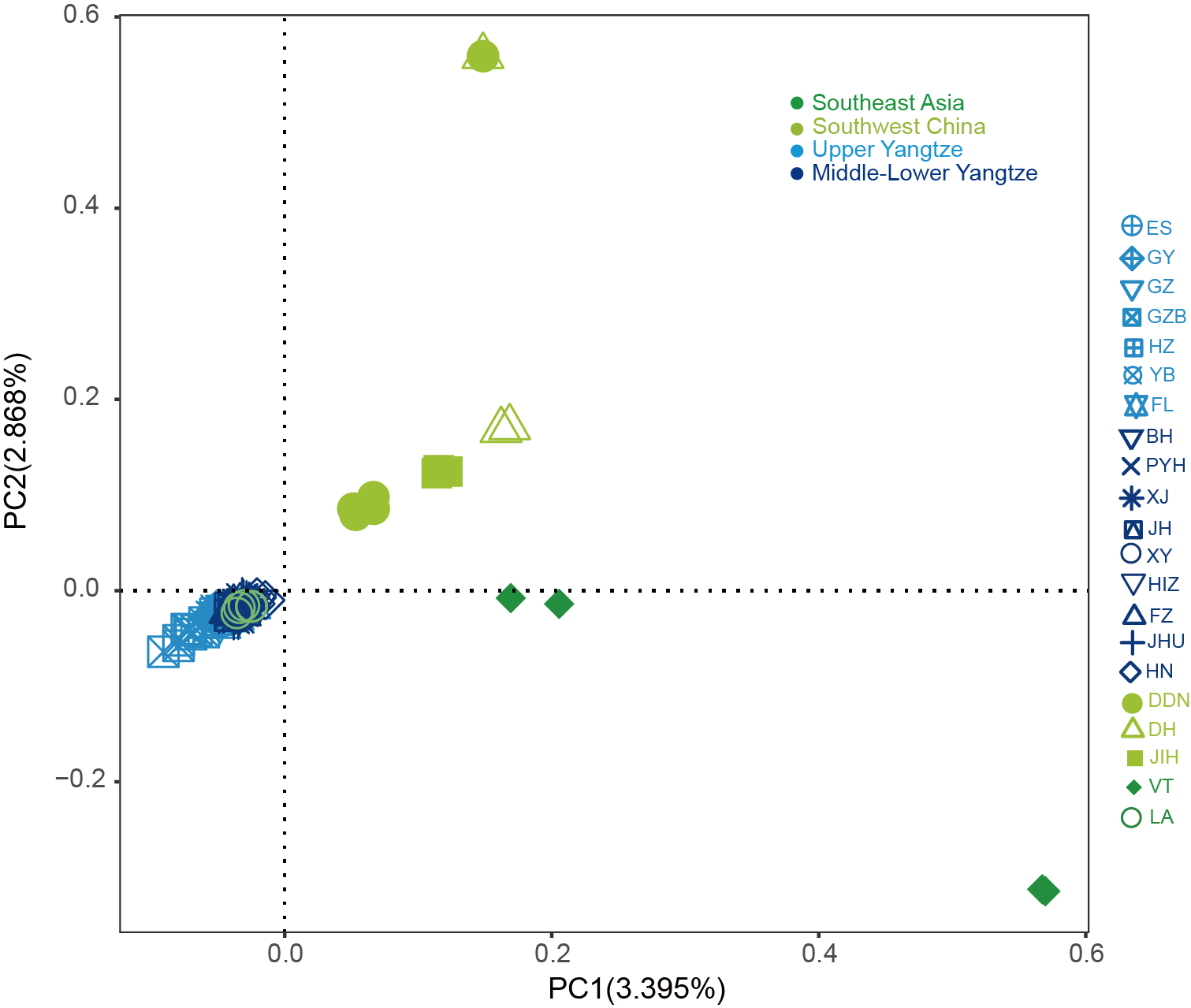


**Supplementary figure 4. PCA of swamp buffaloes with PC1 plotted against PC2.** In total, 89 individuals from 21 swamp buffalo breeds were used for the PCA analysis. The first component separates Southwest Chinese and Vietnam buffaloes from other swamp breeds. Each breed was colored according to their geographic groups.


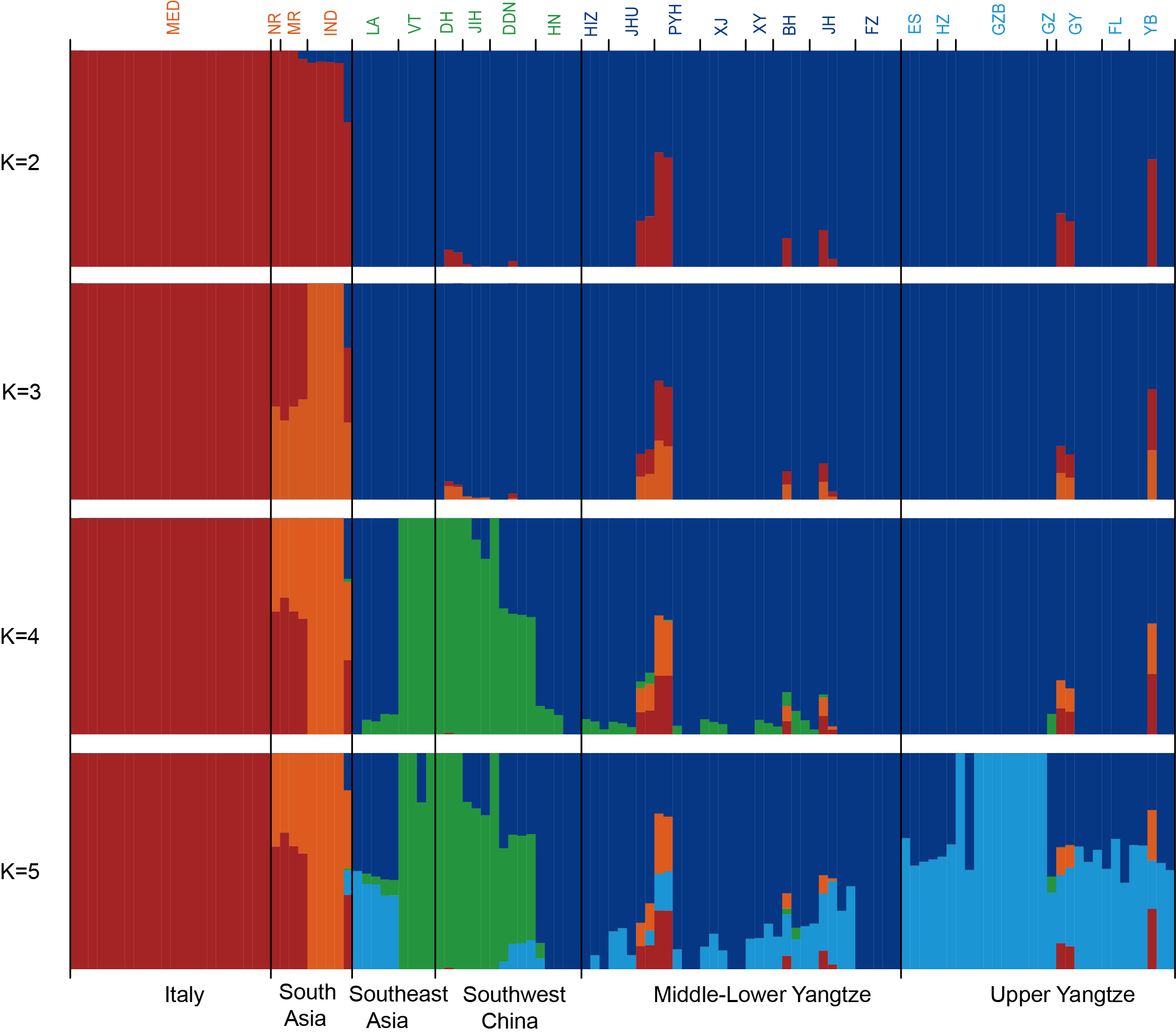


**Supplementary figure 5. Model-based clustering of buffalo using the ADMIXTURE program with *K* from 2 to 5 (plotted with R).** We found strong support for *K* = 2, which is consistent with a deep division of river and swamp buffaloes. When *K* = 3, South Asian buffalo breeds were separated from Italian buffaloes (Mediterranean buffalo). At *K* = 4, all individuals were unambiguously assigned to two ancestries in swamp buffalo, South Chinese (blue) and Southeast Asian (green) components, and other two in river buffalo, South Asian (orange) and Italian (red) components.


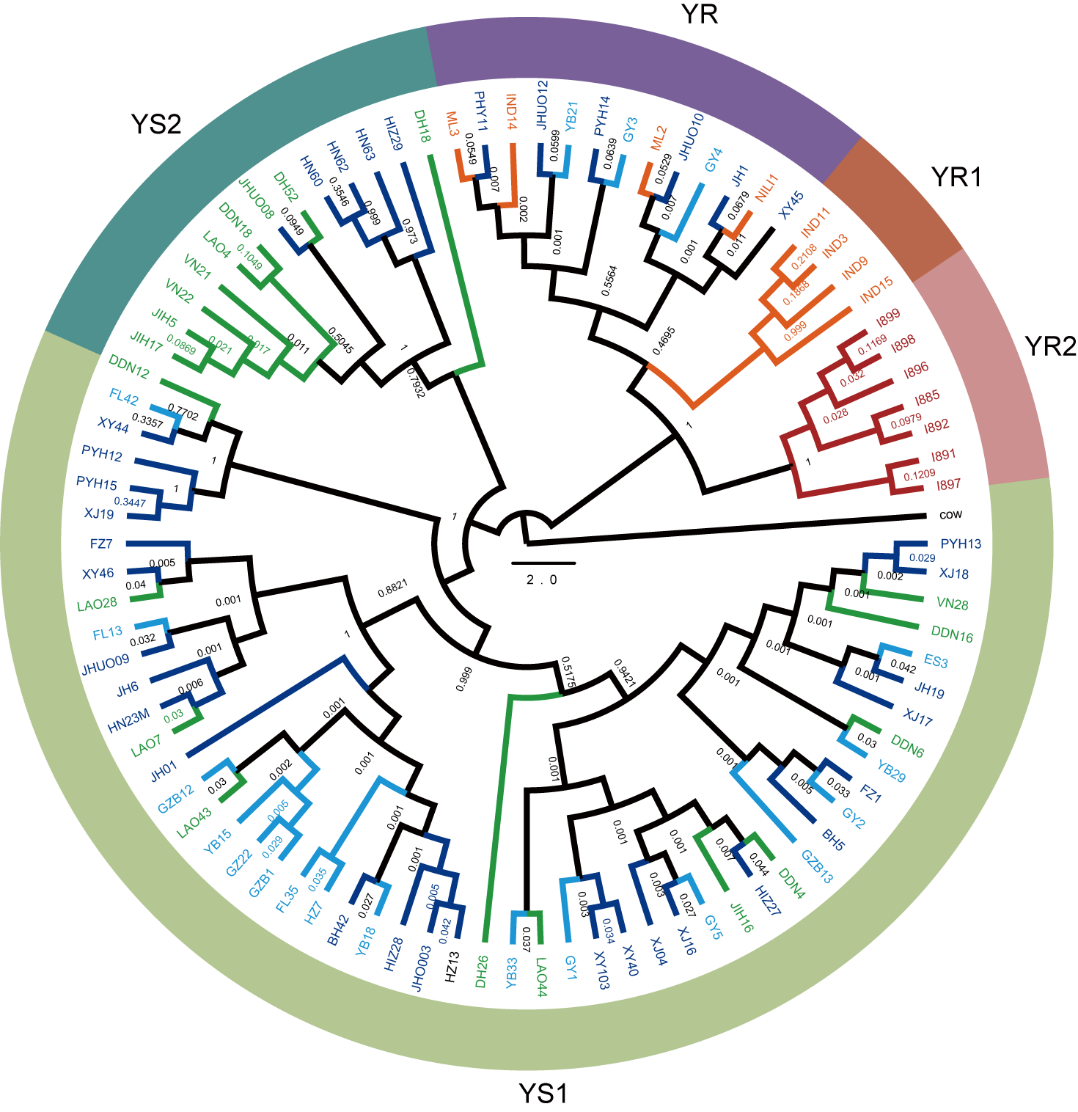


**Supplementary figure 6. ML phylogeny of the Y-chromosome using 520 SNPs for 89 buffaloes.** Labels and nodes are colored according to their geographic groups. Different colors in the external circle represent different haplogroups.


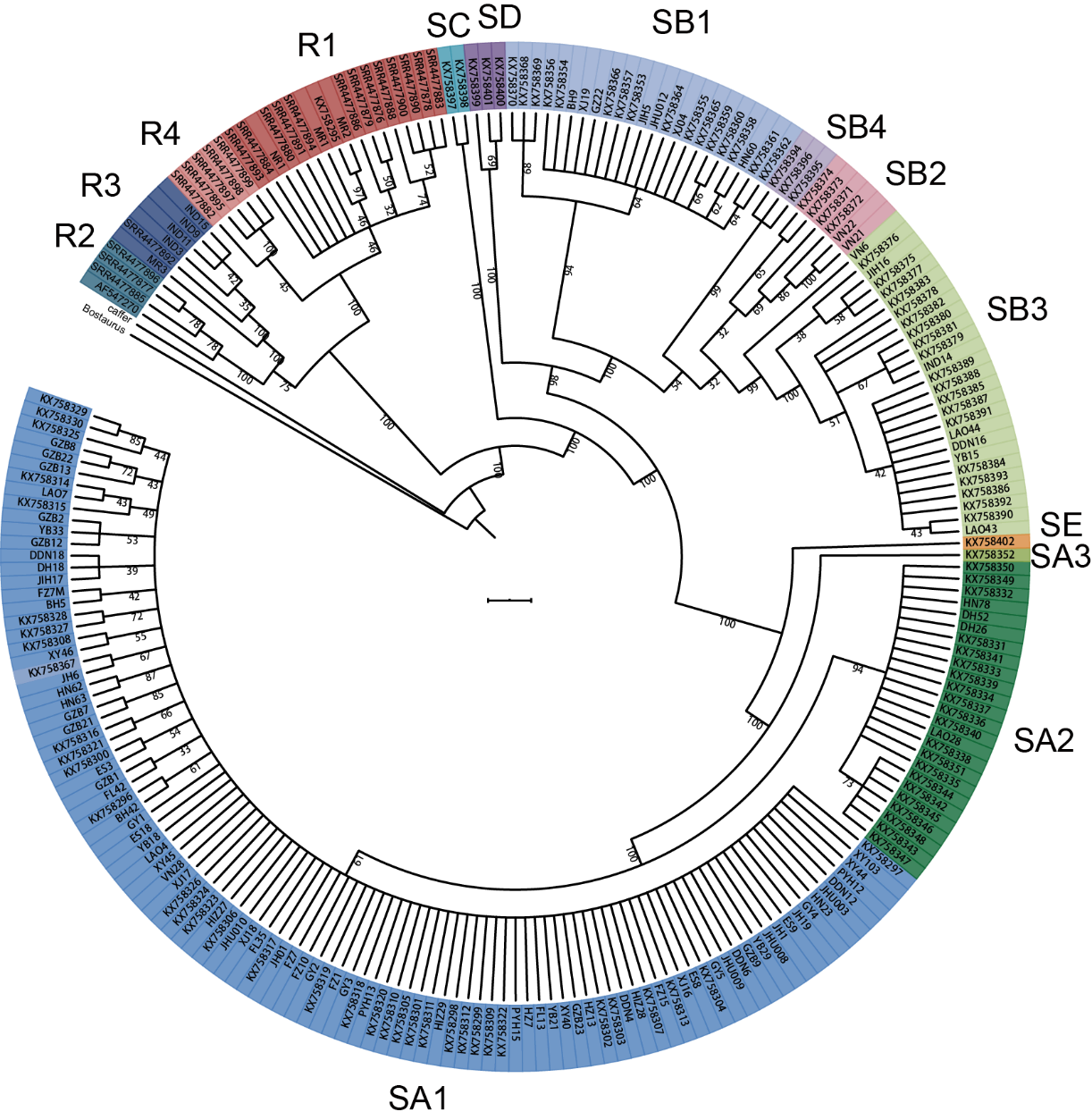


**Supplementary figure 7. ML phylogeny of the mitochondrial genome.** The tree encompassed 225 sequences (including 118 mitogenome sequences from this study and 107 published mtDNA sequences) and was rooted using a published cattle sequence. Node labels are bootstrap values. Different colors in the external circle represent different haplogroups.


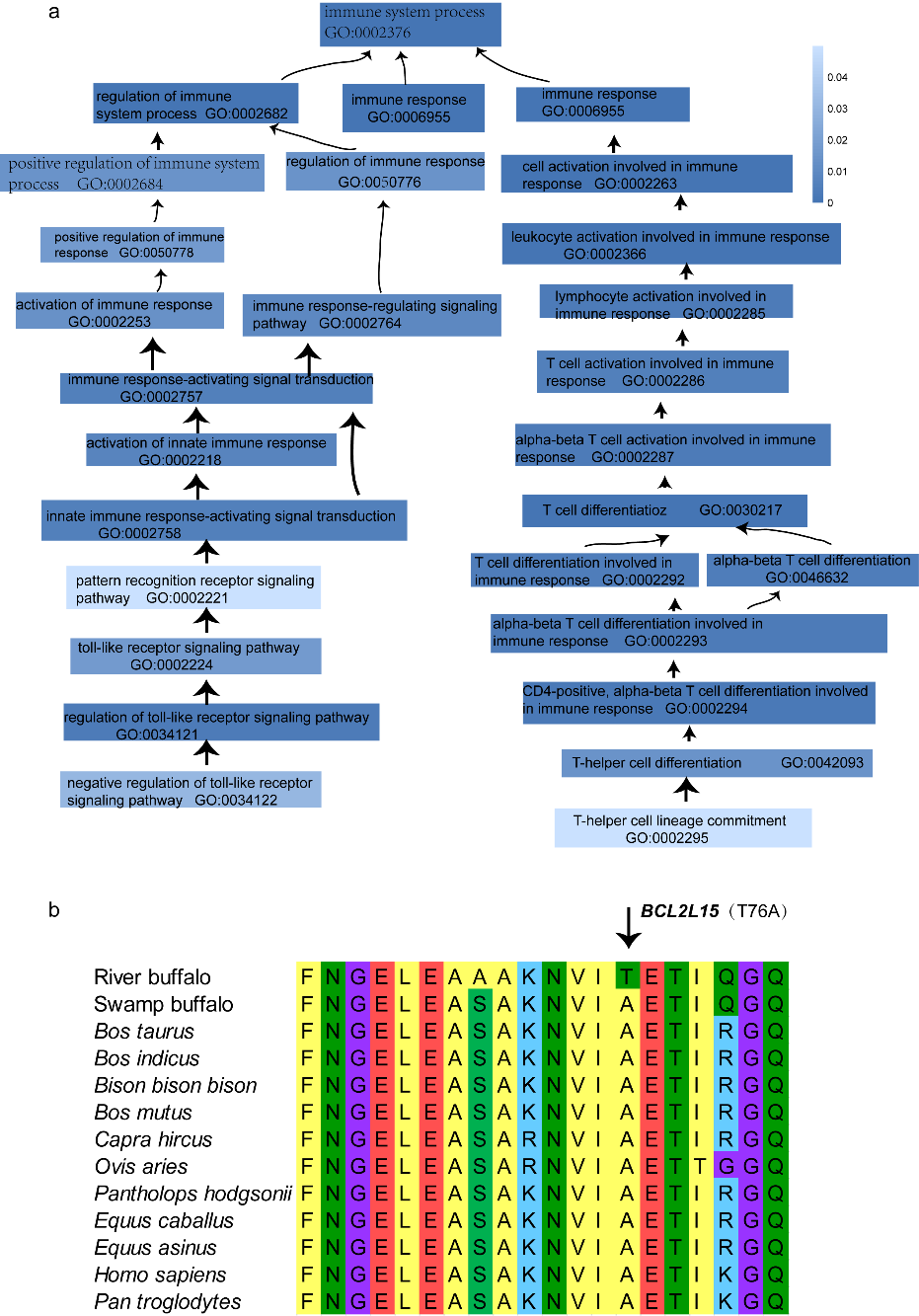


**Supplementary figure 8.** **(a) Hierarchical graph of the over-represented (with significant corrected *p-values* < 0.05) GO terms associated with immunity.** **The color intensity is positively correlated to the corrected *p-value* of the GO term.** **(b) Amino acid conservation of *BCL2L15* (first exon) in mammals. Most amino acids are highly conserved with only few exceptions, including SNP A226G (acid changed: T76A).**


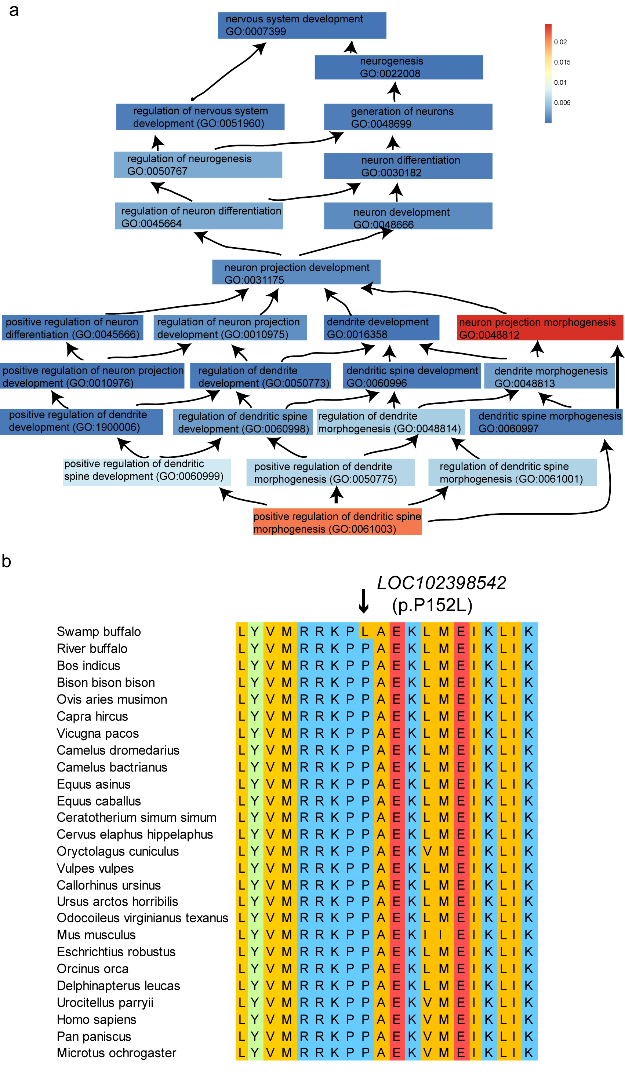


**Supplementary figure 9.** **(a) Hierarchical graph of the over-represented (with significant corrected *p-values* < 0.05) GO terms associated with the nervous system. The color intensity is positively correlated to the corrected *p-value* of the GO term.** **(b) Amino acid conservation of *LOC102398542*. Nonsynonymous** **SNP C455T (acid changed: P152L) located in the first exon Amino acids at this site are highly conserved in other mammals.**

**Supplementary tables**

**Supplementary table 1.** **Overview of sample information and sequencing statistics concerning the 121 buffaloes analyzed in this study.**

| Samples | Breeds | Reference | Abbreviations | Region of origin | Type | Gender | Mapping Rate | Duplication | Insert Size | Mean Depth (X) | 3X | 4X |
| --- | --- | --- | --- | --- | --- | --- | --- | --- | --- | --- | --- | --- |
| ES3 | Enshi | this study | ES | Up Yangtze | Swamp | male | 99.43% | 7.51% | 409 | 10.6976 | 84.71% | 83.11% |
| ES8 | Enshi | this study | ES | Up Yangtze | Swamp | female | 99.44% | 6.97% | 417 | 9.9262 | 99.44% | 83.14% |
| ES9 | Enshi | this study | ES | Up Yangtze | Swamp | female | 98.87% | 6.84% | 407 | 9.1232 | 84.20% | 81.91% |
| ES18 | Enshi | this study | ES | Up Yangtze | Swamp | female | 99.23% | 6.47% | 430 | 8.0702 | 83.30% | 80.08% |
| GY1 | Guangyuan | this study | GY | Up Yangtze | Swamp | male | 99.48% | 8.05% | 403 | 10.1667 | 80.63% | 77.00% |
| GY2 | Guangyuan | this study | GY | Up Yangtze | Swamp | male | 99.32% | 6.73% | 452 | 9.2232 | 83.70% | 81.32% |
| GY3 | Guangyuan | this study | GY | Up Yangtze | Swamp | male | 99.32% | 10.21% | 423 | 10.9135 | 72.41% | 66.76% |
| GY4 | Guangyuan | this study | GY | Up Yangtze | Swamp | male | 99.37% | 11.17% | 422 | 11.9934 | 72.95% | 67.85% |
| GY5 | Guangyuan | this study | GY | Up Yangtze | Swamp | male | 99.18% | 7.54% | 433 | 8.9944 | 78.11% | 73.34% |
| GZB1 | Guizhoubai | this study | GZB | Up Yangtze | Swamp | male | 99.45% | 7.71% | 271 | 22.2774 | 80.90% | 76.75% |
| GZB2 | Guizhoubai | this study | GZB | Up Yangtze | Swamp | female | 99.59% | 9.79% | 423 | 12.1701 | 80.17% | 76.66% |
| GZB7 | Guizhoubai | this study | GZB | Up Yangtze | Swamp | female | 99.34% | 8.78% | 275 | 22.1058 | 77.47% | 71.91% |
| GZB8 | Guizhoubai | this study | GZB | Up Yangtze | Swamp | female | 99.50% | 6.69% | 436 | 9.2987 | 83.85% | 81.33% |
| GZB9 | Guizhoubai | this study | GZB | Up Yangtze | Swamp | male | 99.44% | 7.27% | 275 | 8.595 | 82.56% | 78.85% |
| GZB12 | Guizhoubai | this study | GZB | Up Yangtze | Swamp | male | 99.51% | 7.43% | 434 | 9.6778 | 83.80% | 81.51% |
| GZB13 | Guizhoubai | this study | GZB | Up Yangtze | Swamp | male | 98.73% | 6.82% | 427 | 8.8004 | 82.58% | 79.37% |
| GZB21 | Guizhoubai | this study | GZB | Up Yangtze | Swamp | female | 99.55% | 7.24% | 436 | 9.6406 | 84.57% | 82.90% |
| GZB22 | Guizhoubai | this study | GZB | Up Yangtze | Swamp | female | 99.35% | 5.78% | 442 | 7.089 | 81.25% | 76.23% |
| GZB23 | Guizhoubai | this study | GZB | Up Yangtze | Swamp | female | 99.46% | 7.18% | 441 | 9.0863 | 81.49% | 77.96% |
| GZ22 | Guizhou | this study | GZ | Up Yangtze | Swamp | male | 96.88% | 7.05% | 448 | 8.4639 | 82.13% | 78.01% |
| FL13 | Fuling | this study | FL | Up Yangtze | Swamp | male | 98.46% | 7.74% | 436 | 11.1293 | 84.85% | 83.45% |
| FL35 | Fuling | this study | FL | Up Yangtze | Swamp | male | 99.46% | 7.22% | 398 | 9.9113 | 84.31% | 82.30% |
| FL42 | Fuling | this study | FL | Up Yangtze | Swamp | male | 97.67% | 8.49% | 444 | 11.633 | 84.46% | 82.60% |
| YB15 | Yibin | this study | YB | Up Yangtze | Swamp | male | 99.35% | 7.10% | 433 | 9.1807 | 83.36% | 80.56% |
| YB18 | Yibin | this study | YB | Up Yangtze | Swamp | male | 99.39% | 7.18% | 427 | 8.9831 | 83.23% | 80.25% |
| YB21 | Yibin | this study | YB | Up Yangtze | Swamp | male | 99.31% | 6.87% | 434 | 9.001 | 83.80% | 81.12% |
| YB29 | Yibin | this study | YB | Up Yangtze | Swamp | male | 99.51% | 7.10% | 422 | 8.8706 | 83.51% | 80.79% |
| YB33 | Yibin | this study | YB | Up Yangtze | Swamp | male | 99.49% | 7.26% | 413 | 9.8562 | 84.08% | 81.96% |
| HZ7 | Hanzhong | this study | HZ | Up Yangtze | Swamp | male | 91.36% | 6.98% | 421 | 8.9199 | 83.56% | 80.77% |
| HZ13 | Hanzhong | this study | HZ | Up Yangtze | Swamp | male | 99.29% | 6.35% | 410 | 8.066 | 82.52% | 78.81% |
| BH5 | Binhu | this study | BH | Middle-Lower Yangtze | Swamp | male | 92.95% | 7.45% | 419 | 10.1817 | 84.51% | 82.74% |
| BH9 | Binhu | this study | BH | Middle-Lower Yangtze | Swamp | female | 99.09% | 6.74% | 408 | 9.5314 | 84.59% | 82.85% |
| BH42 | Binhu | this study | BH | Middle-Lower Yangtze | Swamp | male | 98.65% | 9.63% | 410 | 11.9457 | 81.53% | 77.75% |
| HIZ27 | Haizi | this study | HIZ | Middle-Lower Yangtze | Swamp | male | 99.26% | 7.36% | 420 | 10.1021 | 84.27% | 82.33% |
| HIZ28 | Haizi | this study | HIZ | Middle-Lower Yangtze | Swamp | male | 99.14% | 6.47% | 410 | 8.0679 | 82.61% | 78.98% |
| HIZ29 | Haizi | this study | HIZ | Middle-Lower Yangtze | Swamp | male | 98.24% | 6.90% | 424 | 9.5093 | 83.87% | 81.49% |
| HN23M | Hainan | this study | HN | Middle-Lower Yangtze | Swamp | male | 98.86% | 7.19% | 435 | 10.9376 | 84.97% | 83.65% |
| HN60 | Hainan | this study | HN | Middle-Lower Yangtze | Swamp | male | 99.32% | 7.27% | 432 | 9.0885 | 83.39% | 80.61% |
| HN62 | Hainan | this study | HN | Middle-Lower Yangtze | Swamp | male | 99.40% | 7.09% | 438 | 9.4316 | 83.51% | 80.90% |
| HN63 | Hainan | this study | HN | Middle-Lower Yangtze | Swamp | male | 99.40% | 7.14% | 446 | 9.1837 | 83.46% | 80.79% |
| HN78 | Hainan | this study | HN | Middle-Lower Yangtze | Swamp | female | 99.59% | 6.81% | 427 | 8.7879 | 83.71% | 80.97% |
| JH01 | Jianghan | this study | JH | Middle-Lower Yangtze | Swamp | male | 90.46% | 19.55% | 191 | 16.8586 | 83.18% | 79.96% |
| JH1-2 | Jianghan | this study | JH | Middle-Lower Yangtze | Swamp | male | 98.45% | 7.06% | 438 | 9.7231 | 84.30% | 82.19% |
| JH6 | Jianghan | this study | JH | Middle-Lower Yangtze | Swamp | male | 98.47% | 7.23% | 443 | 9.2506 | 83.69% | 81.01% |
| JH19 | Jianghan | this study | JH | Middle-Lower Yangtze | Swamp | male | 99.30% | 8.64% | 439 | 10.0366 | 83.16% | 79.92% |
| JHU003 | Jianghuai | this study | JHU | Middle-Lower Yangtze | Swamp | male | 99.40% | 7.00% | 412 | 9.3141 | 83.92% | 81.69% |
| JHU008 | Jianghuai | this study | JHU | Middle-Lower Yangtze | Swamp | male | 99.40% | 7.37% | 462 | 9.2977 | 83.70% | 81.26% |
| JHU009 | Jianghuai | this study | JHU | Middle-Lower Yangtze | Swamp | male | 99.30% | 7.06% | 445 | 8.6669 | 83.05% | 79.95% |
| JHUO10 | Jianghuai | this study | JHU | Middle-Lower Yangtze | Swamp | male | 99.30% | 7.10% | 460 | 9.2776 | 83.58% | 80.94% |
| JHU012 | Jianghuai | this study | JHU | Middle-Lower Yangtze | Swamp | male | 99.40% | 7.37% | 418 | 8.7486 | 82.85% | 79.39% |
| PYH11 | Poyanghu | this study | PYH | Middle-Lower Yangtze | Swamp | male | 98.91% | 12.96% | 465 | 8.0149 | 79.79% | 73.69% |
| PYH12 | Poyanghu | this study | PYH | Middle-Lower Yangtze | Swamp | male | 99.52% | 7.05% | 427 | 8.6682 | 83.25% | 80.30% |
| PYH13 | Poyanghu | this study | PYH | Middle-Lower Yangtze | Swamp | male | 99.39% | 7.12% | 457 | 9.5857 | 83.83% | 81.56% |
| PYH14 | Poyanghu | this study | PYH | Middle-Lower Yangtze | Swamp | male | 99.42% | 6.86% | 421 | 9.2404 | 84.09% | 81.68% |
| PYH15 | Poyanghu | this study | PYH | Middle-Lower Yangtze | Swamp | male | 99.40% | 7.37% | 464 | 9.164 | 83.50% | 80.82% |
| XJ04 | Xiajiang | this study | XJ | Middle-Lower Yangtze | Swamp | male | 99.51% | 7.80% | 418 | 9.7263 | 83.94% | 81.57% |
| XJ16 | Xiajiang | this study | XJ | Middle-Lower Yangtze | Swamp | male | 99.53% | 7.53% | 434 | 19.0711 | 84.15% | 82.07% |
| XJ17 | Xiajiang | this study | XJ | Middle-Lower Yangtze | Swamp | male | 99.46% | 7.49% | 422 | 17.9957 | 83.69% | 81.13% |
| XJ18 | Xiajiang | this study | XJ | Middle-Lower Yangtze | Swamp | male | 99.46% | 7.21% | 430 | 9.8521 | 84.27% | 82.30% |
| XJ19 | Xiajiang | this study | XJ | Middle-Lower Yangtze | Swamp | male | 99.38% | 6.95% | 415 | 9.3327 | 83.89% | 81.52% |
| XY40 | Xingyang | this study | XY | Middle-Lower Yangtze | Swamp | male | 99.55% | 7.66% | 424 | 9.8895 | 84.35% | 82.44% |
| XY44 | Xingyang | this study | XY | Middle-Lower Yangtze | Swamp | male | 99.40% | 6.87% | 428 | 9.1571 | 83.72% | 81.24% |
| XY45 | Xingyang | this study | XY | Middle-Lower Yangtze | Swamp | male | 99.48% | 7.65% | 429 | 10.3049 | 84.45% | 82.61% |
| XY46 | Xingyang | this study | XY | Middle-Lower Yangtze | Swamp | male | 99.10% | 7.17% | 426 | 9.4266 | 82.41% | 79.38% |
| XY103 | Xingyang | this study | XY | Middle-Lower Yangtze | Swamp | male | 96.96% | 7.29% | 428 | 9.9194 | 84.07% | 81.91% |
| FZ1 | Fuzhou | this study | FZ | Middle-Lower Yangtze | Swamp | male | 99.04% | 6.82% | 405 | 9.5409 | 84.65% | 82.93% |
| FZ7 | Fuzhou | this study | FZ | Middle-Lower Yangtze | Swamp | male | 99.08% | 6.14% | 417 | 7.7134 | 82.81% | 78.96% |
| FZ7M | Fuzhou | this study | FZ | Middle-Lower Yangtze | Swamp | female | 99.02% | 6.62% | 407 | 8.3402 | 82.96% | 79.61% |
| FZ10 | Fuzhou | this study | FZ | Middle-Lower Yangtze | Swamp | female | 99.24% | 6.60% | 404 | 8.8441 | 84.20% | 81.90% |
| FZ15 | Fuzhou | this study | FZ | Middle-Lower Yangtze | Swamp | female | 99.26% | 6.70% | 406 | 8.8073 | 83.53% | 80.76% |
| JIH5 | Jinghong | this study | JIH | Southwest China | Swamp | male | 97.17% | 7.09% | 413 | 10.18 | 84.60% | 82.93% |
| JIH16 | Jinghong | this study | JIH | Southwest China | Swamp | male | 99.16% | 6.07% | 443 | 8.4216 | 83.42% | 80.70% |
| JIH17 | Jinghong | this study | JIH | Southwest China | Swamp | male | 98.13% | 7.27% | 403 | 10.8411 | 84.90% | 83.49% |
| DDN4 | Diandongnan | this study | DDN | Southwest China | Swamp | male | 98.76% | 7.11% | 428 | 10.4433 | 84.71% | 83.18% |
| DDN6 | Diandongnan | this study | DDN | Southwest China | Swamp | male | 99.33% | 6.73% | 415 | 9.2667 | 84.07% | 81.89% |
| DDN12 | Diandongnan | this study | DDN | Southwest China | Swamp | male | 99.15% | 6.59% | 458 | 9.0473 | 83.75% | 81.39% |
| DDN16 | Diandongnan | this study | DDN | Southwest China | Swamp | male | 99.52% | 7.02% | 414 | 10.2357 | 84.59% | 82.98% |
| DDN18 | Diandongnan | this study | DDN | Southwest China | Swamp | male | 99.46% | 6.98% | 434 | 10.166 | 84.52% | 82.87% |
| DH18 | Dehong | this study | DH | Southwest China | Swamp | male | 99.46% | 6.98% | 434 | 10.166 | 84.52% | 82.87% |
| DH26 | Dehong | this study | DH | Southwest China | Swamp | male | 99.05% | 7.10% | 428 | 8.7045 | 82.53% | 78.78% |
| DH52 | Dehong | this study | DH | Southwest China | Swamp | male | 99.33% | 7.28% | 426 | 10.4784 | 84.64% | 82.96% |
| VN6 | Vietnam | this study | VN | Southeast Asia | Swamp | female | 98.42% | 7.51% | 451 | 9.1137 | 83.60% | 80.79% |
| VN21 | Vietnam | this study | VN | Southeast Asia | Swamp | male | 98.42% | 6.92% | 464 | 17.2677 | 83.08% | 80.09% |
| VN22 | Vietnam | this study | VN | Southeast Asia | Swamp | male | 99.16% | 7.03% | 449 | 18.613 | 83.58% | 81.05% |
| VN28 | Vietnam | this study | VN | Southeast Asia | Swamp | male | 98.67% | 7.97% | 448 | 10.4595 | 84.08% | 81.97% |
| LAO4 | Laos | this study | LA | Southeast Asia | Swamp | male | 99.37% | 7.93% | 427 | 10.8236 | 84.75% | 83.22% |
| LAO7 | Laos | this study | LA | Southeast Asia | Swamp | male | 98.17% | 7.40% | 443 | 7.7952 | 81.07% | 76.06% |
| LAO28 | Laos | this study | LA | Southeast Asia | Swamp | male | 98.70% | 7.28% | 442 | 8.7079 | 82.69% | 79.22% |
| LAO43 | Laos | this study | LA | Southeast Asia | Swamp | male | 97.85% | 6.61% | 471 | 7.0965 | 78.93% | 72.37% |
| LAO44 | Laos | this study | LA | Southeast Asia | Swamp | male | 98.93% | 7.17% | 448 | 8.3539 | 82.17% | 78.26% |
| NILI1 | Nili-Ravi | this study | NR | South Asia | River | male | 99.42% | 6.78% | 438 | 9.6986 | 84.64% | 82.75% |
| Murrah01 | Murrah | SRR032564,SRR034148 | MR | South Asia | River | female | 90.45% | 18.59% | 175 | 14.2386 | 84.94% | 83.01% |
|  |  | SRR034232(NCBI) |  |  |  |  |  |  |  |  |  |  |
| MR2 | Murrah | this study | MR | South Asia | River | male | 99.46% | 6.60% | 442 | 9.5247 | 84.52% | 82.53% |
| MR3 | Murrah | this study | MR | South Asia | River | male | 99.49% | 9.70% | 437 | 18.1128 | 86.44% | 85.95% |
| IND3 | India | this study | IND | South Asia | River | male | 99.39% | 7.16% | 436 | 18.6814 | 83.00% | 80.14% |
| IND9 | India | this study | IND | South Asia | River | male | 99.50% | 7.45% | 434 | 19.2273 | 85.01% | 83.47% |
| IND11 | India | this study | IND | South Asia | River | male | 99.51% | 7.02% | 418 | 9.9089 | 84.37% | 82.34% |
| IND14 | India | this study | IND | South Asia | hybrid | male | 99.39% | 6.84% | 453 | 9.2374 | 84.07% | 81.71% |
| IND15 | India | this study | IND | South Asia | River | male | 99.42% | 6.65% | 459 | 8.9954 | 83.73% | 81.12% |
| SRR4477876 | Mediterranean | [[1]](#RANGE!_ENREF_1) | MD | Italy | River | female | 99.76% | 6.43% | 254 | 6.7125 | 77.78% | 71.77% |
| SRR4477877 | Mediterranean | [[1]](#RANGE!_ENREF_1) | MD | Italy | River | female | 99.74% | 7.36% | 247 | 7.9414 | 78.79% | 74.31% |
| SRR4477878 | Mediterranean | [[1]](#RANGE!_ENREF_1) | MD | Italy | River | female | 99.77% | 6.94% | 228 | 7.4651 | 79.61% | 74.93% |
| SRR4477879 | Mediterranean | [[1]](#RANGE!_ENREF_1) | MD | Italy | River | female | 99.77% | 7.48% | 222 | 8.3378 | 81.44% | 77.76% |
| SRR4477880 | Mediterranean | [[1]](#RANGE!_ENREF_1) | MD | Italy | River | female | 99.77% | 6.20% | 211 | 6.5879 | 78.69% | 72.71% |
| SRR4477882 | Mediterranean | [[1]](#RANGE!_ENREF_1) | MD | Italy | River | female | 99.77% | 7.53% | 218 | 8.2581 | 81.18% | 77.28% |
| SRR4477883 | Mediterranean | [[1]](#RANGE!_ENREF_1) | MD | Italy | River | female | 99.79% | 6.85% | 206 | 7.2583 | 81.07% | 76.29% |
| SRR4477884 | Mediterranean | [[1]](#RANGE!_ENREF_1) | MD | Italy | River | female | 99.70% | 6.56% | 212 | 5.7224 | 74.06% | 66.44% |
| SRR4477885 | Mediterranean | [[1]](#RANGE!_ENREF_1) | MD | Italy | River | male | 99.44% | 6.59% | 213 | 5.5289 | 71.97% | 63.74% |
| SRR4477886 | Mediterranean | [[1]](#RANGE!_ENREF_1) | MD | Italy | River | female | 99.68% | 6.58% | 241 | 5.6185 | 72.57% | 64.74% |
| SRR4477888 | Mediterranean | [[1]](#RANGE!_ENREF_1) | MD | Italy | River | female | 99.73% | 7.37% | 213 | 6.6526 | 77.60% | 71.62% |
| SRR4477890 | Mediterranean | [[1]](#RANGE!_ENREF_1) | MD | Italy | River | female | 99.08% | 8.13% | 413 | 9.568 | 85.40% | 83.91% |
| SRR4477891 | Mediterranean | [[1]](#RANGE!_ENREF_1) | MD | Italy | River | male | 99.09% | 8.48% | 415 | 10.6283 | 85.36% | 83.90% |
| SRR4477892 | Mediterranean | [[1]](#RANGE!_ENREF_1) | MD | Italy | River | male | 98.84% | 8.58% | 388 | 10.1737 | 85.44% | 83.89% |
| SRR4477893 | Mediterranean | [[1]](#RANGE!_ENREF_1) | MD | Italy | River | female | 99.15% | 8.52% | 391 | 9.9779 | 85.65% | 84.33% |
| SRR4477894 | Mediterranean | [[1]](#RANGE!_ENREF_1) | MD | Italy | River | female | 99.53% | 11.53% | 364 | 14.3563 | 86.52% | 85.87% |
| SRR4477895 | Mediterranean | [[1]](#RANGE!_ENREF_1) | MD | Italy | River | female | 99.07% | 7.24% | 373 | 7.7817 | 84.22% | 81.26% |
| SRR4477896 | Mediterranean | [[1]](#RANGE!_ENREF_1) | MD | Italy | River | male | 99.33% | 10.06% | 424 | 12.1531 | 85.93% | 84.96% |
| SRR4477897 | Mediterranean | [[1]](#RANGE!_ENREF_1) | MD | Italy | River | male | 98.85% | 7.88% | 402 | 8.5269 | 84.37% | 81.83% |
| SRR4477898 | Mediterranean | [[1]](#RANGE!_ENREF_1) | MD | Italy | River | male | 99.11% | 8.28% | 445 | 9.0016 | 84.56% | 82.36% |
| SRR4477899 | Mediterranean | [[1]](#RANGE!_ENREF_1) | MD | Italy | River | male | 99.32% | 11.04% | 346 | 11.5496 | 86.03% | 84.92% |
| SRR4477900 | Mediterranean | [[1]](#RANGE!_ENREF_1) | MD | Italy | River | female | 99.04% | 6.46% | 356 | 5.9717 | 80.60% | 73.74% |

**Note:** NILI1, MR2, and MR2 were sampled from China, and were imported from Pakistan, and India, respectively.

**Supplementary table 2. Summary information on the 25 buffalo breeds.**

| Breeds | Abbreviations | Animal count | Type | Study | Longitude | | Latitude | Geographic distribution |
| --- | --- | --- | --- | --- | --- | --- | --- | --- |
| Guangyuan | GY | 5 | Swamp | this study | | 105.739285 | 32.434557 | Sichuan province |
| Guizhoubai | GZB | 10 | Swamp | this study | | 107.714689 | 27.957737 | Fenggang,Zunyi city,guizhou province |
| Guizhou | GZ | 1 | Swamp | this study | | 107.178268 | 28.545459 | North of Guizhou province |
| Fuling | FL | 3 | Swamp | this study | | 107.270953 | 29.674330 | Fuling, Chongqing City |
| Yibin | YB | 5 | Swamp | this study | | 104.642735 | 28.752063 | Yibin,Sichuan province |
| Hanzhong | HZ | 2 | Swamp | this study | | 107.715420 | 32.913346 | Hanzhong, Shaanxi province |
| Fuzhou | FZ | 5 | Swamp | this study | | 119.673220 | 27.117908 | Fu'an, Fujian province |
| Haizi | HIZ | 3 | Swamp | this study | | 120.499934 | 32.732401 | Jiangsu |
| Poyanghu | PYH | 5 | Swamp | this study | | 116.861640 | 29.298113 | Poyang, Jiangxi province |
| Binhu | BH | 3 | Swamp | this study | | 113.133035 | 29.354326 | Yueyang, Hunan province |
| Xiajiang | XJ | 5 | Swamp | this study | | 115.247891 | 27.614814 | Xiajiang, Ji'an,Jiangxi province |
| Xinyang | XY | 5 | Swamp | this study | | 114.127219 | 32.101693 | Xinyang,Henan province |
| Jianghan | JH | 4 | Swamp | this study | | 112.264773 | 30.329550 | Jingzhou, Hubei province |
| Jianghuai | JHU | 5 | Swamp | this study | | 116.480439 | 31.737813 | Liu'an, Anhui province |
| Hainan | HN | 5 | Swamp | this study | | 110.202068 | 18.756250 | Xinglong, Wanning, Hainan province |
| Enshi | ES | 4 | Swamp | this study | | 109.512550 | 30.408398 | Enshi, Hubei province |
| Jinghong | JIH | 3 | Swamp | this study | | 100.947482 | 22.217163 | Jinghong, Xishuangbanna, Yunnan province |
| Diandongnan | DDN | 5 | Swamp | this study | | 102.917610 | 23.801018 | Honghe, Yunnan province |
| Dehong | DH | 3 | Swamp | this study | | 97.879080 | 24.247805 | Dehong, Yunnan province |
| Laos | LA | 5 | Swamp | this study | | 102.187332 | 21.653561 | China-Laos border |
| Vietnam | VN | 4 | Swamp | this study | | 105.015774 | 22.762678 | China-Vietnam border |
| India | IND | 5 | River | this study | | 81.997303 | 27.221269 | North India |
| Murrah | MR | 3 | River | two from this study; and one NCBI | | 108.557395 | 22.951182 | India |
| Nili-Ravi | NR | 1 | River | this study | | 108.557395 | 22.951182 | Pakistan |
| Mediterranean | MD | 22 | River | [[4](#_ENREF_4)] | |  |  |  |

**Supplementary table 3. Distribution of SNPs within various genomic regions.**

| Variant type | SNP Count |
| --- | --- |
| Total number | 34,352,893 |
| Intergenic | 20,867,720 |
| Intronic | 11427068 |
| Downstream | 282,420 |
| Upstream | 269964 |
| Exonic | 458119 |
| UTR | 285,954 |
| Synonymous | 153,317 |
| Nonsynonymous | 69,396 |
| Splicing | 5,401 |
| Stop gain | 831 |
| Stop loss | 85 |
| Others | 990,737 |
| Ratio of nonsynonymous/synonymous | 0.452630824 |

**Supplementary table 4. Summary information on the linked pseudo-chromosomes.**

| Pseudo-chromosome | Number of scaffolds | Total Size | Average size of scaffold | N50 of scaffold |
| --- | --- | --- | --- | --- |
| Chr1 | 16386 | 233,117,896 | 13,227 | 1,445,645 |
| Chr2 | 32028 | 244,125,176 | 6,622 | 1,492,582 |
| Chr3 | 12552 | 206,763,389 | 15,473 | 1,910,177 |
| Chr4 | 21999 | 192,336,607 | 7,743 | 1,175,522 |
| Chr5 | 13071 | 153,052,043 | 10,709 | 1,378,483 |
| Chr6 | 11721 | 130,030,428 | 10,094 | 1,371,224 |
| Chr7 | 23247 | 155,389,619 | 5,684 | 1,241,020 |
| Chr8 | 8631 | 135,979,981 | 14,755 | 1,460,441 |
| Chr9 | 8066 | 126,563,782 | 14,691 | 1,268,121 |
| Chr10 | 10289 | 121,683,129 | 10,827 | 1,528,830 |
| Chr11 | 7029 | 107,235,950 | 14,256 | 1,574,705 |
| Chr12 | 7001 | 119,871,756 | 16,122 | 1,660,966 |
| Chr13 | 5669 | 111,957,700 | 18,749 | 1,727,873 |
| Chr14 | 6009 | 92,181,728 | 14,341 | 1,550,336 |
| Chr15 | 9388 | 95,382,903 | 9,160 | 1,350,111 |
| Chr16 | 24474 | 122,970,810 | 4,025 | 1,097,950 |
| Chr17 | 4362 | 79,684,951 | 17,268 | 1,488,780 |
| Chr18 | 4737 | 69,030,298 | 13,573 | 1,322,868 |
| Chr19 | 4142 | 70,946,101 | 16,129 | 1,215,911 |
| Chr20 | 6679 | 78,437,469 | 10,744 | 1,068,218 |
| Chr21 | 4463 | 74,390,089 | 15,668 | 2,201,260 |
| Chr22 | 4208 | 73,599,655 | 16,491 | 1,526,019 |
| Chr23 | 3512 | 54,224,151 | 14,440 | 2,133,153 |
| Chr24 | 3315 | 70,496,478 | 20,266 | 2,115,015 |
| ChrX | 12434 | 146,132,119 | 10,753 | 811,811 |
| unplace | 3821 | 6,877,613 | 800 | 824 |
| MIT | 1 | 16,359 | 16,359 | 16,359 |

**Supplementary table 5. The θ_π_ value for the buffalo population groups.**

| Region | Breeds | Type | Sample size | *θ*_π_ (×10^-3^) |
| --- | --- | --- | --- | --- |
| Up Yangtze | Enshi | Swamp | 4 | 1.68934 |
|  | Fuling | Swamp | 3 | 1.76313 |
|  | Yibin | Swamp | 4 | 1.66563 |
|  | Guizhou | Swamp | 1 | - |
|  | Guizhoubai | Swamp | 10 | 1.59776 |
|  | Guangyuan | Swamp | 3 | 1.62372 |
|  | Hanzhong | Swamp | 2 | - |
|  | Total |  | 27 | 1.6055 |
| Middle-Lower Yangtze | Binhu | Swamp | 3 | 1.73136 |
|  | Fuzhou | Swamp | 5 | 1.64184 |
|  | Haizi | Swamp | 3 | 1.74687 |
|  | Jianghan | Swamp | 3 | 1.77969 |
|  | Jianghuai | Swamp | 3 | 1.71286 |
|  | Poyanghu | Swamp | 3 | 1.65943 |
|  | Xiajiang | Swamp | 5 | 1.6869 |
|  | Xinyang | Swamp | 4 | 1.71684 |
|  | Total |  | 29 | 1.64649 |
| Southwest China | Diandongnan | Swamp | 5 | 1.82252 |
|  | Ddehong | Swamp | 3 | 1.93344 |
|  | Jinghong | Swamp | 3 | 1.87901 |
|  | Hainan | Swamp | 5 | 1.61664 |
|  | Total |  | 16 | 1.73552 |
| Southeast Asia | Vietnam | Swamp | 4 | 1.62368 |
|  | Laos | Swamp | 5 | 1.64618 |
|  | Total |  | 9 | 1.65817 |
| South Asia | Indian | River | 4 | 1.55116 |
|  | Murrah | River | 3 | 2.25486 |
|  | Nili-Ravi | River | 1 | - |
|  | Total |  | 8 | 2.21255 |
| Italy | Mediterranean | River | 22 | 1.59321 |

Note: cross-breed samples were excluded.

**Supplementary table 6. Pairwise *F*_ST_ values. On the continental scale, *F*_ST_ values were correlated with geographical distances between populations.**

| Population | Middle-Lower Yangtze | Southeast Asia | Up Yangtze | Southwest China | South Asia |
| --- | --- | --- | --- | --- | --- |
| Southeast Asia | 0.017 |  |  |  |  |
| Up Yangtze | 0.012 | 0.022 |  |  |  |
| Southwest China | 0.036 | 0.046 | 0.046 |  |  |
| South Asia | 0.278 | 0.257 | 0.284 | 0.304 |  |
| Italy | 0.323 | 0.301 | 0.330 | 0.351 | 0.143 |

**Supplementary table 7. Tracy-Widom (TW) statistics and *p-value*s for the ten first eigenvalues in the PCA of buffaloes.**

| Number | eigenvalue | TW | *p-value* |  |
| --- | --- | --- | --- | --- |
| 1 | 14.07822 | 56.492 | 5.98E-125 |  |
| 2 | 4.097363 | 59.671 | 1.77E-135 |  |
| 3 | 2.59635 | 34.671 | 4.55E-61 |  |
| 4 | 2.392697 | 35.009 | 6.16E-62 |  |
| 5 | 2.171265 | 32.943 | 1.06E-56 |  |
| 6 | 1.724569 | 15.096 | 7.46E-19 |  |
| 7 | 1.660906 | 14.31 | 1.54E-17 |  |
| 8 | 1.534869 | 9.143 | 8.04E-10 |  |
| 9 | 1.382001 | 1.056 | 0.0449206 |  |

**Supplementary table 8. Cross-Validation (CV) error for different ADMIXTURE models (K from 2 to 5).**

| *K* value | CV error |
| --- | --- |
| *K* = 2 | 0.34528 |
| *K* = 3 | 0.35289 |
| *K* = 4 | 0.34788 |
| *K* = 5 | 0.35639 |

**Supplementary References**

1. Amaral MEJ, Grant JR, Riggs PK, Stafuzza NB, Filho EAR, Goldammer T, et al. A first generation whole genome RH map of the river buffalo with comparison to domestic cattle. BMC Genomics. 2008;9 1:631. doi:10.1186/1471-2164-9-631.

2. Li H and Durbin R. Fast and accurate short read alignment with Burrows–Wheeler transform. Bioinformatics. 2009;25 14:1754-60. doi:10.1093/bioinformatics/btp324.

3. Nekrutenko A and Taylor J. Next-generation sequencing data interpretation: enhancing reproducibility and accessibility. Nature Reviews Genetics. 2012;13 9:667-72. doi:10.1038/nrg3305.

4. Whitacre LK, Hoff JL, Schnabel RD, Albarella S, Ciotola F, Peretti V, et al. Elucidating the genetic basis of an oligogenic birth defect using whole genome sequence data in a non-model organism, Bubalus bubalis. Scientific Reports. 2017;7:39719. doi:10.1038/srep39719.

5. Shaun P, Benjamin N, Kathe TB, Lori T, Ferreira MAR, David B, et al. PLINK: a tool set for whole-genome association and population-based linkage analyses. American Journal of Human Genetics. 2007; doi:10.1086/519795.

6. Patterson N, Price AL and Reich D. Population Structure and Eigenanalysis. PLOS Genetics. 2006;2 12:e190. doi:10.1371/journal.pgen.0020190.

7. Colli L, Milanesi M, Vajana E, Iamartino D, Bomba L, Puglisi F, et al. New Insights on Water Buffalo Genomic Diversity and Post-Domestication Migration Routes From Medium Density SNP Chip Data. Frontiers in Genetics. 2018;9 53 doi:10.3389/fgene.2018.00053.

8. Alexander DH, Novembre J and Lange K. Fast model-based estimation of ancestry in unrelated individuals. Genome Research. 2009;19 9:1655-64. doi:10.1101/gr.094052.109.

9. Chen L, Qiu Q, Jiang Y, Wang K, Lin Z, Li Z, et al. Large-scale ruminant genome sequencing provides insights into their evolution and distinct traits. Science. 2019;364 6446:eaav6202. doi:10.1126/science.aav6202.

10. Pickrell JK and Pritchard JK. Inference of Population Splits and Mixtures from Genome-Wide Allele Frequency Data. PLOS Genetics. 2012;8 11:e1002967. doi:10.1371/journal.pgen.1002967.

11. Browning SR and Browning BL. Rapid and accurate haplotype phasing and missing-data inference for whole-genome association studies by use of localized haplotype clustering. American journal of human genetics. 2007;81 5:1084-97. doi:10.1086/521987.

12. Paradis E. pegas: an R package for population genetics with an integrated–modular approach. Bioinformatics. 2010;26 3:419-20. doi:10.1093/bioinformatics/btp696.

13. Bouckaert R, Heled J, Kühnert D, Vaughan T, Wu C-H, Xie D, et al. BEAST 2: A Software Platform for Bayesian Evolutionary Analysis. PLOS Computational Biology. 2014;10 4:e1003537. doi:10.1371/journal.pcbi.1003537.

14. Briggs AW, Good JM, Green RE, Krause J, Maricic T, Stenzel U, et al. Targeted Retrieval and Analysis of Five Neandertal mtDNA Genomes. Science. 2009;325 5938:318. doi:10.1126/science.1174462.
