# Supplementary material for: Genomic analyses reveal distinct genetic architectures and selective pressures in buffaloes
Source: Gigascience. 2020 Feb 21;9(2):giz166. doi: 10.1093/gigascience/giz166 (PMC7033652; doi:10.1093/gigascience/giz166)
Supplement: giz166_Response_to_Reviewer_Comments_Original_Submission [file giz166_response_to_reviewer_comments_original_submission.pdf]

Author's Response To Reviewer Comments

Close

Response to Reviewers' comments

Genomic Analyses Reveal Distinct Genetic Architectures and Selective Pressures in Buffaloes
GigaScience
Dear Zhou,
Thank you very much for handling our manuscript Manuscript ID entitled " Genomic Analyses Reveal Distinct Genetic Architectures and Selective Pressures in Buffaloes" (GIGA-D-19-00183). We appreciate all the comments from the reviewers, which helped us to improve our manuscript. We have now revised the manuscript according to the reviewers’ comments and your instructions.
We addressed the comments and questions of the reviewers as explained below; we have modified the manuscript according to the suggestions of the reviewers. Revised sentences are marked in red in the paper.

Reviewer reports:
[Reviewer #1:]
Karyotyping for confirmation of riverine, swamp or hybrid buffaloes has not been done. Then, how, the classification of the groups has been done?
Response: Riverine buffaloes and swamp buffaloes belong to two types of buffalo, with divergent genomes and karyotypes. According to the results of NJ tree, ML tree, ADMIXTURE, and PCA using the whole-genome SNP information, the buffaloes can be clearly divided into river buffaloes, swamp buffaloes, and hybrid buffaloes. Furthermore, the uniparental markers (Y chromosomal and mitochondrial DNA) have been also used as a further confirmation.

No out group has been taken in the phylogenetic analysis.
Response: Actually, we used the Syncerus caffer as outgroup to perform the phylogenetic analysis as shown in the Figure 1b and Supplementary figure 2. This information has been added in the revised text (Line 107 - Line 108).

There is no mention about the parameters used in the various tools in the study.
Response: The necessary parameters are provided in the methods and the supplementary notes, please check.

Many of the notations used in the supplementary need to be abbreviated.
Response: Whenever possible, we have now abbreviated the notions, as reported in supplementary figures 2-5 and supplementary Notes. Please check.

Selective sweep regions usually show lower nucleotide diversity and high level of haplotype homozygosity. Please delete the general statements about the selective sweep.
Response: We completely agree with the reviewer. The statement has been deleted accordingly.

What is meant by candidate selective sweep regions?
Response: They indicate genomic regions in which based on our analysis it would be more likely to identify signs of selective sweep. These regions show reduction of variation due to genetic hitchhiking with a site under selection. In other words, a selective sweep can occur when a rare or previously non-existing allele that increases the fitness of the carrier (relative to other members of the population) increases rapidly in frequency. A selective sweep due to a strongly selected allele, which arose on a single genomic background therefore results in a region of the genome with a large reduction of genetic variation in that chromosome region. Such detected regions were identified as selective regions. These selective regions containing genes which may be associated with specific phenotype, adaption, specific characters of a specie/breed/population, etc. Therefore, we called candidate selective regions.

Data need to be submitted in the public domain
Response: We have submitted the data to the NCBI Short Read Archive under the BioProject accession

number PRJNA547460. We have now added the information in the main text (Line 97-99), please check.

Reviewer #2:
In this manuscript Sun et al. analysed 121 buffalo whole genomes, out of which 98 genomes are newly reported. The authors provide a detailed description of the genetic diversity both addressing demographic questions and investigating selection signals. First, the authors assessed the split time between the ancestor of swamp and river buffalo and described the possible domestication scenarios for both of them. Moreover, they analysed uniparental markers which support their previous results even though they did not have mtDNA sequences for river buffalos. Finally, the authors investigated possible signals of selection using different approaches and they identified distinctive genes under selection in river and swamp buffalo.
Overall, the manuscript represents a comprehensive and detailed description of the genetic diversity, demographic histories and selection signals for river and swamp buffalo. I am happy with both the data generation and the analyses. Multiple approaches have also been used to confirm their results. However, I personally feel that the authors reported too many technical details regarding the amount the data generated, specifically in the paragraph titled "Data Description". I would rather move these technical details to the supplementary information and incorporate the estimates of genetic diversity into the next paragraph called "Analysis".
Response: Thanks for your kind suggestion. Due to the "Data Description" is a needed part according to the guideline of GigaScience. So, we only retain the brief information in the main text (line 96 - 101), and other information removed to the supplementary (supplementary: line 39 -line 45). Please check.

Considering the interesting results of this study, I would suggest the authors to highlight the main messages of the story around swamp and river buffalos and leave the more technical and descriptive sections to the supplementary information. The current version of the manuscript is a precise detailed description of all analyses performed but it would be nice to have the manuscript more structured and centred around the interesting story of the river and swamp buffalos. The authors could restructure some paragraphs focusing more on the main messages of this study and providing the analyses performed as evidence to support such messages. In this way the paper would be more engaging and easier to read.
Response: Thanks for your kind suggestion. We have revised the manuscript in order to make it more fluent, therefore some information has been moved to the supplementary material (Line 39-57) as suggested. We also tried our best to restructure some paragraphs (Line 244- Line 258, Line 274-279, Line 197-302), please check.

Regarding the mtDNA: I would like the authors to clarify why they were not able to get mtDNA sequences from the river buffalo genomes as they average coverage is pretty good. Possibly I misread the supplementary tables but some of samples of river buffalo, for examples, from India and Murrah have coverage between 9x and ~20x (from supplementary table 1).
Response: We really thank the reviewer for this advice. In previous analyses, we mapped the sequencing reads only on the swamp mitochondrial reference sequence (NC_006295.1). The results showed so many 'N' in the assembled river sequences that we didn't perform further analyses using the reconstructed river mtDNAs. Now, we mapped the sequencing reads of river buffaloes on the specific river mitochondrial genome (AF547270.1). Therefore, we were able to obtain high quality riverine mitochondrial genomes (Line 160-163) that allowed us to construct the phylogenetic tree (Supplementary figure 7) and network (Figure 2).

Minor points:
Supplementary tables: please report the tag "river" and "swamp" in all tables to make it easier to understand which samples belong to which categories instead of cross check multiple tables every time.
Response: We have revised, please check.
Line 88: I would suggest the author to change the word remainder referring to Indonesia as not appropriate.
Response: We have changed "the remainder of Indonesia" to "the rest of Indonesia", please check (Line 89).
Supplementary Table 3 is mentioned for the first time after Supplementary Table 4.
Response: We have now resolved this inconsistency, please check.

Close
