# Supplementary material for: Genomic analyses reveal distinct genetic architectures and selective pressures in buffaloes
Source: Gigascience. 2020 Feb 21;9(2):giz166. doi: 10.1093/gigascience/giz166 (PMC7033652; doi:10.1093/gigascience/giz166)
Supplement: giz166_Reviewer_2_Report_Original_Submission — Pierpaolo Maisano Delser -- 9/18/2019 Reviewed [file giz166_reviewer_2_report_original_submission.pdf]

Reviewer Report

Title: Genomic Analyses Reveal Distinct Genetic Architectures and Selective Pressures in Buffaloes

Version: Original Submission **Date:** 9/18/2019

Reviewer name: Pierpaolo Maisano Delser

Reviewer Comments to Author:

In this manuscript Sun et al. analysed 121 buffalo whole genomes, out of which 98 genomes are newly reported. The authors provide a detailed description of the genetic diversity both addressing demographic questions and investigating selection signals. First, the authors assessed the split time between the ancestor of swamp and river buffalo and described the possible domestication scenarios for both of them. Moreover, they analysed uniparental markers which support their previous results even though they did not have mtDNA sequences for river buffalos. Finally, the authors investigated possible signals of selection using different approaches and they identified distinctive genes under selection in river and swam buffalo.

Overall, the manuscript represents a comprehensive and detailed description of the genetic diversity, demographic histories and selection signals for river and swamp buffalo. I am happy with both the data generation and the analyses. Multiple approaches have also been used to confirm their results. However, I personally feel that the authors reported too many technical details regarding the amount the data generated, specifically in the paragraph titled "Data Description". I would rather move these technical details to the supplementary information and incorporate the estimates of genetic diversity into the next paragraph called "Analysis".

Considering the interesting results of this study, I would suggest the authors to highlight the main messages of the story around swamp and river buffalos and leave the more technical and descriptive sections to the supplementary information. The current version of the manuscript is a precise detailed description of all analyses performed but it would be nice to have the manuscript more structured and centred around the interesting story of the river and swamp buffalos. The authors could restructure some paragraphs focusing more on the main messages of this study and providing the analyses performed as evidence to support such messages. In this way the paper would be more engaging and easier to read.

Regarding the mtDNA: I would like the authors to clarify why they were not able to get mtDNA sequences from the river buffalo genomes as they average coverage is pretty good. Possibly I misread the supplementary tables but some of samples of river buffalo, for examples, from India and Murrah have coverage between 9x and ~20x (from supplementary table 1).

Minor points:

Supplementary tables: please report the tag "river" and "swamp" in all tables to make it easier to understand which samples belong to which categories instead of cross check multiple tables every time.

Line 88: I would suggest the author to change the word remainder referring to Indonesia as not appropriate.

Supplementary Table 3 is mentioned for the first time after Supplementary Table 4.

Methods

Are the methods appropriate to the aims of the study, are they well described, and are necessary controls included? Choose an item.

Conclusions

Are the conclusions adequately supported by the data shown? Choose an item.

Reporting Standards

Does the manuscript adhere to the journal's guidelines on minimum standards of reporting? Choose an item.

Choose an item.

Statistics

Are you able to assess all statistics in the manuscript, including the appropriateness of statistical tests used? Choose an item.

Quality of Written English

Please indicate the quality of language in the manuscript: Choose an item.

Declaration of Competing Interests

Please complete a declaration of competing interests, considering the following questions:

- Have you in the past five years received reimbursements, fees, funding, or salary from an organisation that may in any way gain or lose financially from the publication of this manuscript, either now or in the future?
- Do you hold any stocks or shares in an organisation that may in any way gain or lose financially from the publication of this manuscript, either now or in the future?
- Do you hold or are you currently applying for any patents relating to the content of the manuscript?
- Have you received reimbursements, fees, funding, or salary from an organization that holds or has applied for patents relating to the content of the manuscript?
- Do you have any other financial competing interests?
- Do you have any non-financial competing interests in relation to this paper?

If you can answer no to all of the above, write 'I declare that I have no competing interests' below. If your reply is yes to any, please give details below.

'I declare that I have no competing interests

I agree to the open peer review policy of the journal. I understand that my name will be included on my report to the authors and, if the manuscript is accepted for publication, my named report including any attachments I upload will be posted on the website along with the authors' responses. I agree for my report to be made available under an Open Access Creative Commons CC-BY license (http://creativecommons.org/licenses/by/4.0/). I understand that any comments which I do not wish to be included in my named report can be included as confidential comments to the editors, which will not be published.

Choose an item.

To further support our reviewers, we have joined with Publons, where you can gain additional credit to further highlight your hard work (see: https://publons.com/journal/530/gigascience). On publication of this paper, your review will be automatically added to Publons, you can then choose whether or not to claim your Publons credit. I understand this statement.

Yes Choose an item.
